# Genomic Instability Promotes the Progression of Clear Cell Renal Cell Carcinoma Through Influencing the Immune Microenvironment

**DOI:** 10.3389/fgene.2021.706661

**Published:** 2021-10-12

**Authors:** Xiyi Wei, Yichun Wang, Chengjian Ji, Jiaocheng Luan, Liangyu Yao, Xi Zhang, Shuai Wang, Bing Yao, Chao Qin, Ninghong Song

**Affiliations:** ^1^The State Key Lab of Reproductive, Department of Urology, The First Affiliated Hospital of Nanjing Medical University, Nanjing, China; ^2^Department of Medical Genetics, Nanjing Medical University, Nanjing, China; ^3^The Affiliated Kezhou People’s Hospital of Nanjing Medical University, Kezhou, China

**Keywords:** genomic instability, lncRNAs, ccRCC, MNX1-AS1, immune atlas

## Abstract

**Background:** Long non-coding RNAs (lncRNAs) are now under discussion as novel promising biomarkers for clear cell renal cell carcinoma (ccRCC). However, the role of genomic instability-associated lncRNA signatures in tumors has not been thoroughly uncovered. The purpose of our study is to probe the role of genomic instability-derived lncRNA signature (GILncSig) and to further investigate the mechanism of genomic instability-mediated ccRCC progression.

**Methods:** The transcriptome data and somatic mutation profiles of ccRCC as well as clinical characteristics used in this study were obtained from The Cancer Genome Atlas database and Gene Expression Omnibus database. Lasso regression analysis was performed to construct the GILncSig. Gene set enrichment analysis (GSEA) was performed to elucidate the biological functions and relative pathways. CIBERSORT and EPIC algorithm were applied to calculate the proportion of immune cells in ccRCC. ESTIMATE algorithm was utilized to compute the immune microenvironment scores.

**Results:** In total, 148 novel genomic instability-derived lncRNAs in ccRCC were identified. Immediately, on the basis of univariate cox analysis and lasso analysis, a GILncSig was appraised, through which the patients were allocated into High-Risk and Low-Risk groups with significantly different characteristics and prognoses. In addition, we confirmed that the somatic mutation count, tumor mutation burden, and the expression of UBQLN4, which were ascertainably associated with genomic instability, were significantly correlated with the GILncSig, indicating its reliability as a measurement of the genomic instability. Furthermore, the efficiency of GILncSig in prognostic aspects was better than the single mutation gene in ccRCC. In addition, MNX1-AS1 was defined to be a potential biomarker characterized by strong correlation with clinical features. Moreover, GSEA results indicated that the IL6/JAK/STAT3/SIGNALING pathway could be considered as a potential mechanism of genomic instability to influence tumor progression. Besides, the immune microenvironment showed significant differences between the GS-like group and the GU-like group, which was specifically manifested as high expression of CTLA4, GITR, TNFSF14, and regulatory T cells (Tregs) as well as low expression of endothelial cells (ECs) in the GU-like group. Finally, the prognostic value and clinical relevance of GILncSig were verified in GEO datasets and other urinary tumors in TCGA dataset.

**Conclusion:** In conclusion, our study provided a new perspective for the role of lncRNAs in genomic instability and revealed that genomic instability may mediate tumor progression by affecting immunity. Besides, MNX1-AS1 played critical roles in promoting the progression of ccRCC, which may be a potential therapeutic target. What is more, the immune atlas of genomic instability was characterized by high expression of CTLA4, GITR, TNFSF14, and Tregs, and low expression of ECs.

## Highlights

- Collectively, our study provided a new insight into the role of genomic instability-derived lncRNAs in the progression of ccRCC, which may mediate tumor progression by affecting tumor immunity.
- A total of 148 novel genomic instability-derived lncRNAs in ccRCC were identified, among which MNX1-AS1 played a critical role in promoting the progression of ccRCC, which may be a potential therapeutic target.
- The IL6/JAK/STAT3/SIGNALING pathway was considered as a potential mechanism of genomic instability to influence tumor progression. What is more, the immune atlas of genomic instability was characterized by high expression of CTLA4, GITR, TNFSF14, and Tregs, and low expression of endothelial cells.

## Introduction

Renal cell carcinoma (RCC), a malignant tumor originating from the kidney epithelium, caused nearly 12,000 deaths annually worldwide (Ljungberg et al., 2015). Its incidence has been increasing in the past decade, comprising up to 2–3% of all newly diagnosed tumor cases (Ljungberg et al., 2015). The median survival time of patients with metastatic RCC was only 13 months, and the 5-year survival rate was less than 10% (Lin et al., 2016). Although great development has been achieved in screening, diagnosis, and various treatments, the clinical outcomes of advanced RCC remained unsatisfied (Garcia and Rini, 2007; Gulati and Vaishampayan, 2020). Therefore, in order to provide a better treatment for clear cell RCC (ccRCC) patients, it was urgent to obtain a deeper understanding of the progression mechanism of ccRCC.

Genomic instability has been claimed as a hallmark of cancer, which may serve as a prognostic marker of tumor patients (Negrini et al., 2010). Moreover, the accumulation of genomic instability was associated with malignant progression and prognosis (Ottini et al., 2006). Although the molecular basis of genomic instability remained blurry, previous findings have revealed that genomic instability had strong relationships with aberrant transcriptional and post-transcriptional regulation, indicating that genomic instability may be measured by molecular signature. Many studies have been conducted to analyze the genomic instability signature in various cancers. For instance, the 12-gene genomic instability signature was defined by Habermann et al. (2009) to identify prognostic subtypes of breast cancer. It should be noted that the expansion of the biological understanding for non-coding RNAs (ncRNAs) has revealed its important roles in the process of tumorigenesis and progression. Meanwhile, the aberrant expression of long non-coding RNAs (lncRNAs) may have an impact on tumor progression or metastasis (Arun et al., 2016). Therefore, a novel mutator hypothesis-derived computational frame has been proposed, which combined lncRNA and genomic instability for predicting the prognosis of the patient (Bao et al., 2020).

Here, we described a genomic instability-derived lncRNAs signature based on the mutator hypothesis derived computational frame to predict the prognosis of ccRCC patients. In addition, the mechanism of genomic instability-mediated tumor progression was explored based on the lncRNA signature, which may provide a new perspective on how genomic instability influences the prognosis of ccRCC.

## Materials and Methods

### Acquisition of Data

The transcriptome file, lncRNA expression matrix, and somatic mutation information of patients with ccRCC were collected from The Cancer Genome Atlas (TCGA) database^1^, including 72 normal cases and 539 tumor cases. Meanwhile, corresponding clinical data were also obtained (Table 1). Fourteen cases were deleted due to lack of clinical information. All of the ccRCC patients included in this study were clustered into two cohorts according to the mutation characteristics, named GS-like group and GU-like group separately (Supplementary Table 1). For the purpose of establishing the prognostic model, the patients were divided into two sets. The training set consisted of 264 patients, which were used to identify prognostic lncRNA signatures and build a prognostic risk model, and the testing set contained 261 patients, which were used to independently validate the performance of the prognostic risk model (Table 1). Another two independent ccRCC validation datasets [GSE73731 (Wei et al., 2017) with 265 samples and GSE53757 (Herrem et al., 2005) with 144 samples] and corresponding clinicopathological information were obtained from the Gene Expression Omnibus (GEO) database^2^.

**TABLE 1:** Clinical characteristics of included patients in the study.

Variables	Total (*n* = 525)	Training cohort (*n* = 264)	Validation cohort (*n* = 261)
**Age (year)**			
<65	347(66.1%)	174(65.91%)	173(66.28%)
≥65	178(33.9%)	90(34.09%)	88(33.72%)
**Gender**			
FEMALE	182(34.67%)	100(37.88%)	82(31.42%)
MALE	343(65.33%)	164(62.12%)	179(68.58%)
**Stage**			
I	261(49.71%)	133(50.38%)	128(49.04%)
II	56(10.67%)	31(11.74%)	25(9.58%)
III	123(23.43%)	59(22.35%)	64(24.52%)
IV	82(15.62%)	40(15.15%)	42(16.09%)
Unknow	3(0.57%)	1(0.38%)	2(0.77%)
**T stage**			
T1	267(50.86%)	136(51.52%)	131(50.19%)
T2	68(12.95%)	40(15.15%)	28(10.73%)
T3	179(34.1%)	82(31.06%)	97(37.16%)
T4	11(2.1%)	6(2.27%)	5(1.92%)
**N stage**			
N0	237(45.14%)	127(48.11%)	110(42.15%)
N1	16(3.05%)	11(4.17%)	5(1.92%)
NX	272(51.81%)	126(47.73%)	146(55.94%)
**M stage**			
M0	417(79.43%)	214(81.06%)	203(77.78%)
M1	78(14.86%)	38(14.39%)	40(15.33%)
MX	28(5.33%)	10(3.79%)	18(6.9%)
Unknow	2(0.38%)	2(0.76%)	0(0%)
**Grade**			
G1	13(2.48%)	7(2.65%)	6(2.3%)
G2	226(43.05%)	111(42.05%)	115(44.06%)
G3	204(38.86%)	103(39.02%)	101(38.7%)
G4	74(14.1%)	38(14.39%)	36(13.79%)
GX	5(0.95%)	2(0.76%)	3(1.15%)
Unknow	3(0.57%)	3(1.14%)	0(0%)

### Identification of Differential Expression Genomic Instability-Derived Long Non-coding RNAs

In order to identify genomic instability-derived lncRNAs, a computational frame that was proposed in a previous study was used in this research. This process can be divided into five steps (Supplementary Figure 1): (i) the total number of somatic mutations in each patient was calculated; (ii) patients were ranked in decreasing order of the cumulative number of somatic mutations and divided into four parts; (iii) the top 25% of patients with the highest somatic mutations were defined as the high mutation (HM) group, and the last 25% with the lowest somatic mutations were defined as the low mutation (LM) group; (iv) lncRNA expression matrix between the HM group and the LM group was compared by using Wilcoxon test; (v) |LogFC| > 1.5 and p < 0.05 were used as the criteria for screening differentially expressed lncRNAs.

### Gene Ontology, KEGG Pathway, and Gene Set Enrichment Analysis

Pearson correlation coefficients (Pripp, 2018) were computed to measure the correlation between the paired expression of lncRNAs and mRNAs, and the top 10 mRNAs with the strongest correlation with the paired lncRNAs were selected as the co-expressed partners. In order to predict the potential functions of lncRNAs, GO enrichment analysis was conducted to comprehend the biological process and molecular function of the mRNAs, while KEGG enrichment analysis was applied to identify potential related biological pathways. Besides, gene set enrichment analysis (GSEA) was performed in order to probe the biological pathways associated with lncRNAs in genomic instability-derived lncRNA signature (GILncSig).

### Assessment of Immune Infiltrating Cells

CIBERSORT (Chen et al., 2018) was a deconvolution algorithm that used 547 tag gene expression values to characterize the composition of immune cells in tissues. In order to assess the association between genomic instability and immunity, this algorithm was applied to estimate the relative proportion of 22 immune infiltrating cells in ccRCC patients. We uploaded the corrected transcriptome data to the CIBERSORT website^3^ and set the algorithm to 1,000 rows. p < 0.05 was used as the criteria.

### Assessment of Stromal Cells

The immune score and stromal score that contained all stromal cells, including cancer-associated fibroblasts (CAFs), endothelial cells (ECs), mesenchymal stem cells (MCSs), and pericytes, were calculated by ESTIMATE algorithm (Yoshihara et al., 2013). By uploading the corrected gene expression data that were normalized by “limma” package to the EPIC website^4^, we obtained the proportions of CAFs, MCSs, and ECs in ccRCC patients.

### Statistical Analysis

According to the genomic instability-derived lncRNAs that were identified above, hierarchical cluster analysis was performed using Euclidean distances. Genomic instability-derived lncRNAs, which were significantly associated with survival, were identified by using univariate cox proportional hazard regression. For predicting the outcomes of ccRCC patients, lasso regression analysis was performed to estimate a GILncSig, which can be described as follows:


$$\text{GILncSig}\left(\text{patients}\right)=\sum_{i=1}^{n}\exp{\left(\ln\mathrm{cRNAi}\right)}^{*}\text{coef}\left(\ln\text{cRNAi}\right).$$

Exp (lncRNAi) represented the expression level of lncRNAi for the patients, and coef (lncRNAi) represented the contribution of lncRNAi to GILncSig, which was obtained from the regression coefficient of lasso regression analysis. The patients in the training set were divided into a High-Risk group with high GILncSig and a Low-Risk group with low GILncSig by using the median scores calculated by the GILncSig model. The survival rate and median survival for each prognostic risk group was calculated by using the Kaplan–Meier method, and the log-rank test was used to assess the difference in survival between two groups with a criteria level of *p* < 0.05. Multivariate cox regression was used to assess the independence of GILncSig compared with other clinical factors like T, M, N, and Stage. Hazard ratio (HR) and 95% confidence interval (CI) were calculated by cox analysis. The credibility and predictive value of the GILncSig were evaluated through a time-dependent receiver operating characteristic (ROC) curve. Tumor mutation burden (TMB; Goodman et al., 2017) was also calculated to evaluate the correlation between GILncSig and genomic instability. In addition, Wilcoxon test was used to assess the association between the expression of lncRNAs in GILncSig and immune microenvironment, while Pearson correlation coefficient was applied to calculate the correlation between lncRNAs and immune-related characteristics including immune checkpoints (Dyck and Mills, 2017) and immune cells. All statistical analyses were performed using R-version 3.6.0.

## Results

### Identification of Genomic Instability-Derived Long Non-coding RNAs in Clear Cell Renal Cell Carcinoma Patients

**FIGURE 1:**
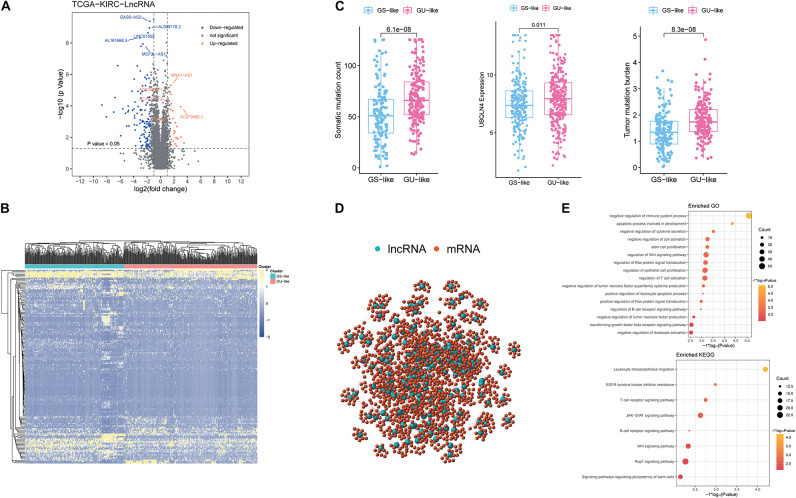
Identification and functional annotations of genomic instability-related lncRNAs in patients with ccRCC. **(A)** Volcano plot of differentially expressed lncRNAs between the GU-like group and the GS-like group. The right orange labeled lncRNAs were the expression of lncRNAs, which were significantly higher in the GU-like group than the GS-like group, and the left blue labeled lncRNAs were the expression of lncRNAs, which were significantly lower in the GU-like group than the GS-like group. |LogFC| > 1.5 and p < 0.05 were used as the criteria for screening differentially expressed lncRNAs. **(B)** Unsupervised clustering of 525 ccRCC patients based on the expression pattern of 148 candidate genomic instability-derived lncRNAs. The left blue cluster was the GS-like group, and the right orange cluster was the GU-like group. **(C)** Boxplots of somatic mutations count, UBQLN4 expression level, and tumor mutation burden in the GU-like group and GS-like group. Somatic mutations count, UBQLN4 expression level, and tumor mutation burden in the GU-like group were significantly higher than those in the GS-like group. Horizontal lines: median values. Statistical analysis was performed using the Mann–Whitney U test. **(D)** Co-expression network of genomic instability-related lncRNAs and mRNAs based on the Pearson correlation coefficient. The red circles represented mRNAs, and the blue circles represented lncRNAs. **(E)** Functional enrichment analysis of GO and KEGG for lncRNAs co-expressed mRNAs.

According to the cumulative numbers of somatic mutations in each patient, the top 25% (*n* = 84) of the samples with the highest somatic mutations were considered to be the HM group and the last 25% (*n* = 84) of the samples with the lowest somatic mutations were considered to be the LM group (Supplementary Figure 1 and Supplementary Table 1). Through Wilcoxon test, 148 novel genomic instability-derived lncRNAs in ccRCC were identified, including 126 down-regulated lncRNAs and 22 up-regulated lncRNAs (Figure 1A and Supplementary Table 2). By conducting unsupervised hierarchical clustering analysis, 539 ccRCC samples were clustered into two groups according to the expression levels of the 148 differentially expressed lncRNAs (Figure 1B and Supplementary Table 3). The group with higher cumulative somatic mutations was defined as the GU-like group, and the other group was defined as the GS-like group. The somatic mutation counts, UBQLN4 and TMB, which were newly identified as drivers of genomic instability (Dyck and Mills, 2017), were significantly differentially expressed between the two groups (*p* < 0.05; Figure 1C). In order to predict the potential functions of the identified lncRNAs, we calculated the Pearson correlation coefficients between the paired lncRNAs and target mRNAs (Supplementary Table 4). The top 10 protein coding genes with the strongest correlation with the paired lncRNAs were selected as the co-expressed partners. A lncRNA–mRNA co-expression network was constructed where the nodes were lncRNAs and mRNAs (Figure 1D). GO analysis revealed that the GO terms of the mRNAs in this network were significantly associated with immune-associated pathways including negative regulation of cytokine secretion, negative regulation of immune system process, regulation of WNT signaling pathway, negative regulation of tumor necrosis factor superfamily cytokine production, regulation of B-cell receptor signaling pathway, and negative regulation of leukocyte activation. In addition, KEGG analysis revealed that the prominent enriched pathways for co-expression mRNAs were T-cell receptor signaling pathway, WNT signaling pathway, B-cell receptor signaling pathway, and signaling pathways regulating pluripotency of stem cells, among others (Figure 1E). These pathways indicated that the 148 differentially expressed genomic instability-derived lncRNAs had a strong correlation with immunity, which indicated that genomic instability may mediate tumor progression through immune mechanism.

### Establishment and Evaluation of a Genomic Instability-Derived Long Non-coding RNAs Signature in the Training and Validation Sets

**FIGURE 2:**
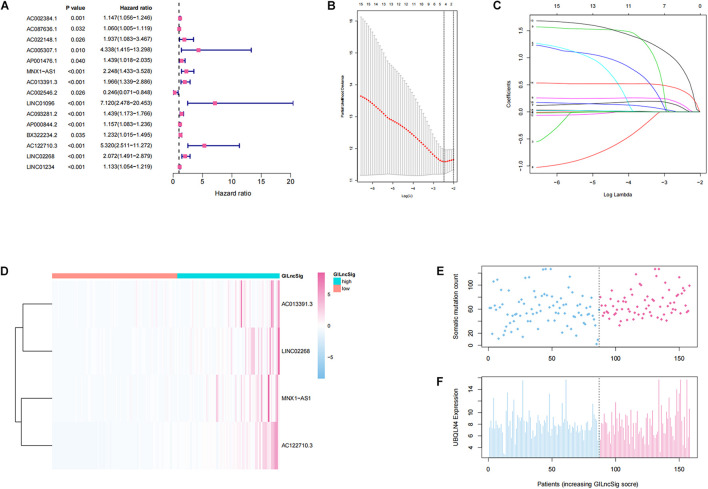
Establishment and identification of the genomic instability-derived lncRNA signature (GILncSig) for outcome prediction in the training set. **(A)** Forest plot of 15 lncRNAs, which has a significant association with the prognosis of ccRCC. **(B,C)** Lasso regression model for 15 prognostic lncRNAs used to construct GILncSig. **(D)** Heatmap of lncRNAs in GILncSig between the high-GILncSig and low-GILncSig groups (divided by median value) in the training set. **(E)** Distribution of somatic mutation counts in ccRCC patients. **(F)** Distribution of the expression level of UBQLN4 in ccRCC patients.

In order to investigate the prognostic effects of these candidate genomic instability-derived lncRNAs, 525 ccRCC patients from the TCGA database combined with clinical information were divided into the training set (*n* = 264) and the testing set (*n* = 261; Table 1). Here, some cases were removed due to lack of clinical information. Among the identified 148 differentially expressed lncRNAs, 15 lncRNAs that had significant associations with the prognosis of ccRCC patients were selected through univariate cox regression analyses (*p* < 0.05; Figure 2A). Then, lasso regression analysis was applied to construct a prognostic model (Figures 2B,C). Finally, we got four lncRNAs (LINC02268, MNX1-AS1, AC013391.3, and AC122710.3; Figure 2D). Then, a GILncSig was constructed to assess the prognosis risk of ccRCC patients based on the coefficients of lasso regression analysis and the expression level of four independent prognostic genomic instability-derived lncRNAs as follows:


GILncSig=(0.1225*⁢MNX1-AS1)+(0.0673*⁢AC⁢013391.3)+(0.7766*⁢AC122710⁢.3)+(0.3861*⁢LINC⁢02268).

**FIGURE 3:**
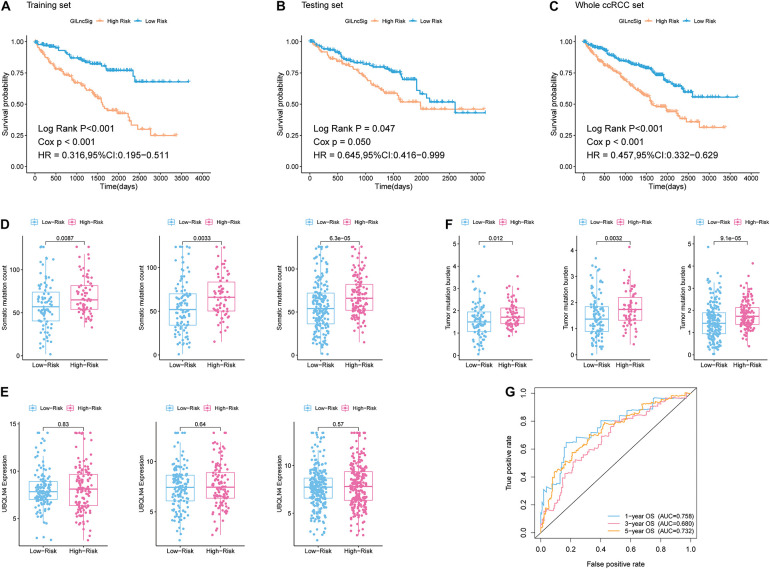
Identification of the predictive efficacy of the model. **(A)** Kaplan–Meier curves of overall survival of patients with low or high risk predicted by the GILncSig in the training set. **(B)** Kaplan–Meier curves of overall survival of patients with low or high risk predicted by the GILncSig in the testing set. **(C)** Kaplan–Meier curves of overall survival of patients with low or high risk predicted by the GILncSig in the whole ccRCC set. **(D)** Boxplots of somatic mutation count in the High-Risk group and Low-Risk group in three sample sets. Somatic cumulative mutations in the High-Risk group were significantly higher than those in the GS-like group. **(E)** Boxplots of UBQLN4 expression level in the High-Risk group and Low-Risk group in three sample sets. The expression level of UBQLN4 in the High-Risk group showed no difference compared with that in the Low-Risk group. **(F)** Boxplots of tumor mutation burden in the High-Risk group and Low-Risk group in three sample sets. Tumor mutation burden in the High-Risk group was significantly higher than that in the Low-Risk group. **(G)** Time-dependent ROC curve analysis of the GILncSig at 1, 3, and 5 years.

In addition, patients in the training group and validation group were separated into different prognostic groups *via* the median GILncSig score (0.074) as a threshold. The distribution of somatic mutation counts and the expression level of UBQLN4 in ccRCC was displayed (Figures 2E,F). The results from the K–M analysis indicated that high-risk patients had lower overall survival than low-risk patients in both the training group and the validation group (*p* < 0.05, Figures 3A–C). According to group information, we observed that the higher counts of somatic mutations and high TMB significantly corresponded with the high-risk type while there was no significant difference in the expression of UBQLN4 between the two groups (*p* < 0.05; Figures 3D–F). Besides, four lncRNAs in GILncSig showed significant correlation with TMB, which indicated that these lncRNAs significantly related to genomic instability (*p* < 0.05, Supplementary Figures 2A–D). The ROC curve prompted that the GILncSig had dominant credibility and predictive value in the training set (1-year os AUC = 0.758, 3-year os AUC = 0.680, and 5-year os AUC = 0.732; Figure 3G).

### Independent Validation of Genomic Instability-Derived Long Non-coding RNAs Signature in the Clear Cell Renal Cell Carcinoma Dataset With RNA-seq Platform and the External Gene Expression Omnibus Dataset With Microarray Platform

**FIGURE 4:**
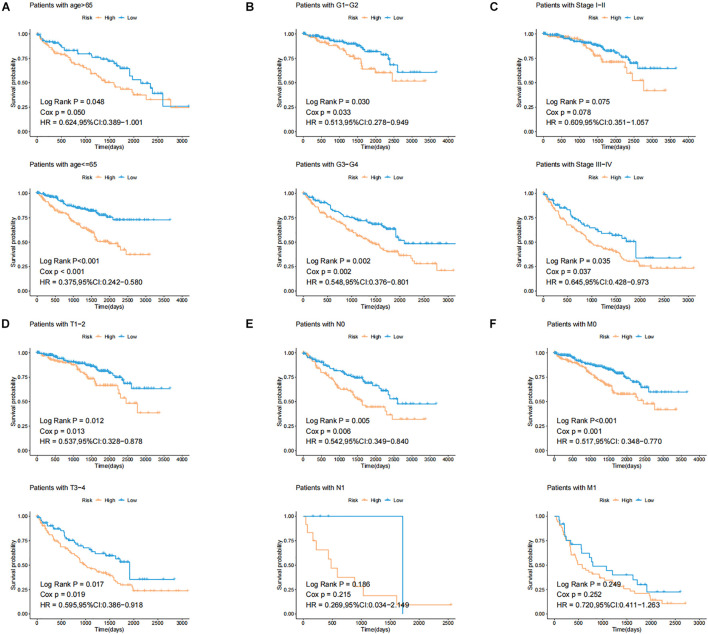
Identification of the efficiency of GILncSig in specific clinical samples. **(A)** Kaplan–Meier curves of overall survival of patients with low or high risk predicted by the GILncSig in patients with Age < 65 and Age ≥ 65. **(B)** Kaplan–Meier curves of overall survival of patients with low or high risk predicted by the GILncSig in patients with G1-2 and G3-4. **(C)** Kaplan–Meier curves of overall survival of patients with low or high risk predicted by the GILncSig in patients with Stage I–II and Stage III–IV. **(D)** Kaplan–Meier curves of overall survival of patients with low or high risk predicted by the GILncSig in patients with T1-2 and T3-4. **(E)** Kaplan–Meier curves of overall survival of patients with low or high risk predicted by the GILncSig in patients with N0 and N1. **(F)** Kaplan–Meier curves of overall survival of patients with low or high risk predicted by the GILncSig in patients with M0 and M1.

**FIGURE 5:**
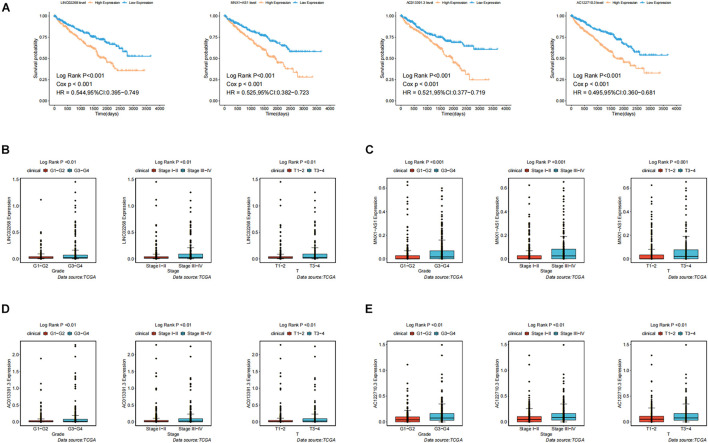
Identification of the clinical characteristics and overall survival of genomic instability-derived lncRNAs in GILncSig. **(A)** Kaplan–Meier curves of overall survival of MNX1-AS1, AC013391.3, AC122710.3, and LINC02268. **(B)** Boxplots of LINC02268 expression level in different clinical factors like Grade, T stage, and Stage. The expression level of LINC02268 was significantly associated with poorer prognosis and the increasing level of clinical factors. **(C)** The expression level of LINC02268 was significantly associated with poorer prognosis and the increasing level of clinical factors. **(D)** The expression level of AC013391.3 was significantly associated with poorer prognosis and the increasing level of clinical factors. **(E)** The expression level of AC122710.3 was significantly associated with poorer prognosis and the increasing level of clinical factors.

In order to test the robustness of the GILncSig, the prognostic performance of GILncSig was tested by the independent TCGA testing set (*n* = 261) and the total TCGA validation set (*n* = 525). By constructing univariate analysis, the HR of the High-Risk group versus the Low-Risk group for overall survival in the whole validation set was 4.893 (95% CI: 2.799–8.553, *p* < 0.05, Table 2). To assess whether GILncSig was independent of other clinical features, we divided the clinical information into different types, including Age < 65 and Age ≥ 65, FEMALE and MALE, G1–G2 and G3–G4, M0 and M1, N0 and N1, Stage I–II and Stage III–IV, and T1–2 and T3–4. We found that GILncSig can effectively divide patients into high and low survival groups among patients from different ages and stages, indicating independent predictive power (Figures 4A–F). By constructing Kaplan–Meier analysis, we found that all of these four lncRNAs were risk factors because their high expression were associated with poor prognosis, which corresponded to coefficients in GILncSig (Figure 5A). To estimate the correlation between four lncRNAs and clinical features, Wilcoxon test and Kruskal–Wallis test were involved. When clinical traits had two characteristics, Wilcoxon test was used to examine the correlation between the expression of the these lncRNAs and clinical features, and Kruskal–Wallis test was applied when the clinical features had more than two characteristics. The result suggested that LINC02268, MNX1-AS1, AC013391.3, and AC122710.3 were associated with the progression of tumors where it showed significant correlation with grade, stage, and T stage (*p* < 0.05, Figures 5B–E).

**TABLE 2:** Univariate and Multivariate cox regression analysis of the GILncSig and overall survival in different patient sets.

Variables	Univariate analysis			Multivariate analysis		
	HR	95% CI	P-Value	HR	95% CI	P-Value
**TCGA set (n = 525)**						
Age	1.565	1.147–2.136	0.004	1.525	1.115–2.084	0.008
Gender	0.957	0.694–1.318	0.789			
Grade	2.667	1.870–3.802	0.000	1.655	1.136–2.411	0.008
Stage	4.250	3.047–5.928	0.000	3.328	1.716–6.457	0.000
T	3.415	2.488–4.686	0.000	0.890	0.483–1.639	0.709
M	2.144	1.695–2.712	0.000	1.546	1.160–2.061	0.002
N	0.859	0.735–1.004	0.056			
RiskScore	4.893	2.799–8.553	0.000	1.898	1.002–3.595	0.049
**Train set (n = 264)**						
Age	1.537	0.995–2.374	0.052			
Gender	1.155	0.741–1.799	0.524			
Grade	2.725	1.659–4.474	0.000	1.654	0.968–2.827	0.065
Stage	4.508	2.845–7.141	0.000	2.604	1.108–6.121	0.028
T	3.475	2.242–5.386	0.000	1.170	0.529–2.588	0.697
M	2.383	1.701–3.338	0.000	1.605	1.046–2.463	0.030
N	0.830	0.663–1.039	0.104			
RiskScore	7.304	3.823–13.95	0.000	2.905	1.322–6.387	0.007
**Test set (n = 261)**						
Age	1.575	1.009–2.460	0.045	1.518	0.963–2.392	0.071
Gender	0.763	0.479–1.216	0.256			
Grade	2.611	1.572–4.339	0.000	1.677	0.980–2.868	0.058
Stage	4.046	2.488–6.578	0.000	3.277	1.025–10.48	0.045
T	3.423	2.149–5.453	0.000	0.913	0.312–2.667	0.868
M	1.991	1.430–2.771	0.000	1.463	0.969–2.208	0.069
N	0.895	0.716–1.118	0.329			
RiskScore	2.241	0.723–6.940	0.161			

### The Efficiency of Genomic Instability-Derived Long Non-coding RNAs Signature Was Better Than That of the Single Mutation Gene

**FIGURE 6:**
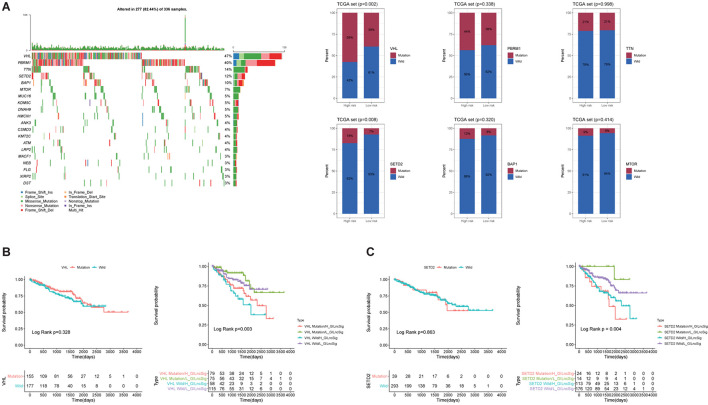
Identification of the mutation characteristics and overall survival in different mutation types. **(A)** Waterfall plot of the top 20 most frequently mutated genes in ccRCC samples and barplots of the top 20 most frequently mutated genes in the whole TCGA ccRCC sample. Only the proportion of patients with VHL and SETD2 mutations in the High-Risk group was significantly higher than that in the Low-Risk group among the TCGA set. **(B)** Kaplan–Meier curves of patients with different combination of GILncSig and VHL mutation. **(C)** Kaplan-Maier curves of patients with different combination of GILncSig and SETD2 mutation.

To evaluate the prognostic efficacy of GILncSig and high-frequency mutation genes, we selected the top six most frequently mutated genes, including VHL, PBRM1, TTN, SETD2, BAP1, and MTOR (Figure 6A). The result showed that the proportion of patients with VHL and SETD2 mutations in the High-Risk group was significantly higher than that in the Low-Risk group among the TCGA set (Figure 6A). In the TCGA set, the proportion of High-Risk group patients (58%) possessed significantly higher SETD2 mutations than the Low-Risk group (39%; *p* < 0.01). Similarly, the proportion of High-Risk group patients (18%) possessed significantly higher VHL mutations than the Low-Risk group (7%; *p* < 0.01). This result indicated that GILncSig may be a promising mutation marker. To further test whether the efficiency of GILncSig was better than VHL and SETD2 mutation status, we combined the GILncSig information with VHL and SETD2 mutation information. The TCGA set was divided into four groups, including VHL Mutation/H-GILncSig, VHL Mutation/L-GILncSig, VHL Wild/H-GILncSig, and VHL Wild/l-GILncSig. The survival curves of four groups demonstrated remarkable differences (*p* < 0.05, Figure 6B). The patients with combined VHL Mutation/L-GILncSig had significantly higher overall survival rate than the patients labeled VHL Mutation/H-GILncSig, while the patients with combined VHL Wild/L-GILncSig had significantly higher overall survival rate than the patients labeled VHL Wild/H-GILncSig. With regard to SETD2, consistent results were obtained (Figure 6C). Consequently, the GILncSig and the genomic instability information had greater prognostic efficiency than VHL or SETD2 mutation status alone.

### Gene Set Enrichment Analysis Uncovered That the IL6/JAK/STAT3/SIGNALING Pathway May Be a Potential Pathway for Explaining How Genomic Instability-Derived Long Non-coding RNAs Affected Tumor Progression

**FIGURE 7:**
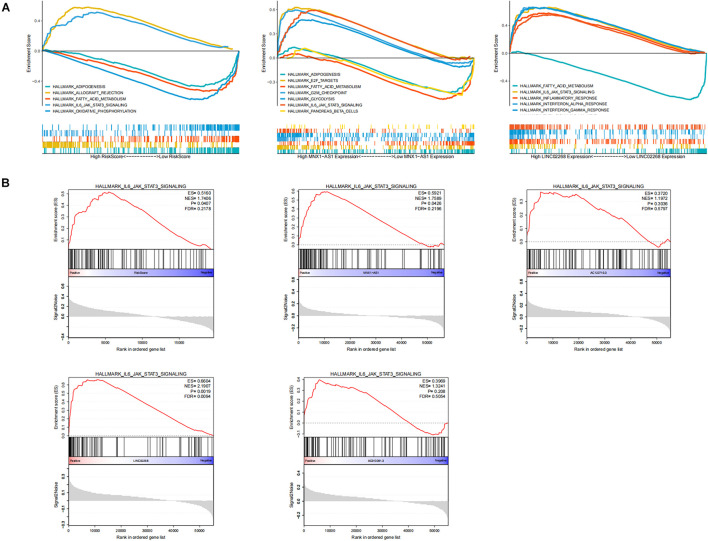
Identification of the biological functions of lncRNAs in GILncSig. **(A)** Multi-GSEA result of significant enrichment pathways about MNX1-AS1, LINC02268, and GILncSig. **(B)** The enrichment of the IL6/JAK/STAT3/SIGNALING pathway among MNX1-AS1, AC013391.3, AC122710.3, LINC02268, and GILncSig. The IL6/JAK/STAT3/SIGNALING pathway can be activated by MNX1-AS1, AC013391.3, AC122710.3, LINC02268, and GILncSig, while only MNX1-AS1, LINC02268, and GILncSig showed significant correlation (p < 0.05).

To explore the biological function of lncRNAs (LINC02268, MNX1-AS1, AC013391.3, and AC122710.3) and GILncSig in the progression of ccRCC, we performed GSEA analysis based on the TCGA cohort. Significant enrichment pathways of these LncRNAs were presented, in which no significant pathways were enriched by AC013391.3 and AC122710.3 (Figure 7A). Enrichment result indicated that the IL6/JAK/STAT3/SIGNALING pathway can be activated by MNX1-AS1, AC013391.3, AC122710.3, LINC02268, and GILncSig, while only MNX1-AS1, LINC02268, and GILncSig showed significant correlation (*p* < 0.05, Figure 7B). The IL6/JAK/STAT3/SIGNALING pathway has been proved that it was aberrantly hyperactivated in various types of cancer and had a strong relationship with poor clinical prognosis. In the tumor microenvironment, IL6/JAK/STAT3/SIGNALING played an important role in promoting the proliferation and metastasis of tumor while strongly inhibiting the antitumor immune response (Johnson et al., 2018). Interestingly, this result indicated that the IL6/JAK/STAT3/SIGNALING pathway may be a potential pathway for explaining how genomic instability-derived lncRNAs affected tumor progression.

### Genomic Instability Had a Strong Correlation With Checkpoints and Immune-Associated Cells

**FIGURE 8:**
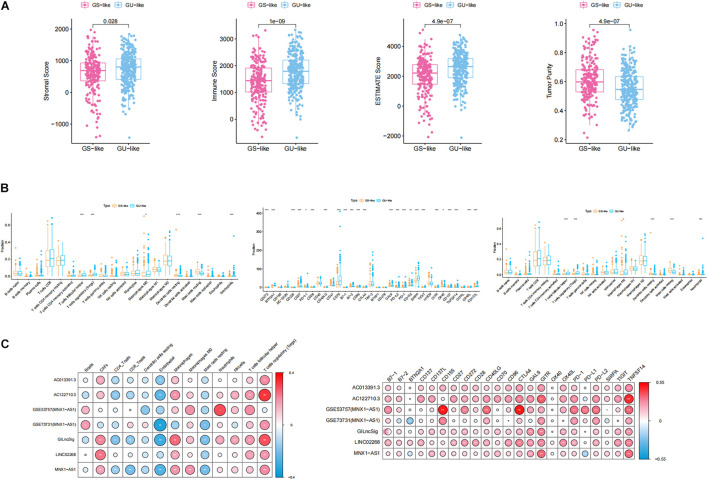
Identification of the characteristics of immune microenvironment and genomic instability and description of the immune atlas of genomic instability. **(A)** Boxplot of the expression level of ImmuneScore, ESTIMATE, StromalScore, and TumorPurity in the GU-like group and GS-like group. The expression level of ImmuneScore, ESTIMATE, and StromalScore in the GU-like group was significantly higher than that in the GS-like group. However, the expression level of TumorPurity in the GU-like group was significantly lower than that in the GS-like group. **(B)** Boxplot of the expression level of immune-associated cells and immune checkpoints in the GU-like group and GS-like group. **(C)** Correlation of immune cells and immune checkpoints with lncRNAs in TCGA samples and GEO samples. Regulatory T cells (Tregs), CTLA4, GITR, and TNFSF14 had a significant positive correlation with genomic instability characteristics and endothelial cells had a significant negative correlation with genomic instability characteristics.

For the purpose of analyzing whether genomic instability promoted the progression of ccRCC through affecting immunity, which was indicated by the GSEA, GO, and KEGG results, ESTIMATE algorithm was used to calculate immune scores, ESTIMATE scores, stromal scores, and tumor purity. As shown in Figure 8A, all of these scores displayed significant differences between GS-like and GU-like groups. Specifically, immune scores, ESTIMATE scores, and stromal scores shared the same phenomenon that the scores in the GU-like group were notably higher than those in the GS-like group, while tumor purity had a completely opposite tendency. This result suggested that the genomic instability may influence the prognosis of ccRCC by disturbing the tumor microenvironment. Then, we calculated the proportion of 22 types of immune infiltrating cells in ccRCC by uploading the normalized transcriptome data to the CIBERSORT website. Concretely, the proportion of follicular helper T cells, regulatory T cells (Tregs), macrophage M0, and neutrophils in the GU-like group were notably higher than those in the GS-like group, while in the GU-like group, resting dendritic cells and resting mast cells processed lower proportion than that in the GS-like group (Figure 8B). To further verify the association between genomic instability and immunity, we acquired the proportion of immune cells including CAFs, ECs, MCSs, and pericytes through the EPIC website. Wilcoxon test was employed to assess the association between genomic instability and immune cells. The result was consistent with previous findings that all cells were significantly differently infiltrated between the GU-like group and the GS-like group (Figure 8B). This phenomenon suggested that the GS-like group had a more stable immune microenvironment than the GU-like group. In addition, we examined the expression of immune checkpoints in different groups of ccRCC patients. Most of the checkpoints presented a significant difference between the GS-like group and the GU-like group, among which only BTN2A1 and OX40 showed lower expression in the GU-like group while 21 other checkpoints, namely CD272, CD226, CD27, CD28, CD40LG, CD70, B7-1, B7-2, CD96, CTLA4, GAL9, PD-L2, PD-1, CD155, SIRPA, TIGIT, GITR, CD137, TNFSF14, OX40L, and CD137L, shared higher expression in the GU-like group (Figure 8B). It was well known that immune checkpoints (Bruni et al., 2020) promoted tumor progression by suppressing the expression of immune cells, which may explain the reason why the GU-like group had worse survival rates than the GS-like group. Therefore, all evidence suggested that genomic instability revealed poor immune characteristics, and it may promote tumor progression. As a result, GU-like group patients had significantly lower overall survival rate than the GS-like patients, which verified the previous assumptions.

### Description of the Genomic Instability-Derived Immune Atlas

For the purpose of identifying the immune characteristic of genomic instability, Pearson correlation test was applied to calculate the correlation between genomic instability-derived lncRNAs and immune-associated cells as well as immune checkpoints. There were several immune-associated cells that showed a strong relationship with genomic instability, where Tregs had a significant positive correlation with genomic instability and ECs had a significant negative correlation with genomic instability. In addition, CTLA4, GITR, and TNFSF14 had a significant positive correlation with genomic instability (*p* < 0.05, Figure 8C). A cross-platform validation from GSE53757 and GSE73731 was involved to verify the correlation between genomic instability and these immune features, and the result was similar to the correlation in the TCGA set (Figure 8C). Specifically, CTLA4, GITR, TNFSF14, and Tregs showed higher expression in the GU-like group, while ECs showed lower expression in the GU-like group (Supplementary Figure 3). Finally, these results uncovered the immune atlas of genomic instability and provided ideas for subsequent immunotherapy.

### The Practicability of the Genomic Instability-Derived Long Non-coding RNAs Signature Was Verified by Other Urinary Tumors

**FIGURE 9:**
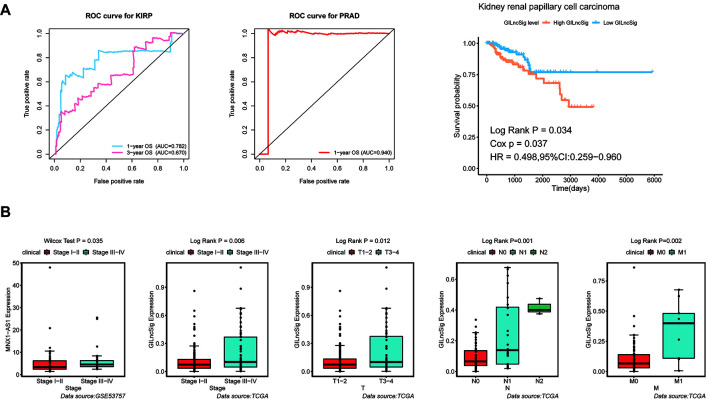
Identification of the practicability of the GILncSig in other urinary tumors. **(A)** Time-dependent ROC curve analysis of the GILncSig in KIRP at 1 and 3 years, time-dependent ROC curve analysis of the GILncSig in PRAD at 1 year, and Kaplan–Meier curves of overall survival of GILncSig in KIRP. **(B)** Boxplot of MNX1-AS1 expression level between stage I–II and stage III–IV in GSE53757 dataset and boxplots of GILncSig levels in different clinical subgroups in TCGA KIRP dataset. The expression level of MNX1-AS1 was significantly associated with increasing level of stage and GILncSig was significantly associated with increasing level of clinical factors containing T, M, N, and Stage.

For the purpose of verifying the broad applicability of GILncSig, lncRNA expression matrix and paired clinical information of patients with kidney renal papillary cell carcinoma (KIRP) and prostate adenocarcinoma (PRAD) were collected from the TCGA database. By using GILncSig, every patient’s GILncSig score was calculated. The ROC curve prompted that the GILncSig had dominant credibility and predictive value in the KIRP set (1-year os AUC = 0.782; 3-year os AUC = 0.670; Figure 9A). Meanwhile, the 1-year overall survival AUC of PRAD patients was 0.94. The results from the K–M analysis indicated that High-Risk patients had significantly lower overall survival than Low-Risk patients in KIRP (*p* < 0.05; Figure 9A). This result proved that GILncSig had wide practicability in urinary tumors. A cross-platform validation from GSE53757 was involved to verify the correlation between these lncRNAs and clinical features, in which the higher expression of MNX1-AS1 was significantly correlated with stage III–IV, which showed the same tendency with the result in the TCGA set (*p* < 0.05, Figure 9B). In conclusion, MNX1-AS1 may be a potential biological marker promoting tumor progression by affecting genomic instability. Moreover, GILncSig from KIRP patients shared the same clinical relevance with that from ccRCC patients (Figure 9B). The GILncSig scores increased gradually with the progress of clinical stages and showed significant differences.

## Discussion

With the development of scientific research, traditional histopathological features (tumor size, stage, and grade) may not satisfy the need for diagnosis and prognosis of ccRCC. Radical nephrectomy has been proved to be a definitive treatment for localized RCC, after which many patients may finally experience progression or recurrence (Garcia and Rini, 2007; Barata and Rini, 2017). Genomic instability has become the focus in recent years, which may serve as a molecular target in multiple tumors (Bartkova et al., 2005; Gorgoulis et al., 2005). In addition, lncRNAs, as novel biological markers, have been applied in many cancers (Gupta et al., 2010; Arun et al., 2016). However, many current studies were limited to the effects of genomic instability and lncRNA on tumor prognosis and ignore the potential mechanisms behind genomic instability and lncRNA (Zeng et al., 2019; Bao et al., 2020). Based on previous studies on lncRNA and genomic instability, we explored the effects of genomic instability-derived lncRNAs on tumor prognosis in depth and explored the potential mechanisms through which genomic instability promoted the progression of ccRCC.

In this study, we identified 148 novel genomic instability-derived lncRNAs by combining the lncRNA expression profile with the somatic mutation profile of ccRCC. By analyzing the function of target genes of genomic instability-derived lncRNAs, a majority of immune-associated pathways were enriched. Immune response can inhibit and promote the development and progression of tumor lesions through a process called immunoediting. Immunoediting during tumor progression was considered to be a three-steps process: elimination, balance, and escape. In the elimination stage, cancer cells can completely elude immune surveillance by using immunosuppressive signals, which promoted tumor growth and spread (Schreiber et al., 2011; O’Donnell et al., 2019). Consequently, genomic instability may mediate tumor progression through immunoediting. Similarly, Yang et al. developed a risk signature based on genomic instability-related lncRNAs for prognosis prediction and drug guidance in ccRCC. Their research provided new insights into the role of genomic instability-derived lncRNAs in ccRCC. In particular, in addition to the prognostic correlation, we paid more attention to the impact of genomic instability-derived lncRNAs on the immune microenvironment and provided targets for immunotherapy (Mirgayazova et al., 2019).

We further investigated whether genomic instability-derived lncRNAs can predict clinical outcomes and establish a GILncSig, including four genomic instability-derived lncRNAs (LINC02268, MNX1-AS1, AC013391.3, and AC122710.3). In addition, the significant correlation between GILncSig and tumor mutator phenotype, TMB, and UBQLN4 indicated that GILncSig can serve as a good indicator of genomic instability. Meanwhile, the practicability of the GILncSig was verified in the ccRCC dataset, GEO dataset, and other urinary tumors such as KIRP and PRAD. By performing clinical correlation analysis, we found that all of these four lncRNAs were associated with the progression of the tumor, which was consistent with previous results. Quite especially, MNX1-AS1 was verified by external data as a driver of ccRCC progression. Previous studies have found that MNX1-AS1 can be used as a prognostic indicator for patients with gastric cancer. MNX1-AS1 activated by TEAD4 can promote the GC process through EZH2/BTG2 and miR-6785-5p/BCL2 axes, suggesting that it was a novel and effective therapeutic target for gastric cancer (Shuai et al., 2020). Another study showed that MNX1-AS1 can promote the progression of intrahepatic cholangiocarcinoma through c-myc and Hippo pathways (Li J. et al., 2020). Therefore, current studies have shown that MNX1-AS1 was involved in the occurrence and development of a variety of malignant tumors (Chu et al., 2019; Liu et al., 2019; Li F. et al., 2020).

Through functional annotation and pathway enrichment analysis, the mechanisms of genomic instability-derived lncRNAs mediating ccRCC development were intuitively outlined. The result suggested that these lncRNAs were enriched in many different pathways, but IL6/JAK/STAT3/SIGNALING can be enriched by all of these four lncRNAs and GILncSig. It has been proved that the IL6/JAK/STAT3/SIGNALING pathway was aberrantly hyperactivated in various types of cancer that had a strong relationship with poor clinical prognosis. In the tumor microenvironment, IL6/JAK/STAT3/SIGNALING played an important role in promoting the proliferation and metastasis of tumor while strongly inhibiting the antitumor immune response (Johnson et al., 2018). IL-6 was produced by a variety of cell types located in the tumor microenvironment, including tumor-infiltrating immune cells, stromal cells, and tumor cells themselves (Walter et al., 2009; Nagasaki et al., 2014; Kumari et al., 2016). IL-6 directly acted on tumor cells and induced the increasing expression of STAT3 target genes. The proteins encoded by STAT3 target genes subsequently precipitated tumor proliferation (such as cyclin D1, BCL-xL). STAT3 promoted IL6 gene expression and led to a feedforward autocrine feedback loop. STAT3 also increased the expression of angiogenic factors, such as VEGF, matrix metalloproteinases, IL-10, and TGF-β (Yu and Jove, 2004; Yu et al., 2009). To summarize, genomic instability may enhance the progression and metastasis of ccRCC through activating the IL6/JAK/STAT3/SIGNALING pathway and suppressing immune response, which laid the foreshadowing for the follow-up research.

For the purpose of analyzing whether genomic instability promoted the progression of ccRCC through immunoediting, ESTIMATE, stromal score, and tumor purity were used to appraise the relationship between genomic instability and the immune microenvironment. Interestingly, the result suggested that the genomic instability may deteriorate the prognosis of ccRCC by disturbing the tumor immune microenvironment. Then, we acquired the proportion of immune cells and calculated the relationship between immune cells and genomic instability. The result suggested that the proportion of several immune cells was significantly infiltrated including follicular helper T cells, Tregs, macrophage M0, resting dendritic cells, resting mast cells, and neutrophils. Meanwhile, all cells calculated by the EPIC algorithm were differentially infiltrated between the GU-like group and the GS-like group. In addition, CTLA4, GITR, TNFSF14, and Tregs had a significant positive correlation with genomic instability. This result indicated that genomic instability portended poor immune characteristics and a complex immune microenvironment, which may account for the mechanism through which genomic instability promoted the progression of ccRCC.

Though our study provided important insights into the relationship between genome instability and the prognosis of ccRCC, it still had some limitations that required further study. For example, the mechanism by which genomic instability affected tumor immunity remained unclear. The specific mechanism needs further elucidation. However, our research provided a vital approach and a new perspective for the role of lncRNAs in genomic instability and revealed potential mechanisms through which genomic instability affected tumor progression.

## Conclusion

Based on mutator hypothesis-derived computational frame, a GILncSig was established as an independent prognostic marker to stratify risk subgroups for ccRCC patients, which was externally verified in GEO and other tumor cohorts. In addition, genomic instability was characterized by a complex immune environment. MNX1-AS1, CTLA4, GITR, TNFSF14, Tregs, and ECs were defined as therapeutic targets that could be used for describing the genomic instability-derived immune atlas. Moreover, IL6/JAK/STAT3/SIGNALING may be a potential pathway for explaining the way genomic instability reduced the overall survival rate of ccRCC patients.

## Data Availability Statement

The original contributions presented in the study are included in the article/Supplementary Material; further inquiries can be directed to the corresponding author/s.

## Author Contributions

NS, CQ, and BY designed this work. XW and YW wrote the manuscript. CJ performed the bioinformatics analysis. JL, LY, XZ, and SW performed the data review. All authors have read and approved the manuscript.

## Conflict of Interest

The authors declare that the research was conducted in the absence of any commercial or financial relationships that could be construed as a potential conflict of interest.

## Publisher’s Note

All claims expressed in this article are solely those of the authors and do not necessarily represent those of their affiliated organizations, or those of the publisher, the editors and the reviewers. Any product that may be evaluated in this article, or claim that may be made by its manufacturer, is not guaranteed or endorsed by the publisher.

## Footnotes

1
https://portal.gdc.cancer.gov/

2
https://www.ncbi.nlm.nih.gov/geo/

3
http://cibersort.stanford.edu/

4
http://epic.gfellerlab.org/

## Supplementary Material

Supplementary Figure 1 The flow diagram of the study.

Supplementary Figure 2 Identification the correlation between genomic instability-derived lncRNAs and Tumor mutation burden (TMB). **(A)** The correlation between LINC02268 and TMB. **(B)** The correlation between MNX1-AS1 and TMB. **(C)** The correlation between AC013391.3 and TMB. **(D)** The correlation between AC122710.3 and TMB.

Supplementary Figure 3 Identification the expression level of the immune-associated features in GU-like group and GS-like group. The expression level of T cells regulatory (Tregs), CTLA4, GITR, and TNFSF14 in the GU-like group was significantly higher than that in the GS-like group while the expression level of Endothelial in the GU-like group was significantly lower than that in the GS-like group.

## References

[B1] Arun G. Diermeier S. Akerman M. Chang K. C. Wilkinson J. E. Hearn S. (2016). Differentiation of mammary tumors and reduction in metastasis upon Malat1 lncRNA loss. *Genes Dev* 30 34–51. 10.1101/gad.270959.115 26701265 PMC4701977

[B2] Bao S. Zhao H. Yuan J. Fan D. Zhang Z. Su J. (2020). Computational identification of mutator-derived lncRNA signatures of genome instability for improving the clinical outcome of cancers: a case study in breast cancer. *Brief Bioinform* 21 1742–1755. 10.1093/bib/bbz118 31665214

[B3] Barata P. C. Rini B. I. (2017). Treatment of renal cell carcinoma: Current status and future directions. *CA Cancer J Clin* 67 507–524. 28961310 10.3322/caac.21411

[B4] Bartkova J. Horejsí Z. Koed K. Krämer A. Tort F. Zieger K. (2005). DNA damage response as a candidate anti-cancer barrier in early human tumorigenesis. *Nature* 434 864–870. 10.1038/nature03482 15829956

[B5] Bruni D. Angell H. K. Galon J. (2020). The immune contexture and Immunoscore in cancer prognosis and therapeutic efficacy. *Nat Rev Cancer* 20 662–680. 10.1038/s41568-020-0285-7 32753728

[B6] Chen B. Khodadoust M. S. Liu C. L. Newman A. M. Alizadeh A. A. (2018). Profiling Tumor Infiltrating Immune Cells with CIBERSORT. *Methods Mol Biol* 1711 243–259. 10.1007/978-1-4939-7493-1_12 29344893 PMC5895181

[B7] Chu J. Li H. Xing Y. Jia J. Sheng J. Yang L. (2019). LncRNA MNX1-AS1 promotes progression of esophageal squamous cell carcinoma by regulating miR-34a/SIRT1 axis. *Biomed Pharmacother* 116 109029. 10.1016/j.biopha.2019.109029 31170665

[B8] Dyck L. Mills K. H. G. (2017). Immune checkpoints and their inhibition in cancer and infectious diseases. *Eur J Immunol* 47 765–779. 10.1002/eji.201646875 28393361

[B9] Garcia J. A. Rini B. I. (2007). Recent progress in the management of advanced renal cell carcinoma. *CA Cancer J Clin* 57 112–125. 10.3322/canjclin.57.2.112 17392388

[B10] Goodman A. M. Kato S. Bazhenova L. Patel S. P. Frampton G. M. Miller V. (2017). Tumor Mutational Burden as an Independent Predictor of Response to Immunotherapy in Diverse Cancers. *Mol Cancer Ther* 16 2598–2608. 10.1158/1535-7163.MCT-17-0386 28835386 PMC5670009

[B11] Gorgoulis V. G. Vassiliou L. V. Karakaidos P. Zacharatos P. Kotsinas A. Liloglou T. (2005). Activation of the DNA damage checkpoint and genomic instability in human precancerous lesions. *Nature* 434 907–913. 10.1038/nature03485 15829965

[B12] Gupta R. A. Shah N. Wang K. C. Kim J. Horlings H. M. Wong D. J. (2010). Long non-coding RNA HOTAIR reprograms chromatin state to promote cancer metastasis. *Nature* 464 1071–1076. 10.1038/nature08975 20393566 PMC3049919

[B13] Gulati S. Vaishampayan U. (2020). Current State of Systemic Therapies for Advanced Renal Cell Carcinoma. *Curr Oncol Rep* 22 26. 10.1007/s11912-020-0892-1 32048058

[B14] Habermann J. K. Doering J. Hautaniemi S. Roblick U. J. Bündgen N. K. Nicorici D. (2009). The gene expression signature of genomic instability in breast cancer is an independent predictor of clinical outcome. *Int J Cancer* 124 1552–1564. 10.1002/ijc.24017 19101988 PMC2707256

[B15] Herrem C. J. Tatsumi T. Olson K. S. Shirai K. Finke J. H. Bukowski R. M. (2005). Expression of EphA2 is prognostic of disease-free interval and overall survival in surgically treated patients with renal cell carcinoma. *Clin Cancer Res* 11 226–231. 15671550

[B16] Johnson D. E. O’Keefe R. A. Grandis J. R. (2018). Targeting the IL-6/JAK/STAT3 signalling axis in cancer. *Nat Rev Clin Oncol* 15 234–248. 10.1038/nrclinonc.2018.8 29405201 PMC5858971

[B17] Kumari N. Dwarakanath B. S. Das A. Bhatt A. N. (2016). Role of interleukin-6 in cancer progression and therapeutic resistance. *Tumour Biol* 37 11553–11572. 10.1007/s13277-016-5098-7 27260630

[B18] Li F. Chen Q. Xue H. Zhang L. Wang K. Shen F. (2020). LncRNA MNX1-AS1 promotes progression of intrahepatic cholangiocarcinoma through the MNX1/Hippo axis. *Cell Death Dis* 11 894. 10.1038/s41419-020-03029-0 33093444 PMC7581777

[B19] Liu H. Han L. Liu Z. Gao N. (2019). Long noncoding RNA MNX1-AS1 contributes to lung cancer progression through the miR-527/BRF2 pathway. *J Cell Physiol* 234 13843–13850. 10.1002/jcp.28064 30618167 PMC13483036

[B20] Li J. Li Q. Li D. Shen Z. Zhang K. Bi Z. (2020). Long Non-Coding RNA MNX1-AS1 Promotes Progression of Triple Negative Breast Cancer by Enhancing Phosphorylation of Stat3. *Front Oncol* 10:1108. 10.3389/fonc.2020.01108 32754442 PMC7366902

[B21] Lin Y. W. Lee L. M. Lee W. J. Chu C. Y. Tan P. Yang Y. C. (2016). Melatonin inhibits MMP-9 transactivation and renal cell carcinoma metastasis by suppressing Akt-MAPKs pathway and NF-κB DNA-binding activity. *J Pineal Res* 60 277–290. 10.1111/jpi.12308 26732239

[B22] Ljungberg B. Bensalah K. Canfield S. Dabestani S. Hofmann F. Hora M. (2015). EAU guidelines on renal cell carcinoma: 2014 update. *Eur Urol* 67 913–924. 25616710 10.1016/j.eururo.2015.01.005

[B23] Mirgayazova R. Khadiullina R. Mingaleeva R. Chasov V. Gomzikova M. Garanina E. (2019). Novel Isatin-based activator of p53 transcriptional functions in tumor cells. *Mol Biol Res Commun* 8 119–128. 31998813 10.22099/mbrc.2019.34179.1419 PMC6802691

[B24] Nagasaki T. Hara M. Nakanishi H. Takahashi H. Sato M. Takeyama H. (2014). Interleukin-6 released by colon cancer-associated fibroblasts is critical for tumour angiogenesis: anti-interleukin-6 receptor antibody suppressed angiogenesis and inhibited tumour-stroma interaction. *Br J Cancer* 110 469–478. 10.1038/bjc.2013.748 24346288 PMC3899773

[B25] Negrini S. Gorgoulis V. G. Halazonetis T. D. (2010). Genomic instability–an evolving hallmark of cancer. *Nat Rev Mol Cell Biol* 11 220–228. 10.1038/nrm2858 20177397

[B26] O’Donnell J. S. Teng M. W. L. Smyth M. J. (2019). Cancer immunoediting and resistance to T cell-based immunotherapy. *Nat Rev Clin Oncol* 16 151–167. 10.1038/s41571-018-0142-8 30523282

[B27] Ottini L. Falchetti M. Lupi R. Rizzolo P. Agnese V. Colucci G. (2006). Patterns of genomic instability in gastric cancer: clinical implications and perspectives. *Ann Oncol* 17(Suppl. 7), vii97–vii102. 16760303 10.1093/annonc/mdl960

[B28] Pripp A. H. (2018). [Pearson’s or Spearman’s correlation coefficients]. *Tidsskr Nor Laegeforen* 138 10.4045/tidsskr.18.0042 29737766

[B29] Schreiber R. D. Old L. J. Smyth M. J. (2011). Cancer immunoediting: integrating immunity’s roles in cancer suppression and promotion. *Science* 331 1565–1570. 10.1126/science.1203486 21436444

[B30] Shuai Y. Ma Z. Liu W. Yu T. Yan C. Jiang H. (2020). TEAD4 modulated LncRNA MNX1-AS1 contributes to gastric cancer progression partly through suppressing BTG2 and activating BCL2. *Mol Cancer* 19 6. 10.1186/s12943-019-1104-1 31924214 PMC6953272

[B31] Walter M. Liang S. Ghosh S. Hornsby P. J. Li R. (2009). Interleukin 6 secreted from adipose stromal cells promotes migration and invasion of breast cancer cells. *Oncogene* 28 2745–2755. 10.1038/onc.2009.130 19483720 PMC2806057

[B32] Wei X. Choudhury Y. Lim W. K. Anema J. Kahnoski R. J. Lane B. (2017). Recognizing the Continuous Nature of Expression Heterogeneity and Clinical Outcomes in Clear Cell Renal Cell Carcinoma. *Sci Rep* 7 7342. 10.1038/s41598-017-07191-y PMC5544702 28779136

[B33] Yu H. Pardoll D. Jove R. (2009). STATs in cancer inflammation and immunity: a leading role for STAT3. *Nat Rev Cancer* 9 798–809. 10.1038/nrc2734 19851315 PMC4856025

[B34] Yu H. Jove R. (2004). The STATs of cancer–new molecular targets come of age. *Nat Rev Cancer* 4 97–105. 10.1038/nrc1275 14964307

[B35] Yoshihara K. Shahmoradgoli M. Martínez E. Vegesna R. Kim H. Torres-Garcia W. (2013). Inferring tumour purity and stromal and immune cell admixture from expression data. *Nat Commun* 4 2612. 10.1038/ncomms3612 24113773 PMC3826632

[B36] Zeng J. H. Lu W. Liang L. Chen G. Lan H.-H. Lang X.-Y. (2019). Prognosis of clear cell renal cell carcinoma (ccRCC) based on a six-lncRNA-based risk score: an investigation based on RNA-sequencing data. *J Transl Med* 17 281. 10.1186/s12967-019-2032-y 31443717 PMC6708203

