# Supplementary material for: Genomic Instability Promotes the Progression of Clear Cell Renal Cell Carcinoma Through Influencing the Immune Microenvironment
Source: Front Genet. 2021 Oct 12;12:706661. doi: 10.3389/fgene.2021.706661 (PMC8546190; doi:10.3389/fgene.2021.706661)
Supplement: Supplementary file 1 [file Table_1.doc]

Supplementary Table 1. The group information separated by cumulative number of somatic mutations

| **id** | **Type** |
| --- | --- |
| TCGA-DV-5576-01A | LM |
| TCGA-B8-A8YJ-01A | LM |
| TCGA-B0-5083-01A | LM |
| TCGA-AK-3443-01A | LM |
| TCGA-A3-A8OX-01A | LM |
| TCGA-DV-5569-01A | LM |
| TCGA-B0-5117-01A | LM |
| TCGA-DV-5574-01A | LM |
| TCGA-BP-4760-01A | LM |
| TCGA-B8-5546-01A | LM |
| TCGA-DV-A4VZ-01A | LM |
| TCGA-CW-6097-01A | LM |
| TCGA-AK-3453-01A | LM |
| TCGA-DV-5567-01A | LM |
| TCGA-B0-4700-01A | LM |
| TCGA-BP-4177-01A | LM |
| TCGA-AK-3427-01A | LM |
| TCGA-A3-3374-01A | LM |
| TCGA-DV-5568-01A | LM |
| TCGA-BP-4975-01A | LM |
| TCGA-CJ-5681-01A | LM |
| TCGA-B8-5165-01A | LM |
| TCGA-B2-5636-01A | LM |
| TCGA-A3-A8OW-01A | LM |
| TCGA-B8-5545-01A | LM |
| TCGA-AK-3440-01A | LM |
| TCGA-AS-3777-01A | LM |
| TCGA-AK-3447-01A | LM |
| TCGA-BP-4795-01A | LM |
| TCGA-DV-5575-01A | LM |
| TCGA-B0-5081-01A | LM |
| TCGA-B8-5552-01B | LM |
| TCGA-CW-5583-01A | LM |
| TCGA-BP-5194-01A | LM |
| TCGA-BP-4987-01A | LM |
| TCGA-B8-A54K-01A | LM |
| TCGA-BP-5007-01A | LM |
| TCGA-BP-4970-01A | LM |
| TCGA-BP-4988-01A | LM |
| TCGA-BP-4974-01A | LM |
| TCGA-AK-3465-01A | LM |
| TCGA-6D-AA2E-01A | LM |
| TCGA-BP-5184-01A | LM |
| TCGA-CZ-5454-01A | LM |
| TCGA-DV-5573-01A | LM |
| TCGA-BP-4973-01A | LM |
| TCGA-B0-5400-01A | LM |
| TCGA-B8-A54F-01A | LM |
| TCGA-CJ-4908-01A | LM |
| TCGA-B0-5711-01A | LM |
| TCGA-CJ-4899-01A | LM |
| TCGA-CZ-5452-01A | LM |
| TCGA-BP-4962-01A | LM |
| TCGA-CZ-5458-01A | LM |
| TCGA-BP-5186-01A | LM |
| TCGA-B0-5100-01A | LM |
| TCGA-A3-3380-01A | LM |
| TCGA-BP-5008-01A | LM |
| TCGA-BP-5001-01A | LM |
| TCGA-T7-A92I-01A | LM |
| TCGA-B8-4148-01A | LM |
| TCGA-B4-5834-01A | LM |
| TCGA-BP-4998-01A | LM |
| TCGA-CW-5591-01A | LM |
| TCGA-BP-4982-01A | LM |
| TCGA-B0-5693-01A | LM |
| TCGA-B8-5551-01A | LM |
| TCGA-CJ-4904-01A | LM |
| TCGA-BP-4986-01A | LM |
| TCGA-BP-4961-01A | LM |
| TCGA-B0-5399-01A | LM |
| TCGA-BP-4992-01A | LM |
| TCGA-BP-5174-01A | LM |
| TCGA-EU-5904-01A | LM |
| TCGA-A3-3365-01A | LM |
| TCGA-BP-5202-01A | LM |
| TCGA-B2-4101-01A | LM |
| TCGA-BP-5006-01A | LM |
| TCGA-CZ-5989-01A | LM |
| TCGA-B0-5113-01A | LM |
| TCGA-CZ-4863-01A | LM |
| TCGA-B8-4146-01B | LM |
| TCGA-B0-5707-01A | LM |
| TCGA-A3-3370-01A | LM |
| TCGA-B0-5085-01A | HM |
| TCGA-B0-5099-01A | HM |
| TCGA-A3-3378-01A | HM |
| TCGA-BP-5182-01A | HM |
| TCGA-A3-3387-01A | HM |
| TCGA-B0-5709-01A | HM |
| TCGA-BP-4967-01A | HM |
| TCGA-B0-5700-01A | HM |
| TCGA-CJ-4918-01A | HM |
| TCGA-CJ-4869-01A | HM |
| TCGA-B0-5698-01A | HM |
| TCGA-B4-5836-01A | HM |
| TCGA-BP-4782-01A | HM |
| TCGA-CZ-5457-01A | HM |
| TCGA-CJ-5682-01A | HM |
| TCGA-A3-3319-01A | HM |
| TCGA-B0-5692-01A | HM |
| TCGA-BP-5173-01A | HM |
| TCGA-B0-5703-01A | HM |
| TCGA-CZ-5451-01A | HM |
| TCGA-B2-4102-01A | HM |
| TCGA-B8-5164-01A | HM |
| TCGA-B0-5095-01A | HM |
| TCGA-BP-5199-01A | HM |
| TCGA-CJ-6033-01A | HM |
| TCGA-BP-5198-01A | HM |
| TCGA-BP-4985-01A | HM |
| TCGA-CZ-5453-01A | HM |
| TCGA-BP-5185-01A | HM |
| TCGA-A3-A8OU-01A | HM |
| TCGA-B8-A54H-01A | HM |
| TCGA-B0-5705-01A | HM |
| TCGA-G6-A8L8-01A | HM |
| TCGA-A3-3308-01A | HM |
| TCGA-B0-5094-01A | HM |
| TCGA-A3-3317-01A | HM |
| TCGA-CJ-6027-01A | HM |
| TCGA-A3-3320-01A | HM |
| TCGA-B0-5106-01A | HM |
| TCGA-B0-4827-01A | HM |
| TCGA-CJ-4912-01A | HM |
| TCGA-CZ-4865-01A | HM |
| TCGA-CZ-4866-01A | HM |
| TCGA-G6-A8L7-01A | HM |
| TCGA-CZ-4864-01A | HM |
| TCGA-B8-4621-01A | HM |
| TCGA-B0-5096-01A | HM |
| TCGA-A3-A6NI-01A | HM |
| TCGA-B0-5119-01A | HM |
| TCGA-A3-3346-01A | HM |
| TCGA-BP-4963-01A | HM |
| TCGA-CW-5580-01A | HM |
| TCGA-B0-5075-01A | HM |
| TCGA-DV-A4VX-01A | HM |
| TCGA-MM-A84U-01A | HM |
| TCGA-AK-3444-01A | HM |
| TCGA-CJ-5679-01A | HM |
| TCGA-A3-3313-01A | HM |
| TCGA-BP-4976-01A | HM |
| TCGA-A3-3357-01A | HM |
| TCGA-CZ-5459-01A | HM |
| TCGA-CJ-5672-01A | HM |
| TCGA-3Z-A93Z-01A | HM |
| TCGA-B0-5713-01A | HM |
| TCGA-B8-5550-01A | HM |
| TCGA-CZ-4859-01A | HM |
| TCGA-A3-3382-01A | HM |
| TCGA-B0-5701-01A | HM |
| TCGA-A3-A6NN-01A | HM |
| TCGA-B0-5712-01A | HM |
| TCGA-CJ-6030-01A | HM |
| TCGA-MM-A564-01A | HM |
| TCGA-CZ-5465-01A | HM |
| TCGA-GK-A6C7-01A | HM |
| TCGA-B0-4823-01A | HM |
| TCGA-CW-6093-01A | HM |
| TCGA-CZ-4853-01A | HM |
| TCGA-CW-6090-01A | HM |
| TCGA-BP-5176-01A | HM |
| TCGA-CZ-5468-01A | HM |
| TCGA-BP-5168-01A | HM |
| TCGA-CJ-4920-01A | HM |
| TCGA-A3-A8OV-01A | HM |
| TCGA-B0-5098-01A | HM |
