# Supplementary material for: Genomic Instability Promotes the Progression of Clear Cell Renal Cell Carcinoma Through Influencing the Immune Microenvironment
Source: Front Genet. 2021 Oct 12;12:706661. doi: 10.3389/fgene.2021.706661 (PMC8546190; doi:10.3389/fgene.2021.706661)
Supplement: Supplementary file 2 [file Table_2.doc]

Supplementary Table 2. Differentially expressed genomic unstability-derived lncRNAs in ccRCC

| **lncRNA** | **conMean** | **treatMean** | **logFC** | **pValue** | **fdr** |
| --- | --- | --- | --- | --- | --- |
| AC010501.1 | 0.554787788 | 0.014874437 | -5.221029089 | 0.000290925 | 0.008329176 |
| KRT7-AS | 0.880505439 | 0.203615549 | -2.112484176 | 0.001015112 | 0.018479591 |
| IL21-AS1 | 0.029623805 | 0.121906719 | 2.0409488 | 0.003935906 | 0.043800435 |
| LINC01975 | 0.144297588 | 0.01402563 | -3.362909707 | 1.19E-05 | 0.001021406 |
| LINC01257 | 0.069283838 | 0.008811513 | -2.975057209 | 0.018467444 | 0.104093599 |
| AC007849.1 | 0.110240859 | 0.019155495 | -2.524828836 | 0.000977166 | 0.018144575 |
| AC002384.1 | 0.132551431 | 0.775775735 | 2.549087406 | 0.011705828 | 0.081364896 |
| LINC00885 | 0.40979349 | 0.015013514 | -4.770563517 | 0.000194173 | 0.006642428 |
| AC087564.1 | 0.067184663 | 0.299687589 | 2.1572555 | 0.026268822 | 0.126251421 |
| AL136088.1 | 0.184060757 | 0.01205008 | -3.933067447 | 1.97E-06 | 0.000337041 |
| PART1 | 0.738033114 | 0.006689754 | -6.78558851 | 2.95E-05 | 0.001938275 |
| AC116312.1 | 0.654445956 | 0.044339375 | -3.883613736 | 0.000418728 | 0.010713821 |
| AC010329.1 | 0.031284017 | 0.001641468 | -4.252367084 | 1.16E-05 | 0.001021406 |
| AC129507.4 | 1.510201803 | 0.109424282 | -3.786736516 | 0.003399012 | 0.040311646 |
| AC108860.2 | 0.106905171 | 0.026187963 | -2.029355882 | 0.001424112 | 0.023371983 |
| AC015660.1 | 0.502718235 | 0.117271166 | -2.09990169 | 0.026580392 | 0.126832577 |
| LINC00856 | 0.018515688 | 0.003866661 | -2.259588129 | 0.004705202 | 0.048384667 |
| AL356740.3 | 0.210725354 | 0.032845236 | -2.681607883 | 1.22E-05 | 0.001021406 |
| AL356740.1 | 0.310723406 | 0.03827674 | -3.021091033 | 6.05E-07 | 0.000178608 |
| AL928921.1 | 0.668883751 | 0.050854176 | -3.717317334 | 0.034423242 | 0.149343557 |
| AP001046.1 | 0.035730248 | 0.004694061 | -2.928237495 | 3.11E-05 | 0.0020012 |
| AC068057.1 | 0.081442332 | 0.015326157 | -2.409782851 | 0.003439964 | 0.040500587 |
| IGF2-AS | 0.296198718 | 0.009015615 | -5.037995648 | 0.001909931 | 0.028466551 |
| AC015921.1 | 0.040330274 | 0.00805678 | -2.323587864 | 1.54E-06 | 0.000323072 |
| AC100801.1 | 0.124466866 | 0.019612528 | -2.665914296 | 0.031814486 | 0.142007103 |
| AL139280.1 | 0.288444565 | 0.065031121 | -2.149091881 | 0.033793957 | 0.147996582 |
| AL161668.3 | 0.297363268 | 0.034196536 | -3.12030437 | 4.75E-09 | 6.17E-06 |
| AL035661.1 | 7.959842675 | 0.095996622 | -6.373612465 | 2.75E-07 | 0.000116466 |
| LINC01561 | 0.075639526 | 0.01761871 | -2.102032057 | 0.048474803 | 0.183055053 |
| AC104984.4 | 0.720652149 | 0.085447358 | -3.076195257 | 0.001281454 | 0.021575571 |
| NCOA7-AS1 | 0.253556314 | 0.014112948 | -4.16721487 | 0.000190211 | 0.006642428 |
| AP001476.1 | 0.139139901 | 0.021301201 | -2.707529548 | 0.000862193 | 0.016529184 |
| AL121827.1 | 0.01101478 | 0.092074005 | 3.063353246 | 0.009716726 | 0.073429071 |
| AL356740.2 | 0.220387604 | 0.028484804 | -2.951778669 | 1.61E-05 | 0.001213432 |
| AC079466.1 | 1.872596929 | 14.84734824 | 2.987092984 | 0.000113823 | 0.004772491 |
| C15orf59-AS1 | 0.976263359 | 0.01598034 | -5.932900409 | 0.00787267 | 0.065012045 |
| AC090241.2 | 0.092618203 | 0.005971275 | -3.955184882 | 0.000157489 | 0.006020704 |
| AL121820.2 | 0.890361413 | 0.063530151 | -3.808877708 | 5.73E-05 | 0.003027647 |
| LINC01213 | 0.331162969 | 0.045951641 | -2.849353055 | 0.047572139 | 0.181225867 |
| LINC00284 | 0.793877937 | 0.069457323 | -3.514718493 | 0.001684708 | 0.026510694 |
| AL031710.1 | 4.225273626 | 0.584016789 | -2.854963023 | 0.007312251 | 0.062283514 |
| AC007342.4 | 0.298473832 | 0.055106788 | -2.437302516 | 0.002123763 | 0.030401624 |
| AC007342.5 | 0.249769454 | 0.045811145 | -2.446826533 | 0.006684862 | 0.05866488 |
| HOXB-AS3 | 2.098001416 | 0.457872858 | -2.195996699 | 0.011764847 | 0.081466337 |
| AC016813.1 | 0.053946839 | 0.01066579 | -2.338547533 | 0.002030335 | 0.029652009 |
| LINC01606 | 1.04268963 | 0.037044052 | -4.814924067 | 0.000102693 | 0.004509459 |
| LINC01644 | 0.087365821 | 0.021750017 | -2.006052448 | 0.027621821 | 0.129707024 |
| AC008060.1 | 0.067662415 | 0.765616053 | 3.500194497 | 0.022975883 | 0.11781224 |
| LINC01096 | 0.033542358 | 0.007031376 | -2.254105264 | 0.020662281 | 0.110288366 |
| SEMA3B-AS1 | 1.677481818 | 0.418259147 | -2.003828133 | 0.017677623 | 0.101490167 |
| AC104237.2 | 0.316123181 | 0.061183394 | -2.36927478 | 0.001276089 | 0.021541042 |
| AC093904.4 | 0.112997239 | 0.004300516 | -4.715633801 | 0.001914123 | 0.028466551 |
| LINC00322 | 0.09519887 | 0.022987997 | -2.050063697 | 0.017851966 | 0.101861218 |
| LINC00452 | 0.040807713 | 0.007673772 | -2.410834021 | 0.009437722 | 0.072006348 |
| AC103563.7 | 1.23301507 | 0.284576464 | -2.115302177 | 1.93E-06 | 0.000337041 |
| AC023421.1 | 0.235107386 | 0.031469104 | -2.901311863 | 1.61E-08 | 1.31E-05 |
| AL592528.1 | 0.016713958 | 0.091780423 | 2.457133064 | 0.002042016 | 0.029689181 |
| AL031123.1 | 2.174436917 | 0.272414555 | -2.99676616 | 0.000171432 | 0.006366481 |
| AL353746.1 | 0.021293982 | 0.005230256 | -2.025492244 | 0.04998872 | 0.186389381 |
| LINC01896 | 1.29499793 | 0.010239824 | -6.9826151 | 0.000247794 | 0.007498109 |
| AC022387.1 | 0.090985906 | 0.022454862 | -2.018615219 | 0.039176246 | 0.160877459 |
| LINC01543 | 0.145539008 | 0.019854142 | -2.873893933 | 0.035129685 | 0.151397758 |
| LINC02302 | 0.016175769 | 0.002358783 | -2.777719559 | 0.000365128 | 0.009568403 |
| LINC01055 | 0.31929781 | 0.037801577 | -3.078384334 | 0.011360025 | 0.080215987 |
| LINC00982 | 0.767813239 | 0.069817958 | -3.45908536 | 2.87E-07 | 0.000116466 |
| AL049555.1 | 0.828481125 | 0.124321491 | -2.736393115 | 1.21E-05 | 0.001021406 |
| AC005082.1 | 1.574092774 | 0.329482221 | -2.256248048 | 1.03E-05 | 0.00095226 |
| THRB-AS1 | 0.208137005 | 0.031327032 | -2.732053481 | 0.001045128 | 0.018920013 |
| GDNF-AS1 | 0.065730313 | 0.010812894 | -2.60380617 | 0.006340154 | 0.057105656 |
| AC079062.1 | 0.029472413 | 0.007349323 | -2.003681912 | 0.011392003 | 0.080215987 |
| LY86-AS1 | 0.043909084 | 0.008729735 | -2.330509621 | 0.000185631 | 0.006642428 |
| LINC01230 | 1.870470158 | 0.006975414 | -8.066906378 | 0.000493716 | 0.011667856 |
| MCF2L-AS1 | 1.044612502 | 0.169011656 | -2.62777322 | 1.15E-08 | 1.07E-05 |
| AP000844.2 | 0.161920655 | 0.84339535 | 2.380922034 | 0.035391376 | 0.152222736 |
| CHL1-AS2 | 0.270624966 | 0.026820974 | -3.334861395 | 0.000108409 | 0.004665875 |
| LINC00645 | 0.700085034 | 0.167892545 | -2.059991998 | 0.002241131 | 0.031255598 |
| BX322234.2 | 0.052036869 | 0.255844596 | 2.297661697 | 0.010536785 | 0.077334847 |
| GATA3-AS1 | 0.084773071 | 0.01028204 | -3.043479449 | 0.000781676 | 0.015652403 |
| LINC01544 | 0.03042081 | 0.002699445 | -3.494324081 | 0.00030247 | 0.008509751 |
| LINC02268 | 0.034820484 | 0.151073309 | 2.117240644 | 0.004467049 | 0.046900404 |
| ZNF350-AS1 | 2.594613279 | 0.372113693 | -2.80170414 | 0.000181725 | 0.006621016 |
| LINC01234 | 0.192440462 | 0.891966052 | 2.212576631 | 0.00117505 | 0.020408094 |
| LINC01655 | 0.034900422 | 0.203655451 | 2.54481404 | 0.012723474 | 0.085574998 |
| AC010255.1 | 0.090332819 | 0.020134849 | -2.165555594 | 0.037632623 | 0.157586609 |
| AC061975.7 | 0.309412802 | 0.006187494 | -5.644033932 | 0.000154744 | 0.005950784 |
| AL137793.1 | 0.062796855 | 0.013665343 | -2.200170685 | 0.002525059 | 0.033695309 |
| AC104237.3 | 0.637596609 | 0.079813953 | -2.997931075 | 0.000189161 | 0.006642428 |
| AC025431.1 | 0.1415252 | 0.021891982 | -2.692584499 | 5.30E-06 | 0.000632201 |
| AC243585.1 | 0.250163758 | 0.017149163 | -3.866662725 | 0.000587654 | 0.013311635 |
| LINC01187 | 9.945564339 | 0.023550597 | -8.722145794 | 8.74E-05 | 0.004118563 |
| AP000757.1 | 2.437194258 | 0.513291328 | -2.247371455 | 5.19E-06 | 0.000632201 |
| AC114811.2 | 0.18009335 | 0.027916656 | -2.689546844 | 4.98E-05 | 0.002698633 |
| AL020994.2 | 0.108374452 | 0.007276657 | -3.896605164 | 0.001574024 | 0.025134102 |
| AC148477.4 | 2.393869577 | 0.082192279 | -4.864197861 | 0.000241108 | 0.007468408 |
| AC079296.1 | 0.021139221 | 0.004719021 | -2.163362731 | 8.68E-05 | 0.004118563 |
| AC023421.2 | 0.352042126 | 0.055108427 | -2.675403232 | 0.032678018 | 0.144570753 |
