# Supplementary material for: Genomic Instability Promotes the Progression of Clear Cell Renal Cell Carcinoma Through Influencing the Immune Microenvironment
Source: Front Genet. 2021 Oct 12;12:706661. doi: 10.3389/fgene.2021.706661 (PMC8546190; doi:10.3389/fgene.2021.706661)
Supplement: Supplementary file 3 [file Table_3.doc]

Supplementary Table 3.The group information clustered by the expression of 148 genomic unstability-derived lncRNAs

| **Sample id** | **Group** |
| --- | --- |
| TCGA-CJ-4920-01A-01R-1426-07 | GU-like |
| TCGA-BP-5177-01A-01R-1426-07 | GS-like |
| TCGA-B0-4842-01A-02R-1420-07 | GU-like |
| TCGA-BP-4343-01A-02R-1289-07 | GS-like |
| TCGA-MW-A4EC-01A-11R-A266-07 | GS-like |
| TCGA-B8-4143-01A-01R-1188-07 | GU-like |
| TCGA-CJ-5675-01A-11R-1541-07 | GU-like |
| TCGA-BP-4760-01A-02R-1420-07 | GS-like |
| TCGA-B0-5113-01A-01R-1420-07 | GS-like |
| TCGA-BP-4340-01A-01R-1289-07 | GS-like |
| TCGA-B8-A7U6-01A-12R-A37O-07 | GS-like |
| TCGA-B8-5551-01A-01R-1541-07 | GU-like |
| TCGA-B0-5400-01A-01R-1503-07 | GS-like |
| TCGA-BP-4994-01A-01R-1334-07 | GS-like |
| TCGA-A3-3349-01A-01R-1188-07 | GU-like |
| TCGA-AK-3455-01A-01R-0864-07 | GU-like |
| TCGA-CW-5585-01A-01R-1541-07 | GS-like |
| TCGA-BP-4981-01A-01R-1334-07 | GU-like |
| TCGA-BP-4337-01A-01R-1289-07 | GU-like |
| TCGA-CJ-5686-01A-11R-1672-07 | GU-like |
| TCGA-A3-A8OV-01A-11R-A37O-07 | GU-like |
| TCGA-AK-3447-01A-01R-1766-07 | GS-like |
| TCGA-B0-5702-01A-11R-1541-07 | GS-like |
| TCGA-CJ-4918-01A-01R-1426-07 | GU-like |
| TCGA-B0-4848-01A-01R-1277-07 | GU-like |
| TCGA-BP-5194-01A-02R-1426-07 | GS-like |
| TCGA-B2-5635-01B-04R-A277-07 | GS-like |
| TCGA-B0-5096-01A-01R-1420-07 | GU-like |
| TCGA-CZ-5986-01A-11R-1672-07 | GS-like |
| TCGA-A3-3380-01A-01R-0864-07 | GS-like |
| TCGA-BP-4329-01A-02R-1289-07 | GS-like |
| TCGA-B0-4712-01A-01R-1503-07 | GU-like |
| TCGA-B0-4691-01A-01R-1277-07 | GU-like |
| TCGA-CZ-5982-01A-11R-1672-07 | GS-like |
| TCGA-BP-4962-01A-01R-1334-07 | GU-like |
| TCGA-CZ-5466-01A-01R-1503-07 | GU-like |
| TCGA-AK-3450-01A-02R-1277-07 | GS-like |
| TCGA-CJ-4876-01A-01R-1305-07 | GU-like |
| TCGA-B0-4834-01A-01R-1305-07 | GS-like |
| TCGA-B0-5713-01A-11R-1672-07 | GS-like |
| TCGA-BP-5009-01A-01R-1334-07 | GU-like |
| TCGA-CJ-4900-01A-01R-1334-07 | GU-like |
| TCGA-BP-5186-01A-01R-1426-07 | GS-like |
| TCGA-A3-3370-01A-02R-1420-07 | GS-like |
| TCGA-B0-4810-01A-01R-1503-07 | GU-like |
| TCGA-CJ-4916-01A-01R-1426-07 | GU-like |
| TCGA-B0-4846-01A-01R-1277-07 | GU-like |
| TCGA-CJ-4638-01A-02R-1325-07 | GU-like |
| TCGA-BP-5184-01A-01R-1426-07 | GS-like |
| TCGA-B0-5116-01A-02R-1420-07 | GU-like |
| TCGA-CZ-4857-01A-01R-1305-07 | GU-like |
| TCGA-B0-4718-01A-01R-1277-07 | GU-like |
| TCGA-B0-5696-01A-11R-1541-07 | GU-like |
| TCGA-BP-4326-01A-01R-1289-07 | GU-like |
| TCGA-CZ-4856-01A-02R-1426-07 | GS-like |
| TCGA-B2-5635-01A-01R-1541-07 | GU-like |
| TCGA-BP-4972-01A-01R-1334-07 | GS-like |
| TCGA-BP-5169-01A-01R-1426-07 | GU-like |
| TCGA-B2-5633-01A-01R-A277-07 | GU-like |
| TCGA-B0-5693-01A-11R-1541-07 | GS-like |
| TCGA-BP-4969-01A-01R-1334-07 | GU-like |
| TCGA-BP-4165-01A-02R-1289-07 | GS-like |
| TCGA-CJ-4870-01A-01R-1305-07 | GS-like |
| TCGA-B8-5552-01B-11R-1672-07 | GU-like |
| TCGA-BP-4164-01A-02R-1325-07 | GS-like |
| TCGA-BP-4987-01A-01R-1334-07 | GU-like |
| TCGA-BP-4769-01A-01R-1289-07 | GS-like |
| TCGA-BP-4161-01A-02R-1325-07 | GU-like |
| TCGA-BP-5010-01A-02R-1420-07 | GS-like |
| TCGA-B2-3923-01B-10R-A277-07 | GS-like |
| TCGA-AK-3445-01A-02R-1277-07 | GU-like |
| TCGA-B2-4102-01A-02R-1325-07 | GU-like |
| TCGA-B0-5083-01A-02R-1420-07 | GS-like |
| TCGA-B0-4697-01A-01R-1277-07 | GU-like |
| TCGA-B2-3924-01A-02R-1325-07 | GU-like |
| TCGA-DV-A4VZ-01A-11R-A266-07 | GS-like |
| TCGA-B0-4821-01A-01R-1503-07 | GU-like |
| TCGA-B0-4813-01A-01R-1277-07 | GS-like |
| TCGA-CZ-5459-01A-01R-1503-07 | GU-like |
| TCGA-B0-5108-01A-01R-1420-07 | GU-like |
| TCGA-CZ-4862-01A-01R-1305-07 | GS-like |
| TCGA-MM-A564-01A-11R-A266-07 | GS-like |
| TCGA-CJ-4881-01A-01R-1305-07 | GU-like |
| TCGA-B0-5077-01A-01R-1334-07 | GS-like |
| TCGA-B0-5092-01A-01R-1420-07 | GU-like |
| TCGA-EU-5905-01A-11R-1672-07 | GU-like |
| TCGA-BP-5168-01A-01R-1420-07 | GS-like |
| TCGA-B4-5832-01A-11R-1672-07 | GU-like |
| TCGA-B8-5163-01A-01R-1420-07 | GU-like |
| TCGA-DV-5575-01A-01R-1541-07 | GU-like |
| TCGA-CW-5580-01A-01R-1672-07 | GU-like |
| TCGA-B0-5692-01A-11R-1541-07 | GU-like |
| TCGA-CJ-4640-01A-02R-1325-07 | GS-like |
| TCGA-A3-3316-01A-01R-0864-07 | GU-like |
| TCGA-BP-4797-01A-01R-1305-07 | GU-like |
| TCGA-BP-5195-01A-02R-1426-07 | GU-like |
| TCGA-B0-5691-01A-11R-1541-07 | GS-like |
| TCGA-BP-5006-01A-01R-1334-07 | GS-like |
| TCGA-CZ-5469-01A-01R-1503-07 | GU-like |
| TCGA-BP-4999-01A-01R-1334-07 | GS-like |
| TCGA-EU-5904-01A-11R-1672-07 | GS-like |
| TCGA-B2-3924-01B-03R-A277-07 | GS-like |
| TCGA-B2-3924-01A-02R-A277-07 | GS-like |
| TCGA-BP-5189-01A-02R-1426-07 | GU-like |
| TCGA-B0-5812-01A-11R-1672-07 | GS-like |
| TCGA-A3-3385-01A-02R-1420-07 | GU-like |
| TCGA-BP-4787-01A-01R-1305-07 | GU-like |
| TCGA-BP-4325-01A-02R-1289-07 | GS-like |
| TCGA-CJ-5677-01A-11R-1541-07 | GU-like |
| TCGA-BP-4167-01A-02R-1325-07 | GU-like |
| TCGA-CW-6097-01A-11R-1672-07 | GU-like |
| TCGA-B8-5162-01A-01R-1420-07 | GS-like |
| TCGA-CJ-6031-01A-11R-1672-07 | GS-like |
| TCGA-CJ-4639-01A-02R-1325-07 | GS-like |
| TCGA-BP-4759-01A-01R-1289-07 | GS-like |
| TCGA-B8-4621-01A-01R-1503-07 | GU-like |
| TCGA-CZ-5452-01A-01R-1503-07 | GU-like |
| TCGA-B0-5699-01A-11R-1541-07 | GU-like |
| TCGA-CZ-5460-01A-01R-1503-07 | GU-like |
| TCGA-B0-4845-01A-01R-1277-07 | GS-like |
| TCGA-MM-A563-01A-11R-A266-07 | GU-like |
| TCGA-BP-4971-01A-01R-1334-07 | GS-like |
| TCGA-B0-5712-01A-11R-1672-07 | GU-like |
| TCGA-B0-4706-01A-01R-1503-07 | GU-like |
| TCGA-CZ-5461-01A-01R-1503-07 | GU-like |
| TCGA-6D-AA2E-01A-11R-A37O-07 | GU-like |
| TCGA-EU-5907-01A-11R-1672-07 | GU-like |
| TCGA-A3-3323-01A-02R-1325-07 | GU-like |
| TCGA-CJ-4895-01A-01R-1305-07 | GU-like |
| TCGA-CW-5591-01A-01R-1541-07 | GS-like |
| TCGA-BP-4799-01A-01R-1305-07 | GU-like |
| TCGA-B0-4847-01A-01R-1277-07 | GS-like |
| TCGA-CW-6087-01A-11R-1672-07 | GU-like |
| TCGA-BP-4173-01A-02R-1289-07 | GU-like |
| TCGA-B8-4619-01A-02R-1325-07 | GS-like |
| TCGA-BP-4176-01A-02R-1289-07 | GU-like |
| TCGA-BP-4768-01A-01R-1289-07 | GU-like |
| TCGA-B8-A54I-01A-21R-A33J-07 | GU-like |
| TCGA-BP-5192-01A-01R-1426-07 | GS-like |
| TCGA-BP-5181-01A-01R-1426-07 | GS-like |
| TCGA-EU-5906-01A-11R-1672-07 | GS-like |
| TCGA-B8-4620-01A-02R-1325-07 | GU-like |
| TCGA-BP-4959-01A-01R-1334-07 | GU-like |
| TCGA-CJ-4905-01A-02R-1426-07 | GS-like |
| TCGA-CJ-6032-01A-11R-1672-07 | GU-like |
| TCGA-B0-5703-01A-11R-1541-07 | GS-like |
| TCGA-B8-A54F-01A-11R-A266-07 | GS-like |
| TCGA-B0-5095-01A-01R-1420-07 | GU-like |
| TCGA-CZ-4861-01A-01R-1305-07 | GU-like |
| TCGA-BP-4964-01A-01R-1334-07 | GS-like |
| TCGA-B0-5402-01A-01R-1503-07 | GS-like |
| TCGA-CJ-4891-01A-01R-1305-07 | GU-like |
| TCGA-B0-4690-01A-01R-1277-07 | GU-like |
| TCGA-A3-3374-01A-02R-1325-07 | GS-like |
| TCGA-CZ-5470-01A-01R-1503-07 | GU-like |
| TCGA-CZ-5463-01A-01R-1503-07 | GU-like |
| TCGA-B8-4153-01B-11R-1672-07 | GS-like |
| TCGA-B0-4698-01A-01R-1503-07 | GU-like |
| TCGA-B0-5106-01A-01R-1420-07 | GU-like |
| TCGA-CJ-4892-01A-01R-1305-07 | GS-like |
| TCGA-CZ-4863-01A-01R-1503-07 | GS-like |
| TCGA-BP-4330-01A-01R-1289-07 | GS-like |
| TCGA-B0-4824-01A-01R-1277-07 | GS-like |
| TCGA-CJ-4871-01A-01R-1305-07 | GU-like |
| TCGA-B0-5690-01A-11R-1541-07 | GU-like |
| TCGA-B8-5549-01A-01R-1541-07 | GU-like |
| TCGA-DV-A4W0-01A-11R-A266-07 | GS-like |
| TCGA-CZ-5989-01A-11R-1672-07 | GU-like |
| TCGA-B0-5081-01A-01R-1334-07 | GU-like |
| TCGA-CZ-4866-01A-01R-1503-07 | GU-like |
| TCGA-BP-5182-01A-01R-1426-07 | GU-like |
| TCGA-CJ-6033-01A-11R-1672-07 | GU-like |
| TCGA-BP-4327-01A-01R-1289-07 | GU-like |
| TCGA-BP-5200-01A-01R-1426-07 | GS-like |
| TCGA-BP-4160-01A-02R-1289-07 | GU-like |
| TCGA-B0-5120-01A-01R-1420-07 | GS-like |
| TCGA-B0-5100-01A-01R-1420-07 | GS-like |
| TCGA-B0-4945-01A-01R-1420-07 | GU-like |
| TCGA-BP-4781-01A-01R-1305-07 | GU-like |
| TCGA-CJ-5684-01A-11R-1541-07 | GS-like |
| TCGA-BP-4346-01A-01R-1289-07 | GU-like |
| TCGA-AK-3426-01A-02R-1325-07 | GS-like |
| TCGA-CJ-5682-01A-11R-1541-07 | GU-like |
| TCGA-A3-A6NL-01A-11R-A33J-07 | GS-like |
| TCGA-B2-5633-01B-04R-A277-07 | GS-like |
| TCGA-CZ-5987-01A-11R-1672-07 | GU-like |
| TCGA-A3-3382-01A-02R-1325-07 | GU-like |
| TCGA-CJ-4636-01A-02R-1325-07 | GU-like |
| TCGA-CW-5583-01A-02R-1541-07 | GS-like |
| TCGA-B0-4849-01A-01R-1277-07 | GS-like |
| TCGA-B0-4852-01A-01R-1503-07 | GU-like |
| TCGA-BP-4995-01A-01R-1334-07 | GS-like |
| TCGA-BP-4798-01A-01R-1305-07 | GU-like |
| TCGA-B0-5700-01A-11R-1541-07 | GS-like |
| TCGA-AK-3456-01A-02R-1325-07 | GS-like |
| TCGA-CJ-4641-01A-02R-1325-07 | GU-like |
| TCGA-BP-4335-01A-01R-1289-07 | GS-like |
| TCGA-B8-4146-01B-11R-1672-07 | GU-like |
| TCGA-B0-4694-01A-01R-1277-07 | GU-like |
| TCGA-B0-4836-01A-01R-1305-07 | GU-like |
| TCGA-CJ-4635-01A-02R-1305-07 | GU-like |
| TCGA-BP-4975-01A-01R-1334-07 | GS-like |
| TCGA-B8-A54E-01A-11R-A266-07 | GS-like |
| TCGA-A3-3376-01A-02R-1420-07 | GS-like |
| TCGA-BP-4758-01A-01R-1289-07 | GU-like |
| TCGA-B0-5121-01A-02R-1420-07 | GU-like |
| TCGA-BP-5180-01A-01R-1426-07 | GU-like |
| TCGA-DV-5566-01A-01R-1541-07 | GU-like |
| TCGA-BP-4784-01A-01R-1305-07 | GS-like |
| TCGA-A3-3331-01A-02R-1325-07 | GS-like |
| TCGA-CZ-5455-01A-01R-1503-07 | GS-like |
| TCGA-A3-3383-01A-02R-1325-07 | GS-like |
| TCGA-CJ-4875-01A-01R-1305-07 | GU-like |
| TCGA-B0-4811-01A-01R-1503-07 | GU-like |
| TCGA-BP-4341-01A-01R-1289-07 | GS-like |
| TCGA-B0-5099-01A-01R-1420-07 | GU-like |
| TCGA-B0-4839-01A-01R-1305-07 | GU-like |
| TCGA-CJ-5678-01A-11R-1541-07 | GU-like |
| TCGA-DV-A4VX-01A-11R-A266-07 | GU-like |
| TCGA-CJ-6030-01A-11R-1672-07 | GU-like |
| TCGA-B0-4818-01A-01R-1503-07 | GS-like |
| TCGA-B0-5706-01A-11R-1541-07 | GU-like |
| TCGA-BP-4973-01A-01R-1334-07 | GU-like |
| TCGA-B8-5165-01A-01R-1420-07 | GS-like |
| TCGA-CJ-4901-01A-01R-1426-07 | GU-like |
| TCGA-B0-5110-01A-01R-1420-07 | GS-like |
| TCGA-B0-4838-01A-01R-1305-07 | GU-like |
| TCGA-BP-4968-01A-01R-1334-07 | GS-like |
| TCGA-A3-A8OX-01A-11R-A37O-07 | GS-like |
| TCGA-A3-A6NN-01A-12R-A33J-07 | GU-like |
| TCGA-B0-4814-01A-01R-1277-07 | GS-like |
| TCGA-B0-4823-01A-02R-1420-07 | GU-like |
| TCGA-B0-5098-01A-01R-1420-07 | GU-like |
| TCGA-B0-5711-01A-11R-1672-07 | GS-like |
| TCGA-A3-3322-01A-02R-1325-07 | GU-like |
| TCGA-B8-4151-01A-01R-1188-07 | GU-like |
| TCGA-BP-4982-01A-01R-1334-07 | GS-like |
| TCGA-B0-4713-01A-01R-1277-07 | GU-like |
| TCGA-CJ-4902-01A-01R-1426-07 | GS-like |
| TCGA-B0-5399-01A-01R-1503-07 | GU-like |
| TCGA-B0-4707-01A-01R-1277-07 | GU-like |
| TCGA-CZ-4865-01A-02R-1503-07 | GU-like |
| TCGA-BP-4344-01A-01R-1289-07 | GS-like |
| TCGA-DV-5565-01A-01R-1541-07 | GU-like |
| TCGA-AK-3436-01A-02R-1325-07 | GU-like |
| TCGA-A3-A6NJ-01A-12R-A33J-07 | GS-like |
| TCGA-BP-5191-01A-01R-1426-07 | GU-like |
| TCGA-B8-4154-01A-01R-1188-07 | GS-like |
| TCGA-B0-4688-01A-01R-1277-07 | GU-like |
| TCGA-BP-4986-01A-01R-1334-07 | GU-like |
| TCGA-B0-5075-01A-01R-1334-07 | GS-like |
| TCGA-BP-4775-01A-01R-1289-07 | GS-like |
| TCGA-CJ-4888-01A-01R-1305-07 | GU-like |
| TCGA-AK-3431-01A-02R-1277-07 | GU-like |
| TCGA-BP-4338-01A-01R-1289-07 | GU-like |
| TCGA-B0-4701-01A-01R-1277-07 | GS-like |
| TCGA-A3-3325-01A-01R-0864-07 | GU-like |
| TCGA-CW-6090-01A-11R-1672-07 | GU-like |
| TCGA-BP-4177-01A-02R-1420-07 | GS-like |
| TCGA-BP-4170-01A-02R-1289-07 | GS-like |
| TCGA-A3-3357-01A-02R-1420-07 | GU-like |
| TCGA-BP-4960-01A-01R-1334-07 | GU-like |
| TCGA-AK-3440-01A-02R-1277-07 | GS-like |
| TCGA-A3-3365-01A-01R-0864-07 | GS-like |
| TCGA-DV-5569-01A-01R-1541-07 | GS-like |
| TCGA-BP-5170-01A-01R-1426-07 | GS-like |
| TCGA-CJ-6027-01A-11R-1672-07 | GU-like |
| TCGA-BP-4983-01A-01R-1334-07 | GU-like |
| TCGA-B4-5378-01A-01R-1503-07 | GS-like |
| TCGA-A3-3317-01A-02R-1325-07 | GU-like |
| TCGA-BP-4771-01A-01R-1289-07 | GU-like |
| TCGA-A3-3352-01A-01R-0864-07 | GU-like |
| TCGA-B0-5084-01A-01R-1334-07 | GU-like |
| TCGA-BP-4159-01A-02R-1289-07 | GS-like |
| TCGA-B8-A54J-01A-11R-A33J-07 | GS-like |
| TCGA-CJ-4644-01A-02R-1325-07 | GU-like |
| TCGA-B4-5836-01A-11R-1672-07 | GU-like |
| TCGA-B0-5117-01A-01R-1420-07 | GS-like |
| TCGA-CJ-5680-01A-11R-1541-07 | GS-like |
| TCGA-DV-A4W0-05A-11R-A266-07 | GS-like |
| TCGA-CW-5589-01A-01R-1541-07 | GS-like |
| TCGA-BP-5000-01A-01R-1334-07 | GU-like |
| TCGA-A3-3346-01A-01R-1766-07 | GU-like |
| TCGA-CJ-5689-01A-11R-1541-07 | GU-like |
| TCGA-3Z-A93Z-01A-11R-A37O-07 | GS-like |
| TCGA-BP-4807-01A-01R-1305-07 | GU-like |
| TCGA-CJ-4908-01A-01R-1426-07 | GS-like |
| TCGA-BP-4352-01A-01R-1289-07 | GU-like |
| TCGA-A3-A8OU-01A-11R-A37O-07 | GU-like |
| TCGA-AK-3434-01A-02R-1277-07 | GU-like |
| TCGA-CJ-5683-01A-11R-1541-07 | GU-like |
| TCGA-A3-3326-01A-01R-0864-07 | GS-like |
| TCGA-CJ-5671-01A-11R-1541-07 | GU-like |
| TCGA-AK-3425-01A-02R-1277-07 | GU-like |
| TCGA-CZ-4858-01A-01R-1305-07 | GU-like |
| TCGA-B0-4841-01A-01R-1277-07 | GU-like |
| TCGA-CJ-4897-01A-03R-1426-07 | GS-like |
| TCGA-BP-4776-01A-01R-1289-07 | GS-like |
| TCGA-B4-5377-01A-01R-1503-07 | GS-like |
| TCGA-CZ-5468-01A-01R-1503-07 | GU-like |
| TCGA-CJ-4904-01A-02R-1426-07 | GS-like |
| TCGA-BP-4756-01A-01R-1289-07 | GS-like |
| TCGA-B0-5088-01A-01R-1334-07 | GU-like |
| TCGA-B0-4837-01A-01R-1305-07 | GU-like |
| TCGA-BP-5174-01A-01R-1426-07 | GU-like |
| TCGA-BP-4803-01A-01R-1305-07 | GU-like |
| TCGA-B0-5097-01A-01R-1420-07 | GU-like |
| TCGA-B0-5119-01A-02R-1420-07 | GS-like |
| TCGA-BP-4345-01A-01R-1289-07 | GS-like |
| TCGA-AK-3453-01A-02R-1277-07 | GU-like |
| TCGA-BP-4342-01A-01R-1289-07 | GS-like |
| TCGA-B0-4844-01A-01R-1277-07 | GU-like |
| TCGA-BP-4804-01A-02R-1305-07 | GS-like |
| TCGA-CW-6088-01A-11R-1672-07 | GU-like |
| TCGA-B0-5701-01A-11R-1541-07 | GU-like |
| TCGA-CJ-5681-01A-11R-1541-07 | GS-like |
| TCGA-B2-3923-01A-02R-A277-07 | GS-like |
| TCGA-A3-3319-01A-02R-1325-07 | GU-like |
| TCGA-BP-5007-01A-01R-1334-07 | GS-like |
| TCGA-B8-A54D-01A-21R-A266-07 | GU-like |
| TCGA-B0-4833-01A-01R-1305-07 | GS-like |
| TCGA-BP-4347-01A-01R-1289-07 | GS-like |
| TCGA-CW-5590-01A-01R-1541-07 | GU-like |
| TCGA-B0-4822-01A-01R-1277-07 | GU-like |
| TCGA-B0-4703-01A-01R-1277-07 | GU-like |
| TCGA-BP-4770-01A-01R-1503-07 | GU-like |
| TCGA-CJ-4637-01A-02R-1325-07 | GU-like |
| TCGA-CJ-4885-01A-01R-1305-07 | GS-like |
| TCGA-B8-5546-01A-01R-1541-07 | GS-like |
| TCGA-B8-5545-01A-01R-1672-07 | GU-like |
| TCGA-CJ-5676-01A-11R-1541-07 | GU-like |
| TCGA-B0-5707-01A-11R-1541-07 | GS-like |
| TCGA-BP-5190-01A-01R-1426-07 | GS-like |
| TCGA-B0-5709-01A-11R-1541-07 | GU-like |
| TCGA-B0-5109-01A-02R-1420-07 | GU-like |
| TCGA-B4-5843-01A-11R-1672-07 | GU-like |
| TCGA-B2-4098-01A-02R-1325-07 | GS-like |
| TCGA-CJ-4893-01A-01R-1305-07 | GU-like |
| TCGA-BP-4985-01A-01R-1334-07 | GU-like |
| TCGA-BP-5001-01A-01R-1334-07 | GU-like |
| TCGA-A3-3313-01A-02R-1325-07 | GU-like |
| TCGA-BP-4169-01A-02R-1289-07 | GU-like |
| TCGA-B8-5553-01A-01R-1541-07 | GU-like |
| TCGA-AK-3443-01A-02R-1325-07 | GS-like |
| TCGA-B0-4696-01A-01R-1277-07 | GU-like |
| TCGA-DV-5576-01A-01R-1541-07 | GS-like |
| TCGA-BP-4963-01A-01R-1334-07 | GS-like |
| TCGA-BP-4354-01A-02R-1289-07 | GU-like |
| TCGA-BP-4789-01A-01R-1305-07 | GS-like |
| TCGA-CZ-5462-01A-01R-1503-07 | GU-like |
| TCGA-B0-5705-01A-11R-1541-07 | GU-like |
| TCGA-B0-4710-01A-01R-1503-07 | GU-like |
| TCGA-BP-5004-01A-01R-1334-07 | GS-like |
| TCGA-A3-3387-01A-01R-1541-07 | GU-like |
| TCGA-BP-5175-01A-01R-1426-07 | GU-like |
| TCGA-BP-4331-01A-01R-1289-07 | GU-like |
| TCGA-BP-4174-01A-02R-1289-07 | GU-like |
| TCGA-CJ-6028-01A-11R-1672-07 | GU-like |
| TCGA-A3-3367-01A-02R-1420-07 | GU-like |
| TCGA-A3-3351-01A-02R-1325-07 | GS-like |
| TCGA-CJ-4890-01A-01R-1305-07 | GS-like |
| TCGA-CW-5581-01A-02R-1541-07 | GU-like |
| TCGA-B0-4815-01A-01R-1503-07 | GU-like |
| TCGA-B2-5635-01A-01R-A277-07 | GU-like |
| TCGA-AK-3454-01A-02R-1277-07 | GU-like |
| TCGA-BP-4782-01A-02R-1420-07 | GU-like |
| TCGA-CZ-5451-01A-01R-1503-07 | GS-like |
| TCGA-CJ-4878-01A-01R-1305-07 | GS-like |
| TCGA-CJ-5672-01A-11R-1541-07 | GU-like |
| TCGA-BP-4765-01A-01R-1289-07 | GU-like |
| TCGA-BP-4970-01A-01R-1334-07 | GS-like |
| TCGA-B0-4819-01A-01R-1277-07 | GU-like |
| TCGA-G6-A5PC-01A-11R-A33J-07 | GS-like |
| TCGA-BP-4992-01A-01R-1334-07 | GU-like |
| TCGA-CZ-4864-01A-01R-1503-07 | GS-like |
| TCGA-BP-5198-01A-01R-1426-07 | GU-like |
| TCGA-BP-4349-01A-01R-1289-07 | GU-like |
| TCGA-BP-4965-01A-01R-1334-07 | GS-like |
| TCGA-B4-5835-01A-11R-1672-07 | GU-like |
| TCGA-B0-5094-01A-01R-1420-07 | GU-like |
| TCGA-DV-5567-01A-01R-1541-07 | GS-like |
| TCGA-BP-4334-01A-01R-1289-07 | GS-like |
| TCGA-BP-5187-01A-01R-1426-07 | GU-like |
| TCGA-CZ-5988-01A-11R-1672-07 | GU-like |
| TCGA-CJ-4894-01A-01R-1305-07 | GU-like |
| TCGA-BP-4763-01A-01R-1289-07 | GS-like |
| TCGA-B0-4828-01A-01R-1277-07 | GU-like |
| TCGA-CJ-5679-01A-11R-1541-07 | GU-like |
| TCGA-CJ-4869-01A-02R-1426-07 | GU-like |
| TCGA-A3-3363-01A-01R-0864-07 | GS-like |
| TCGA-BP-4777-01A-01R-1289-07 | GU-like |
| TCGA-B0-4843-01A-01R-1277-07 | GU-like |
| TCGA-BP-4766-01A-01R-1289-07 | GU-like |
| TCGA-B8-A54H-01A-11R-A33J-07 | GS-like |
| TCGA-BP-4774-01A-01R-1289-07 | GS-like |
| TCGA-A3-3373-01A-02R-1420-07 | GU-like |
| TCGA-CZ-4860-01A-01R-1305-07 | GU-like |
| TCGA-AK-3451-01A-02R-1188-07 | GU-like |
| TCGA-B0-5104-01A-01R-1420-07 | GU-like |
| TCGA-BP-4351-01A-01R-1289-07 | GS-like |
| TCGA-B2-4099-01A-02R-1188-07 | GS-like |
| TCGA-B0-5694-01A-11R-1541-07 | GS-like |
| TCGA-CJ-4903-01A-01R-1426-07 | GU-like |
| TCGA-CZ-5457-01A-01R-1503-07 | GS-like |
| TCGA-BP-4158-01A-02R-1289-07 | GS-like |
| TCGA-A3-3320-01A-02R-1325-07 | GS-like |
| TCGA-DV-5568-01A-01R-1541-07 | GU-like |
| TCGA-CZ-5985-01A-11R-1672-07 | GU-like |
| TCGA-BP-4166-01A-02R-1289-07 | GU-like |
| TCGA-AS-3777-01A-01R-0864-07 | GS-like |
| TCGA-CZ-5456-01A-01R-1503-07 | GU-like |
| TCGA-BP-5183-01A-01R-1426-07 | GU-like |
| TCGA-CZ-4854-01A-01R-1305-07 | GU-like |
| TCGA-A3-3306-01A-01R-0864-07 | GU-like |
| TCGA-B8-5158-01A-01R-1420-07 | GU-like |
| TCGA-B0-5695-01A-11R-1541-07 | GU-like |
| TCGA-BP-5176-01A-01R-1426-07 | GU-like |
| TCGA-CZ-4859-01A-02R-1426-07 | GS-like |
| TCGA-CW-5587-01A-01R-1541-07 | GS-like |
| TCGA-B4-5844-01A-11R-1672-07 | GU-like |
| TCGA-BP-4801-01A-02R-1420-07 | GU-like |
| TCGA-A3-3307-01A-01R-0864-07 | GU-like |
| TCGA-B4-5834-01A-11R-1672-07 | GS-like |
| TCGA-G6-A8L6-01A-11R-A37O-07 | GU-like |
| TCGA-BP-4991-01A-01R-1334-07 | GU-like |
| TCGA-A3-3347-01A-02R-1325-07 | GU-like |
| TCGA-B2-5636-01A-02R-1541-07 | GS-like |
| TCGA-A3-3308-01A-02R-1325-07 | GU-like |
| TCGA-MM-A84U-01A-11R-A37O-07 | GU-like |
| TCGA-CJ-4907-01A-01R-1426-07 | GS-like |
| TCGA-BP-5196-01A-01R-1426-07 | GU-like |
| TCGA-CJ-4889-01A-01R-1305-07 | GS-like |
| TCGA-AK-3428-01A-02R-1277-07 | GU-like |
| TCGA-CJ-4874-01A-01R-1305-07 | GS-like |
| TCGA-A3-3328-01A-01R-0864-07 | GS-like |
| TCGA-CJ-4872-01A-01R-1305-07 | GU-like |
| TCGA-B8-A54K-01A-11R-A33J-07 | GS-like |
| TCGA-CJ-4873-01A-01R-1305-07 | GU-like |
| TCGA-G6-A8L7-01A-11R-A37O-07 | GU-like |
| TCGA-CZ-5467-01A-01R-1503-07 | GS-like |
| TCGA-B8-5159-01A-01R-1420-07 | GS-like |
| TCGA-A3-3358-01A-01R-1541-07 | GU-like |
| TCGA-A3-3372-01A-02R-1325-07 | GS-like |
| TCGA-BP-4974-01A-01R-1334-07 | GU-like |
| TCGA-CW-5584-01A-01R-1541-07 | GU-like |
| TCGA-B4-5838-01A-11R-1672-07 | GU-like |
| TCGA-B0-4699-01A-01R-1277-07 | GS-like |
| TCGA-B8-5550-01A-01R-1541-07 | GU-like |
| TCGA-A3-A8OW-01A-11R-A37O-07 | GS-like |
| TCGA-B2-3923-01A-02R-1325-07 | GS-like |
| TCGA-BP-4967-01A-01R-1334-07 | GU-like |
| TCGA-BP-5201-01A-01R-1426-07 | GS-like |
| TCGA-T7-A92I-01A-11R-A37O-07 | GU-like |
| TCGA-B0-5085-01A-01R-1334-07 | GS-like |
| TCGA-CZ-4853-01A-01R-1426-07 | GS-like |
| TCGA-A3-A6NI-01A-11R-A33J-07 | GU-like |
| TCGA-AK-3465-01A-02R-1325-07 | GS-like |
| TCGA-BP-4989-01A-01R-1334-07 | GS-like |
| TCGA-G6-A8L8-01A-21R-A37O-07 | GS-like |
| TCGA-BP-4162-01A-02R-1325-07 | GS-like |
| TCGA-BP-5173-01A-01R-1426-07 | GU-like |
| TCGA-B8-A8YJ-01A-13R-A39I-07 | GS-like |
| TCGA-B0-5080-01A-01R-1503-07 | GU-like |
| TCGA-BP-4976-01A-01R-1334-07 | GS-like |
| TCGA-BP-4163-01A-02R-1325-07 | GU-like |
| TCGA-CJ-4899-01A-01R-1334-07 | GS-like |
| TCGA-BP-4977-01A-01R-1334-07 | GU-like |
| TCGA-A3-3335-01A-01R-0864-07 | GU-like |
| TCGA-CJ-4634-01A-02R-1325-07 | GS-like |
| TCGA-B2-4101-01A-02R-1277-07 | GS-like |
| TCGA-BP-5185-01A-01R-1426-07 | GU-like |
| TCGA-AK-3427-01A-01R-0864-07 | GS-like |
| TCGA-BP-4998-01A-01R-1334-07 | GS-like |
| TCGA-BP-5202-01A-02R-1426-07 | GS-like |
| TCGA-B8-5164-01A-01R-1420-07 | GU-like |
| TCGA-DV-5573-01A-01R-1541-07 | GS-like |
| TCGA-B0-5697-01A-11R-1541-07 | GS-like |
| TCGA-BP-4993-01A-02R-1420-07 | GS-like |
| TCGA-B0-4817-01A-01R-1277-07 | GU-like |
| TCGA-BP-4355-01A-01R-1289-07 | GS-like |
| TCGA-A3-3359-01A-01R-0864-07 | GS-like |
| TCGA-AK-3433-01A-02R-1277-07 | GS-like |
| TCGA-B0-4827-01A-02R-1420-07 | GU-like |
| TCGA-B2-A4SR-01A-11R-A266-07 | GU-like |
| TCGA-B2-5641-01A-01R-1541-07 | GU-like |
| TCGA-CJ-4882-01A-02R-1426-07 | GU-like |
| TCGA-BP-4795-01A-02R-1420-07 | GS-like |
| TCGA-CW-5588-01A-01R-1541-07 | GU-like |
| TCGA-CZ-5464-01A-01R-1503-07 | GU-like |
| TCGA-BP-4762-01A-02R-1289-07 | GS-like |
| TCGA-B2-5633-01A-01R-1541-07 | GU-like |
| TCGA-BP-5178-01A-01R-1426-07 | GU-like |
| TCGA-B0-5115-01A-01R-1420-07 | GU-like |
| TCGA-B8-4622-01A-02R-1277-07 | GS-like |
| TCGA-B0-5102-01A-01R-1420-07 | GU-like |
| TCGA-BP-5008-01A-01R-1334-07 | GU-like |
| TCGA-CJ-4642-01B-01R-1305-07 | GU-like |
| TCGA-A3-3362-01A-02R-1325-07 | GS-like |
| TCGA-B2-5639-01A-01R-1541-07 | GU-like |
| TCGA-CZ-5458-01A-01R-1503-07 | GS-like |
| TCGA-AS-3778-01A-01R-A32Z-07 | GS-like |
| TCGA-B0-4693-01A-01R-1277-07 | GU-like |
| TCGA-CJ-4884-01A-01R-1305-07 | GU-like |
| TCGA-B0-5710-01A-11R-1672-07 | GS-like |
| TCGA-BP-4353-01A-02R-1289-07 | GU-like |
| TCGA-GK-A6C7-01A-11R-A33J-07 | GS-like |
| TCGA-BP-4761-01A-01R-1289-07 | GU-like |
| TCGA-A3-3329-01A-01R-0864-07 | GU-like |
| TCGA-BP-4332-01A-01R-1289-07 | GS-like |
| TCGA-CJ-4643-01A-02R-1325-07 | GU-like |
| TCGA-B8-A54G-01A-11R-A266-07 | GU-like |
| TCGA-BP-4961-01A-01R-1334-07 | GS-like |
| TCGA-B0-5698-01A-11R-1672-07 | GU-like |
| TCGA-BP-5199-01A-01R-1426-07 | GU-like |
| TCGA-CW-6093-01A-11R-1672-07 | GS-like |
| TCGA-B0-4700-01A-02R-1541-07 | GU-like |
| TCGA-AK-3460-01A-02R-1277-07 | GS-like |
| TCGA-AK-3458-01A-01R-1503-07 | GS-like |
| TCGA-CZ-5984-01A-11R-1672-07 | GU-like |
| TCGA-A3-3324-01A-02R-1325-07 | GU-like |
| TCGA-CJ-4912-01A-01R-1426-07 | GU-like |
| TCGA-CZ-5453-01A-01R-1503-07 | GS-like |
| TCGA-A3-3378-01A-02R-1325-07 | GU-like |
| TCGA-A3-3343-01A-01R-0864-07 | GS-like |
| TCGA-CJ-4887-01A-01R-1305-07 | GU-like |
| TCGA-DV-5574-01A-01R-1541-07 | GS-like |
| TCGA-A3-A8CQ-01A-11R-A37O-07 | GS-like |
| TCGA-B0-4816-01A-01R-1503-07 | GU-like |
| TCGA-A3-3311-01A-02R-1325-07 | GU-like |
| TCGA-CJ-4868-01A-01R-1305-07 | GU-like |
| TCGA-B8-4148-01A-02R-1325-07 | GU-like |
| TCGA-CJ-4886-01A-01R-1305-07 | GS-like |
| TCGA-AK-3429-01A-02R-1325-07 | GS-like |
| TCGA-CZ-5465-01A-01R-1503-07 | GU-like |
| TCGA-B0-4714-01A-01R-1277-07 | GS-like |
| TCGA-AK-3461-01A-02R-1277-07 | GS-like |
| TCGA-CZ-5454-01A-01R-1503-07 | GS-like |
| TCGA-BP-4790-01A-01R-1305-07 | GS-like |
| TCGA-B0-5107-01A-01R-1420-07 | GU-like |
