# Supplementary material for: Genomic Instability Promotes the Progression of Clear Cell Renal Cell Carcinoma Through Influencing the Immune Microenvironment
Source: Front Genet. 2021 Oct 12;12:706661. doi: 10.3389/fgene.2021.706661 (PMC8546190; doi:10.3389/fgene.2021.706661)
Supplement: Supplementary file 4 [file Table_4.doc]

Supplementary Table 4. The correlation between lncRNA and mRNA

| **lncRNA** | **mRNA** | **cor** | **pvalue** |
| --- | --- | --- | --- |
| AC010501.1 | ATP6V0D2 | 0.913347759 | 1.08E-211 |
| AC010501.1 | FAM189A2 | 0.886571374 | 6.31E-182 |
| AC010501.1 | EPB41L4B | 0.881890713 | 1.69E-177 |
| AC010501.1 | SYT17 | 0.878043805 | 5.35E-174 |
| AC010501.1 | TMEM38A | 0.877573337 | 1.41E-173 |
| AC010501.1 | SETMAR | 0.872428013 | 4.28E-169 |
| AC010501.1 | PFN2 | 0.867207332 | 9.68E-165 |
| AC010501.1 | TNFAIP8L3 | 0.863250647 | 1.46E-161 |
| AC010501.1 | CLNK | 0.857038572 | 9.08E-157 |
| AC010501.1 | TMEM116 | 0.854798646 | 4.28E-155 |
| KRT7-AS | LCN12 | 0.79924712 | 7.77E-121 |
| KRT7-AS | PRSS22 | 0.78044291 | 1.31E-111 |
| KRT7-AS | TACSTD2 | 0.756182154 | 5.64E-101 |
| KRT7-AS | KCNK15 | 0.708662855 | 2.21E-83 |
| KRT7-AS | KRT7 | 0.69152811 | 6.97E-78 |
| KRT7-AS | KRT17 | 0.681282009 | 8.95E-75 |
| KRT7-AS | SLC25A29 | 0.674967859 | 6.37E-73 |
| KRT7-AS | C3orf52 | 0.674911986 | 6.62E-73 |
| KRT7-AS | NECTIN4 | 0.673189213 | 2.08E-72 |
| KRT7-AS | CACNA1H | 0.666783545 | 1.38E-70 |
| IL21-AS1 | ADARB1 | 0.44741661 | 6.86E-28 |
| IL21-AS1 | SDK1 | 0.408402796 | 4.40E-23 |
| IL21-AS1 | JPH2 | 0.385515768 | 1.53E-20 |
| IL21-AS1 | ZBED6CL | 0.382895557 | 2.90E-20 |
| IL21-AS1 | RASSF2 | 0.338298698 | 6.74E-16 |
| IL21-AS1 | TCAF2 | 0.327432662 | 6.18E-15 |
| IL21-AS1 | CAV1 | 0.327335543 | 6.30E-15 |
| IL21-AS1 | CEP250 | 0.324079344 | 1.20E-14 |
| IL21-AS1 | CDCA7L | 0.316570437 | 5.19E-14 |
| IL21-AS1 | C1QTNF1 | 0.314230493 | 8.12E-14 |
| LINC01975 | CD9 | 0.858630769 | 5.64E-158 |
| LINC01975 | ATP6V0D2 | 0.84147069 | 1.09E-145 |
| LINC01975 | HEPACAM2 | 0.84084838 | 2.85E-145 |
| LINC01975 | TBC1D1 | 0.837497832 | 4.72E-143 |
| LINC01975 | SMIM5 | 0.831715019 | 2.44E-139 |
| LINC01975 | SETMAR | 0.830740674 | 9.97E-139 |
| LINC01975 | ABHD3 | 0.82914613 | 9.80E-138 |
| LINC01975 | ERP27 | 0.828759119 | 1.70E-137 |
| LINC01975 | DHRS7 | 0.827079627 | 1.83E-136 |
| LINC01975 | NKX6-1 | 0.820993888 | 8.13E-133 |
| AC100872.1 | GALNT2 | 0.579105267 | 1.37E-49 |
| AC100872.1 | STMN2 | 0.570475954 | 7.42E-48 |
| AC100872.1 | LOXL2 | 0.56273631 | 2.41E-46 |
| AC100872.1 | CRYGS | 0.562048834 | 3.27E-46 |
| AC100872.1 | EPO | 0.541605961 | 2.06E-42 |
| AC100872.1 | TNNT1 | 0.538109149 | 8.69E-42 |
| AC100872.1 | LOXL3 | 0.536197216 | 1.89E-41 |
| AC100872.1 | SYP | 0.530213367 | 2.10E-40 |
| AC100872.1 | INHBE | 0.520128037 | 1.09E-38 |
| AC100872.1 | PTPRN | 0.519243107 | 1.52E-38 |
| AL009178.2 | STMN2 | 0.849356543 | 3.82E-151 |
| AL009178.2 | CRYGS | 0.832784874 | 5.14E-140 |
| AL009178.2 | ANKRD65 | 0.826293785 | 5.51E-136 |
| AL009178.2 | LIPG | 0.822921638 | 5.88E-134 |
| AL009178.2 | SYP | 0.8175097 | 8.63E-131 |
| AL009178.2 | LOXL3 | 0.814798714 | 3.04E-129 |
| AL009178.2 | CHGB | 0.793255613 | 8.59E-118 |
| AL009178.2 | PTX3 | 0.746025468 | 6.92E-97 |
| AL009178.2 | EPO | 0.726966177 | 1.02E-89 |
| AL009178.2 | FANCE | 0.721418044 | 9.62E-88 |
| LEF1-AS1 | ATP6V1G3 | 0.923758341 | 5.42E-226 |
| LEF1-AS1 | NDUFA4 | 0.900021027 | 8.02E-196 |
| LEF1-AS1 | C9orf24 | 0.884491908 | 6.18E-180 |
| LEF1-AS1 | RHCG | 0.870155725 | 3.55E-167 |
| LEF1-AS1 | C1orf53 | 0.86322953 | 1.52E-161 |
| LEF1-AS1 | COX7B | 0.862129189 | 1.11E-160 |
| LEF1-AS1 | ATP6V0B | 0.861999964 | 1.41E-160 |
| LEF1-AS1 | MRPL34 | 0.850771986 | 3.71E-152 |
| LEF1-AS1 | PACRG | 0.848244374 | 2.35E-150 |
| LEF1-AS1 | COX6A1 | 0.845668189 | 1.49E-148 |
| LINC01558 | CAMK1G | 0.88625302 | 1.28E-181 |
| LINC01558 | PTGDS | 0.881876192 | 1.74E-177 |
| LINC01558 | HHATL | 0.857284986 | 5.92E-157 |
| LINC01558 | SEMA4A | 0.848174399 | 2.63E-150 |
| LINC01558 | ITGAM | 0.821055745 | 7.47E-133 |
| LINC01558 | C2CD4A | 0.795909269 | 3.97E-119 |
| LINC01558 | NDRG4 | 0.777617448 | 2.65E-110 |
| LINC01558 | TYRP1 | 0.771424353 | 1.67E-107 |
| LINC01558 | MFNG | 0.760105579 | 1.31E-102 |
| LINC01558 | ITGAD | 0.73606059 | 4.64E-93 |
| LINC01257 | FAM180A | 0.936249319 | 4.15E-246 |
| LINC01257 | TFF3 | 0.879793994 | 1.42E-175 |
| LINC01257 | TNNT3 | 0.814053743 | 8.01E-129 |
| LINC01257 | IGF2 | 0.803616559 | 4.02E-123 |
| LINC01257 | SCNN1G | 0.781235765 | 5.57E-112 |
| LINC01257 | SCNN1B | 0.769311554 | 1.44E-106 |
| LINC01257 | PTGS2 | 0.749519586 | 2.86E-98 |
| LINC01257 | RGS2 | 0.724152541 | 1.04E-88 |
| LINC01257 | SCUBE1 | 0.713239213 | 6.41E-85 |
| LINC01257 | IL6 | 0.683701465 | 1.70E-75 |
| AC007849.1 | ZNF91 | 0.899091722 | 8.45E-195 |
| AC007849.1 | SLC14A2 | 0.874156102 | 1.41E-170 |
| AC007849.1 | KIAA1456 | 0.862488537 | 5.82E-161 |
| AC007849.1 | PIK3C2G | 0.858941293 | 3.27E-158 |
| AC007849.1 | RNF152 | 0.84921827 | 4.79E-151 |
| AC007849.1 | ZNF431 | 0.846144881 | 6.93E-149 |
| AC007849.1 | ZNF493 | 0.843663449 | 3.56E-147 |
| AC007849.1 | DGKI | 0.827639805 | 8.31E-137 |
| AC007849.1 | NFAT5 | 0.823492807 | 2.69E-134 |
| AC007849.1 | PDE4D | 0.81426398 | 6.10E-129 |
| AC002384.1 | CCDC71L | 0.597844225 | 1.54E-53 |
| AC002384.1 | SERPINE1 | 0.495050638 | 1.14E-34 |
| AC002384.1 | KDELR2 | 0.479964799 | 2.09E-32 |
| AC002384.1 | SRPX2 | 0.477816674 | 4.30E-32 |
| AC002384.1 | NAP1L1 | 0.446314771 | 9.56E-28 |
| AC002384.1 | RCC1 | 0.438686367 | 9.27E-27 |
| AC002384.1 | FRRS1 | 0.435911189 | 2.09E-26 |
| AC002384.1 | GFPT2 | 0.427448846 | 2.37E-25 |
| AC002384.1 | GXYLT2 | 0.424724645 | 5.11E-25 |
| AC002384.1 | SHH | 0.422687247 | 9.03E-25 |
| LINC00885 | SYNGR3 | 0.864456216 | 1.61E-162 |
| LINC00885 | ADCYAP1 | 0.849025645 | 6.56E-151 |
| LINC00885 | SDCBP2 | 0.831481846 | 3.42E-139 |
| LINC00885 | SMOC1 | 0.824209059 | 1.00E-134 |
| LINC00885 | EPN3 | 0.789659689 | 5.16E-116 |
| LINC00885 | PLA2G4F | 0.789198941 | 8.66E-116 |
| LINC00885 | KLK4 | 0.77659469 | 7.80E-110 |
| LINC00885 | INPP5J | 0.775003513 | 4.13E-109 |
| LINC00885 | KCNJ11 | 0.768775322 | 2.48E-106 |
| LINC00885 | FAM189A2 | 0.763658519 | 4.08E-104 |
| HHATL-AS1 | HHATL | 0.948449571 | 3.76E-270 |
| HHATL-AS1 | CAMK1G | 0.905532538 | 4.24E-202 |
| HHATL-AS1 | PTGDS | 0.902205772 | 2.89E-198 |
| HHATL-AS1 | SEMA4A | 0.869900584 | 5.80E-167 |
| HHATL-AS1 | ITGAM | 0.849366259 | 3.76E-151 |
| HHATL-AS1 | SLC19A3 | 0.819449674 | 6.51E-132 |
| HHATL-AS1 | RHOBTB2 | 0.81600601 | 6.27E-130 |
| HHATL-AS1 | TYRP1 | 0.801912362 | 3.18E-122 |
| HHATL-AS1 | NDRG4 | 0.797750718 | 4.57E-120 |
| HHATL-AS1 | MFNG | 0.795971145 | 3.69E-119 |
| AC087564.1 | MYO1G | 0.539711893 | 4.50E-42 |
| AC087564.1 | MT1X | 0.531059871 | 1.50E-40 |
| AC087564.1 | MT1E | 0.522692525 | 4.03E-39 |
| AC087564.1 | FGA | 0.514603906 | 8.92E-38 |
| AC087564.1 | PROS1 | 0.482598218 | 8.57E-33 |
| AC087564.1 | ROM1 | 0.482549489 | 8.72E-33 |
| AC087564.1 | FGB | 0.480497175 | 1.75E-32 |
| AC087564.1 | MT2A | 0.466456712 | 1.79E-30 |
| AC087564.1 | ZMYND8 | 0.445239254 | 1.32E-27 |
| AC087564.1 | RCL1 | 0.435116423 | 2.63E-26 |
| AC087636.1 | CXCL6 | 0.699913637 | 1.59E-80 |
| AC087636.1 | PF4V1 | 0.659759965 | 1.21E-68 |
| AC087636.1 | CXCL1 | 0.634234816 | 5.35E-62 |
| AC087636.1 | SAA2-SAA4 | 0.607478344 | 1.13E-55 |
| AC087636.1 | MMP25 | 0.60494499 | 4.19E-55 |
| AC087636.1 | FCGR3B | 0.577680922 | 2.67E-49 |
| AC087636.1 | SAA4 | 0.571003217 | 5.83E-48 |
| AC087636.1 | SAA1 | 0.557162467 | 2.80E-45 |
| AC087636.1 | CSF3R | 0.52499556 | 1.64E-39 |
| AC087636.1 | RNLS | 0.501169615 | 1.28E-35 |
| AL136088.1 | CLCNKA | 0.862443848 | 6.31E-161 |
| AL136088.1 | FOXI1 | 0.80380281 | 3.21E-123 |
| AL136088.1 | FA2H | 0.798674641 | 1.53E-120 |
| AL136088.1 | SYNGR3 | 0.797794919 | 4.34E-120 |
| AL136088.1 | KCNJ11 | 0.797137084 | 9.41E-120 |
| AL136088.1 | FAM189A2 | 0.795466897 | 6.64E-119 |
| AL136088.1 | DUSP15 | 0.790899661 | 1.27E-116 |
| AL136088.1 | CCDC151 | 0.788581777 | 1.73E-115 |
| AL136088.1 | C15orf59 | 0.787408709 | 6.43E-115 |
| AL136088.1 | SDCBP2 | 0.786808531 | 1.25E-114 |
| PART1 | CLDN8 | 0.882757078 | 2.65E-178 |
| PART1 | TSPAN5 | 0.853905725 | 1.95E-154 |
| PART1 | HEPACAM2 | 0.840704955 | 3.56E-145 |
| PART1 | SETMAR | 0.840595332 | 4.21E-145 |
| PART1 | TMEM116 | 0.833049231 | 3.49E-140 |
| PART1 | MYZAP | 0.827585897 | 8.96E-137 |
| PART1 | ATP6V0D2 | 0.820178296 | 2.44E-132 |
| PART1 | PFN2 | 0.818954406 | 1.26E-131 |
| PART1 | SC5D | 0.814890032 | 2.70E-129 |
| PART1 | TNFAIP8L3 | 0.814515811 | 4.40E-129 |
| AC022148.1 | BAAT | 0.958075916 | 1.10E-293 |
| AC022148.1 | BZW2 | 0.945423016 | 1.12E-263 |
| AC022148.1 | PIGZ | 0.921970505 | 2.13E-223 |
| AC022148.1 | GPBAR1 | 0.918192526 | 4.12E-218 |
| AC022148.1 | IRX1 | 0.918029838 | 6.87E-218 |
| AC022148.1 | CAPN3 | 0.898621892 | 2.75E-194 |
| AC022148.1 | BBS4 | 0.896350507 | 7.67E-192 |
| AC022148.1 | ZNF114 | 0.893980523 | 2.38E-189 |
| AC022148.1 | MYO7B | 0.881982053 | 1.39E-177 |
| AC022148.1 | SFXN2 | 0.877663321 | 1.17E-173 |
| AC116312.1 | MUC20 | 0.873249724 | 8.49E-170 |
| AC116312.1 | RHBG | 0.859106062 | 2.44E-158 |
| AC116312.1 | NTN1 | 0.85141311 | 1.28E-152 |
| AC116312.1 | PIP | 0.848821676 | 9.16E-151 |
| AC116312.1 | NKX6-1 | 0.809640238 | 2.28E-126 |
| AC116312.1 | FOXI2 | 0.806280244 | 1.52E-124 |
| AC116312.1 | SMIM5 | 0.804918061 | 8.18E-124 |
| AC116312.1 | ERP27 | 0.79549393 | 6.44E-119 |
| AC116312.1 | ABHD3 | 0.762489008 | 1.29E-103 |
| AC116312.1 | SLC4A9 | 0.756808388 | 3.11E-101 |
| AC010329.1 | BMPR1B | 0.896338228 | 7.91E-192 |
| AC010329.1 | RSPO3 | 0.864707312 | 1.01E-162 |
| AC010329.1 | MYZAP | 0.86270963 | 3.90E-161 |
| AC010329.1 | ZDHHC23 | 0.859060502 | 2.65E-158 |
| AC010329.1 | GPAM | 0.854735445 | 4.77E-155 |
| AC010329.1 | DGKI | 0.854012987 | 1.63E-154 |
| AC010329.1 | PIK3C2G | 0.852846867 | 1.17E-153 |
| AC010329.1 | STXBP6 | 0.846669096 | 2.99E-149 |
| AC010329.1 | ATP8B4 | 0.842265471 | 3.17E-146 |
| AC010329.1 | BTG3 | 0.842074229 | 4.27E-146 |
| AC129507.4 | BCAS4 | 0.687325456 | 1.36E-76 |
| AC129507.4 | ADCYAP1 | 0.680074239 | 2.04E-74 |
| AC129507.4 | RHCG | 0.680073718 | 2.04E-74 |
| AC129507.4 | CCDC151 | 0.670317785 | 1.38E-71 |
| AC129507.4 | DUSP15 | 0.668209984 | 5.46E-71 |
| AC129507.4 | KLK15 | 0.659677055 | 1.27E-68 |
| AC129507.4 | KCNQ1 | 0.657598889 | 4.68E-68 |
| AC129507.4 | SYNGR3 | 0.656249163 | 1.08E-67 |
| AC129507.4 | KLK4 | 0.655910505 | 1.34E-67 |
| AC129507.4 | DEFB1 | 0.646638875 | 3.79E-65 |
| AC108860.2 | TJP3 | 0.851343088 | 1.44E-152 |
| AC108860.2 | ESRP1 | 0.786341238 | 2.10E-114 |
| AC108860.2 | EPB41L4B | 0.77880075 | 7.56E-111 |
| AC108860.2 | TMEM30B | 0.778245971 | 1.36E-110 |
| AC108860.2 | GRHL2 | 0.764945615 | 1.14E-104 |
| AC108860.2 | STAP1 | 0.764379419 | 2.00E-104 |
| AC108860.2 | FHDC1 | 0.763525769 | 4.65E-104 |
| AC108860.2 | FAM3B | 0.761755565 | 2.64E-103 |
| AC108860.2 | ATP6V0D2 | 0.761265322 | 4.26E-103 |
| AC108860.2 | SYT17 | 0.756607328 | 3.77E-101 |
| AC015660.1 | KCNIP1 | 0.746395313 | 4.95E-97 |
| AC015660.1 | TDGF1 | 0.716525895 | 4.83E-86 |
| AC015660.1 | B4GALNT1 | 0.623502725 | 2.18E-59 |
| AC015660.1 | COX4I2 | 0.579553759 | 1.11E-49 |
| AC015660.1 | SHISA3 | 0.575851935 | 6.25E-49 |
| AC015660.1 | TMCO4 | 0.572475412 | 2.97E-48 |
| AC015660.1 | PDE8B | 0.554983811 | 7.21E-45 |
| AC015660.1 | ANKS1A | 0.55496431 | 7.27E-45 |
| AC015660.1 | FLNB | 0.553158438 | 1.58E-44 |
| AC015660.1 | MYO3B | 0.553103081 | 1.62E-44 |
| LINC00856 | CCRL2 | 0.637099783 | 1.03E-62 |
| LINC00856 | GABARAPL1 | 0.631368176 | 2.73E-61 |
| LINC00856 | LINC00890 | 0.619453716 | 1.99E-58 |
| LINC00856 | TMEM38A | 0.588227193 | 1.76E-51 |
| LINC00856 | FAM189A2 | 0.583396983 | 1.80E-50 |
| LINC00856 | ZNF165 | 0.581633905 | 4.15E-50 |
| LINC00856 | CWH43 | 0.580932707 | 5.79E-50 |
| LINC00856 | DIRAS1 | 0.574430504 | 1.21E-48 |
| LINC00856 | CLCNKB | 0.565718176 | 6.38E-47 |
| LINC00856 | TNFAIP8L3 | 0.561937278 | 3.44E-46 |
| AC105094.2 | RNF152 | 0.776028867 | 1.41E-109 |
| AC105094.2 | PKIA | 0.766656668 | 2.08E-105 |
| AC105094.2 | SLC16A7 | 0.748466635 | 7.51E-98 |
| AC105094.2 | PIK3C2G | 0.740282061 | 1.17E-94 |
| AC105094.2 | MPPED2 | 0.737088425 | 1.90E-93 |
| AC105094.2 | SLC14A2 | 0.73570208 | 6.32E-93 |
| AC105094.2 | TMEM116 | 0.729621929 | 1.11E-90 |
| AC105094.2 | BMPR1B | 0.726216208 | 1.90E-89 |
| AC105094.2 | KIAA1456 | 0.725826614 | 2.62E-89 |
| AC105094.2 | CLNK | 0.720915626 | 1.44E-87 |
| SALRNA1 | SLC14A2 | 0.914879775 | 1.11E-213 |
| SALRNA1 | RNF152 | 0.914330812 | 5.76E-213 |
| SALRNA1 | PIK3C2G | 0.904141941 | 1.77E-200 |
| SALRNA1 | DGKI | 0.896822734 | 2.41E-192 |
| SALRNA1 | KIAA1456 | 0.889432239 | 9.92E-185 |
| SALRNA1 | ZNF91 | 0.885484645 | 7.00E-181 |
| SALRNA1 | ZNF431 | 0.876688367 | 8.59E-173 |
| SALRNA1 | SLC16A7 | 0.864362563 | 1.91E-162 |
| SALRNA1 | SAMD12 | 0.851806148 | 6.66E-153 |
| SALRNA1 | EXPH5 | 0.835839347 | 5.67E-142 |
| AC103563.2 | CLNK | 0.864481223 | 1.54E-162 |
| AC103563.2 | C1orf168 | 0.863041051 | 2.14E-161 |
| AC103563.2 | TFCP2L1 | 0.862819202 | 3.20E-161 |
| AC103563.2 | MSI2 | 0.83623153 | 3.16E-142 |
| AC103563.2 | SLC26A7 | 0.829317186 | 7.68E-138 |
| AC103563.2 | MPPED2 | 0.821982622 | 2.12E-133 |
| AC103563.2 | SLC16A7 | 0.819371926 | 7.22E-132 |
| AC103563.2 | KLHL3 | 0.808923138 | 5.62E-126 |
| AC103563.2 | CCDC68 | 0.807433719 | 3.63E-125 |
| AC103563.2 | PPARGC1A | 0.807328509 | 4.14E-125 |
| AC128687.2 | ZDHHC3 | 0.744735842 | 2.21E-96 |
| AC128687.2 | MYRIP | 0.73503812 | 1.12E-92 |
| AC128687.2 | RAB11FIP4 | 0.733656851 | 3.66E-92 |
| AC128687.2 | ANKRD9 | 0.727755717 | 5.30E-90 |
| AC128687.2 | KLK15 | 0.718850326 | 7.59E-87 |
| AC128687.2 | BDH1 | 0.701117977 | 6.52E-81 |
| AC128687.2 | SETMAR | 0.694641113 | 7.47E-79 |
| AC128687.2 | EPB41L4B | 0.684770209 | 8.09E-76 |
| AC128687.2 | NR0B2 | 0.683738559 | 1.65E-75 |
| AC128687.2 | INPP5J | 0.676175911 | 2.84E-73 |
| GAS6-AS2 | ATP6V0D2 | 0.87414793 | 1.43E-170 |
| GAS6-AS2 | TBC1D1 | 0.848834812 | 8.97E-151 |
| GAS6-AS2 | PXK | 0.840716584 | 3.50E-145 |
| GAS6-AS2 | PACRG | 0.833660899 | 1.42E-140 |
| GAS6-AS2 | SETMAR | 0.827353411 | 1.24E-136 |
| GAS6-AS2 | DMRT2 | 0.822298593 | 1.38E-133 |
| GAS6-AS2 | SYT17 | 0.814105805 | 7.49E-129 |
| GAS6-AS2 | ABHD3 | 0.812974543 | 3.23E-128 |
| GAS6-AS2 | CFTR | 0.811042809 | 3.84E-127 |
| GAS6-AS2 | SMIM5 | 0.804925667 | 8.10E-124 |
| AL356740.3 | ZNF808 | 0.648572687 | 1.19E-65 |
| AL356740.3 | TNRC6B | 0.641207997 | 9.45E-64 |
| AL356740.3 | ZNF552 | 0.628198229 | 1.62E-60 |
| AL356740.3 | ZBTB20 | 0.625481777 | 7.34E-60 |
| AL356740.3 | AP001931.1 | 0.625027052 | 9.43E-60 |
| AL356740.3 | INTS6 | 0.621644417 | 6.04E-59 |
| AL356740.3 | OXNAD1 | 0.620673468 | 1.03E-58 |
| AL356740.3 | ZNF460 | 0.619271776 | 2.19E-58 |
| AL356740.3 | MYOC | 0.616219916 | 1.13E-57 |
| AL356740.3 | ATP8B4 | 0.611882444 | 1.13E-56 |
| AC005307.1 | PTPRO | 0.517011237 | 3.58E-38 |
| AC005307.1 | APLP1 | 0.496852012 | 6.01E-35 |
| AC005307.1 | IBSP | 0.467901742 | 1.12E-30 |
| AC005307.1 | TM4SF4 | 0.457719004 | 2.88E-29 |
| AC005307.1 | ST3GAL5 | 0.457435589 | 3.14E-29 |
| AC005307.1 | CPA4 | 0.449002965 | 4.24E-28 |
| AC005307.1 | FGF2 | 0.413207955 | 1.22E-23 |
| AC005307.1 | NTM | 0.391060728 | 3.86E-21 |
| AC005307.1 | DCBLD2 | 0.373037469 | 3.07E-19 |
| AC005307.1 | GPR39 | 0.361464979 | 4.43E-18 |
| AC024563.1 | BMPR1B | 0.846477988 | 4.06E-149 |
| AC024563.1 | RNF152 | 0.846381114 | 4.75E-149 |
| AC024563.1 | PIK3C2G | 0.838336673 | 1.33E-143 |
| AC024563.1 | KIAA1456 | 0.834196715 | 6.47E-141 |
| AC024563.1 | SLC16A7 | 0.833862674 | 1.06E-140 |
| AC024563.1 | SLC14A2 | 0.832878574 | 4.48E-140 |
| AC024563.1 | ZNF431 | 0.822309877 | 1.36E-133 |
| AC024563.1 | DGKI | 0.818165626 | 3.61E-131 |
| AC024563.1 | PKIA | 0.810802934 | 5.22E-127 |
| AC024563.1 | SAMD12 | 0.809164365 | 4.15E-126 |
| AL356740.1 | ABHD3 | 0.825038343 | 3.17E-135 |
| AL356740.1 | ATP6V0D2 | 0.801699369 | 4.12E-122 |
| AL356740.1 | ERP27 | 0.786645528 | 1.50E-114 |
| AL356740.1 | NTN1 | 0.767359866 | 1.03E-105 |
| AL356740.1 | CD9 | 0.759106611 | 3.44E-102 |
| AL356740.1 | FOXI2 | 0.749847616 | 2.11E-98 |
| AL356740.1 | PLIN5 | 0.749784825 | 2.24E-98 |
| AL356740.1 | SYT17 | 0.748784876 | 5.61E-98 |
| AL356740.1 | RHBG | 0.746760927 | 3.55E-97 |
| AL356740.1 | RALGPS1 | 0.743410909 | 7.26E-96 |
| AL928921.1 | ENO3 | 0.924578355 | 3.33E-227 |
| AL928921.1 | CYP17A1 | 0.905114464 | 1.31E-201 |
| AL928921.1 | LYPD6B | 0.856686119 | 1.67E-156 |
| AL928921.1 | PAH | 0.843681287 | 3.46E-147 |
| AL928921.1 | CAPN3 | 0.808990862 | 5.17E-126 |
| AL928921.1 | ADGRB1 | 0.794314331 | 2.53E-118 |
| AL928921.1 | MYO7B | 0.77912362 | 5.36E-111 |
| AL928921.1 | SLC51B | 0.768157702 | 4.62E-106 |
| AL928921.1 | COLEC11 | 0.759243211 | 3.02E-102 |
| AL928921.1 | NMRK2 | 0.750596 | 1.06E-98 |
| LINC02315 | PDPN | 0.38633679 | 1.25E-20 |
| LINC02315 | FOSL1 | 0.378064779 | 9.31E-20 |
| LINC02315 | CDKN2A | 0.377797679 | 9.92E-20 |
| LINC02315 | SRPX | 0.37323255 | 2.93E-19 |
| LINC02315 | DKK1 | 0.341796908 | 3.24E-16 |
| LINC02315 | HMGA1 | 0.339082351 | 5.72E-16 |
| LINC02315 | LAMP5 | 0.33180919 | 2.56E-15 |
| LINC02315 | PRSS3 | 0.324759328 | 1.05E-14 |
| LINC02315 | SLC20A1 | 0.319904458 | 2.73E-14 |
| LINC02315 | TMEM158 | 0.315292547 | 6.63E-14 |
| AP001046.1 | BTG3 | 0.822087423 | 1.84E-133 |
| AP001046.1 | BMPR1B | 0.797416124 | 6.78E-120 |
| AP001046.1 | RSPO3 | 0.794075652 | 3.34E-118 |
| AP001046.1 | GPAM | 0.761234371 | 4.39E-103 |
| AP001046.1 | TSPAN5 | 0.760396687 | 9.89E-103 |
| AP001046.1 | PSCA | 0.758518078 | 6.06E-102 |
| AP001046.1 | PKIA | 0.757306397 | 1.93E-101 |
| AP001046.1 | ACSS3 | 0.747112155 | 2.58E-97 |
| AP001046.1 | OXCT1 | 0.745471254 | 1.14E-96 |
| AP001046.1 | FAM3B | 0.743837248 | 4.96E-96 |
| LINC01489 | GPD1L | 0.738853286 | 4.09E-94 |
| LINC01489 | STK32A | 0.73342642 | 4.45E-92 |
| LINC01489 | OXCT1 | 0.713751463 | 4.30E-85 |
| LINC01489 | KLHL3 | 0.704118521 | 6.94E-82 |
| LINC01489 | TBC1D1 | 0.687705764 | 1.04E-76 |
| LINC01489 | ERP27 | 0.677700679 | 1.02E-73 |
| LINC01489 | EXOSC7 | 0.662607323 | 2.00E-69 |
| LINC01489 | PFN2 | 0.661616501 | 3.75E-69 |
| LINC01489 | SETMAR | 0.651412989 | 2.12E-66 |
| LINC01489 | MYRIP | 0.649383461 | 7.27E-66 |
| AC068057.1 | MYO3B | 0.665795361 | 2.60E-70 |
| AC068057.1 | CASP14 | 0.658946794 | 2.02E-68 |
| AC068057.1 | VTCN1 | 0.636845362 | 1.19E-62 |
| AC068057.1 | HOXB8 | 0.601379882 | 2.59E-54 |
| AC068057.1 | ITM2C | 0.580791901 | 6.19E-50 |
| AC068057.1 | TMCO4 | 0.571242304 | 5.23E-48 |
| AC068057.1 | SUSD4 | 0.566598786 | 4.29E-47 |
| AC068057.1 | KCNJ1 | 0.55620692 | 4.24E-45 |
| AC068057.1 | EHF | 0.552997632 | 1.70E-44 |
| AC068057.1 | SLC7A1 | 0.548323321 | 1.24E-43 |
| IGF2-AS | INHA | 0.774036676 | 1.13E-108 |
| IGF2-AS | B4GALNT1 | 0.725343655 | 3.90E-89 |
| IGF2-AS | IGF2 | 0.714990088 | 1.62E-85 |
| IGF2-AS | FLNB | 0.67120919 | 7.68E-72 |
| IGF2-AS | OBSL1 | 0.641842543 | 6.51E-64 |
| IGF2-AS | ECEL1 | 0.638531917 | 4.50E-63 |
| IGF2-AS | ANKS1A | 0.634926308 | 3.60E-62 |
| IGF2-AS | KCNIP1 | 0.598088964 | 1.36E-53 |
| IGF2-AS | WWP2 | 0.59337651 | 1.42E-52 |
| IGF2-AS | PDE8B | 0.592126524 | 2.63E-52 |
| AC015921.1 | NNAT | 0.817187998 | 1.32E-130 |
| AC015921.1 | ATP5EP2 | 0.763190883 | 6.47E-104 |
| AC015921.1 | RAD51B | 0.750496638 | 1.16E-98 |
| AC015921.1 | TFF1 | 0.737612372 | 1.21E-93 |
| AC015921.1 | FSIP1 | 0.72793403 | 4.57E-90 |
| AC015921.1 | FAM221A | 0.723066148 | 2.52E-88 |
| AC015921.1 | GATA3 | 0.708410308 | 2.68E-83 |
| AC015921.1 | HARBI1 | 0.708127152 | 3.33E-83 |
| AC015921.1 | CMTM1 | 0.706350748 | 1.29E-82 |
| AC015921.1 | ESR1 | 0.703685446 | 9.60E-82 |
| AC100801.1 | COX6C | 0.724909994 | 5.58E-89 |
| AC100801.1 | ATP5EP2 | 0.703522745 | 1.08E-81 |
| AC100801.1 | NRIP3 | 0.700377177 | 1.13E-80 |
| AC100801.1 | STARD10 | 0.665858001 | 2.50E-70 |
| AC100801.1 | GATA3 | 0.650160478 | 4.54E-66 |
| AC100801.1 | TFF1 | 0.644448882 | 1.40E-64 |
| AC100801.1 | NNAT | 0.617146055 | 6.90E-58 |
| AC100801.1 | HARBI1 | 0.613820554 | 4.07E-57 |
| AC100801.1 | RHCG | 0.604906597 | 4.27E-55 |
| AC100801.1 | FSIP1 | 0.601218412 | 2.81E-54 |
| AL139280.1 | PRSS22 | 0.758593712 | 5.64E-102 |
| AL139280.1 | CACNA1H | 0.729286714 | 1.48E-90 |
| AL139280.1 | ABCA4 | 0.723073236 | 2.51E-88 |
| AL139280.1 | TACSTD2 | 0.7199473 | 3.15E-87 |
| AL139280.1 | USP43 | 0.713892594 | 3.85E-85 |
| AL139280.1 | AGR2 | 0.706912525 | 8.40E-83 |
| AL139280.1 | PADI1 | 0.686114346 | 3.18E-76 |
| AL139280.1 | TMPRSS4 | 0.657156434 | 6.17E-68 |
| AL139280.1 | SFN | 0.653563403 | 5.69E-67 |
| AL139280.1 | KRT17 | 0.650262067 | 4.27E-66 |
| AL161668.3 | SPINK1 | 0.767097046 | 1.34E-105 |
| AL161668.3 | PHYH | 0.757505614 | 1.60E-101 |
| AL161668.3 | CALCA | 0.756999254 | 2.59E-101 |
| AL161668.3 | SLC25A5 | 0.742895912 | 1.15E-95 |
| AL161668.3 | ATP6V1G3 | 0.730146958 | 7.17E-91 |
| AL161668.3 | RHCG | 0.725958811 | 2.35E-89 |
| AL161668.3 | PVALB | 0.723374564 | 1.96E-88 |
| AL161668.3 | INSIG1 | 0.716192704 | 6.29E-86 |
| AL161668.3 | DNASE1 | 0.704217348 | 6.44E-82 |
| AL161668.3 | CA2 | 0.699087172 | 2.92E-80 |
| AL035661.1 | MYZAP | 0.869488584 | 1.28E-166 |
| AL035661.1 | RHCG | 0.867299685 | 8.14E-165 |
| AL035661.1 | EPB41L4B | 0.858538274 | 6.63E-158 |
| AL035661.1 | TMEM38A | 0.855516504 | 1.25E-155 |
| AL035661.1 | TMEM116 | 0.852305262 | 2.89E-153 |
| AL035661.1 | SETMAR | 0.850573255 | 5.16E-152 |
| AL035661.1 | PFN2 | 0.842803602 | 1.37E-146 |
| AL035661.1 | TMEM30B | 0.836320017 | 2.77E-142 |
| AL035661.1 | HEPACAM2 | 0.834717258 | 3.00E-141 |
| AL035661.1 | GLTP | 0.825848087 | 1.03E-135 |
| THRB-IT1 | SLC14A2 | 0.898029629 | 1.21E-193 |
| THRB-IT1 | ZNF91 | 0.890779401 | 4.46E-186 |
| THRB-IT1 | PIK3C2G | 0.857962315 | 1.82E-157 |
| THRB-IT1 | KIAA1456 | 0.852323967 | 2.80E-153 |
| THRB-IT1 | RNF152 | 0.844551301 | 8.76E-148 |
| THRB-IT1 | ZNF431 | 0.836683891 | 1.60E-142 |
| THRB-IT1 | ZNF493 | 0.831654215 | 2.66E-139 |
| THRB-IT1 | DGKI | 0.82703151 | 1.96E-136 |
| THRB-IT1 | NFAT5 | 0.814356209 | 5.41E-129 |
| THRB-IT1 | PDE4D | 0.807821701 | 2.24E-125 |
| LINC01561 | PLPP4 | 0.879543498 | 2.39E-175 |
| LINC01561 | ABHD17C | 0.627788974 | 2.04E-60 |
| LINC01561 | ADCK1 | 0.624151242 | 1.53E-59 |
| LINC01561 | AHSA1 | 0.602188574 | 1.72E-54 |
| LINC01561 | CREB3L1 | 0.594712725 | 7.34E-53 |
| LINC01561 | MAP3K9 | 0.594037541 | 1.03E-52 |
| LINC01561 | GSTZ1 | 0.583521472 | 1.69E-50 |
| LINC01561 | COX16 | 0.553626379 | 1.30E-44 |
| LINC01561 | FAM222A | 0.544320466 | 6.68E-43 |
| LINC01561 | SEC13 | 0.542236607 | 1.59E-42 |
| AC104984.4 | ERP27 | 0.899090949 | 8.46E-195 |
| AC104984.4 | FOXI2 | 0.875524201 | 9.08E-172 |
| AC104984.4 | NTN1 | 0.871692928 | 1.80E-168 |
| AC104984.4 | GNPNAT1 | 0.856643449 | 1.80E-156 |
| AC104984.4 | ABHD3 | 0.846104608 | 7.39E-149 |
| AC104984.4 | RHBG | 0.841136247 | 1.83E-145 |
| AC104984.4 | ATP6V0D2 | 0.832798577 | 5.04E-140 |
| AC104984.4 | MSI2 | 0.82856063 | 2.25E-137 |
| AC104984.4 | CLNK | 0.827761418 | 7.00E-137 |
| AC104984.4 | SYT17 | 0.820995036 | 8.11E-133 |
| NCOA7-AS1 | ZNF91 | 0.931911897 | 1.08E-238 |
| NCOA7-AS1 | ZNF431 | 0.90728364 | 3.56E-204 |
| NCOA7-AS1 | SLC16A7 | 0.906686159 | 1.84E-203 |
| NCOA7-AS1 | ZNF493 | 0.90556178 | 3.92E-202 |
| NCOA7-AS1 | RNF152 | 0.903964668 | 2.83E-200 |
| NCOA7-AS1 | KIAA1456 | 0.897269727 | 7.99E-193 |
| NCOA7-AS1 | HS6ST3 | 0.890166351 | 1.84E-185 |
| NCOA7-AS1 | PIK3C2G | 0.887230157 | 1.45E-182 |
| NCOA7-AS1 | EXPH5 | 0.884653189 | 4.34E-180 |
| NCOA7-AS1 | DGKI | 0.874395756 | 8.72E-171 |
| AP001476.1 | BAAT | 0.890437582 | 9.84E-186 |
| AP001476.1 | BZW2 | 0.872955937 | 1.52E-169 |
| AP001476.1 | IRX1 | 0.855381742 | 1.58E-155 |
| AP001476.1 | GPBAR1 | 0.845772035 | 1.26E-148 |
| AP001476.1 | PIGZ | 0.833162385 | 2.96E-140 |
| AP001476.1 | ZNF114 | 0.82702599 | 1.97E-136 |
| AP001476.1 | ZDHHC3 | 0.808409995 | 1.07E-125 |
| AP001476.1 | BBS4 | 0.807399312 | 3.79E-125 |
| AP001476.1 | CAPN3 | 0.801816097 | 3.58E-122 |
| AP001476.1 | MYO7B | 0.800756353 | 1.28E-121 |
| AL121827.1 | PAPPA2 | 0.544088565 | 7.36E-43 |
| AL121827.1 | CIB4 | 0.437496946 | 1.31E-26 |
| AL121827.1 | PLCH2 | 0.360355415 | 5.69E-18 |
| AL121827.1 | ANKRD37 | 0.34003644 | 4.69E-16 |
| AL121827.1 | CETP | 0.318015924 | 3.93E-14 |
| AL121827.1 | ARG2 | 0.306885314 | 3.22E-13 |
| AL121827.1 | CD300LG | 0.29441749 | 3.07E-12 |
| AL121827.1 | PROM2 | 0.28939571 | 7.37E-12 |
| AL121827.1 | KLHDC7B | 0.286303925 | 1.25E-11 |
| AL121827.1 | PKLR | 0.286275446 | 1.26E-11 |
| AL356740.2 | ZBTB20 | 0.755240209 | 1.38E-100 |
| AL356740.2 | MYOC | 0.753232629 | 9.11E-100 |
| AL356740.2 | C5orf56 | 0.746437247 | 4.77E-97 |
| AL356740.2 | TNRC6B | 0.742490704 | 1.65E-95 |
| AL356740.2 | AP001931.1 | 0.735900382 | 5.32E-93 |
| AL356740.2 | SHPRH | 0.733752509 | 3.37E-92 |
| AL356740.2 | ZNF808 | 0.732264178 | 1.20E-91 |
| AL356740.2 | ZNF460 | 0.725601792 | 3.16E-89 |
| AL356740.2 | C5orf42 | 0.719385047 | 4.95E-87 |
| AL356740.2 | ZNF587B | 0.718957637 | 6.97E-87 |
| AC079466.1 | EPB41 | 0.251631302 | 3.14E-09 |
| AC079466.1 | ZNF813 | -0.248545419 | 4.95E-09 |
| AC079466.1 | MYEOV | 0.241438101 | 1.37E-08 |
| AC079466.1 | PTGS1 | 0.239526327 | 1.80E-08 |
| AC079466.1 | ANGPTL3 | 0.232863411 | 4.52E-08 |
| AC079466.1 | LGALS4 | 0.204328979 | 1.72E-06 |
| AC079466.1 | GAS2L3 | 0.203955983 | 1.80E-06 |
| AC079466.1 | KLHDC7A | 0.200579735 | 2.68E-06 |
| AC079466.1 | SLC2A5 | 0.19962233 | 3.00E-06 |
| AC079466.1 | KCNK9 | 0.19337942 | 6.13E-06 |
| MNX1-AS1 | PI3 | 0.529297069 | 3.02E-40 |
| MNX1-AS1 | PKP3 | 0.519460401 | 1.40E-38 |
| MNX1-AS1 | C1QL1 | 0.498699138 | 3.11E-35 |
| MNX1-AS1 | PANX2 | 0.494902203 | 1.20E-34 |
| MNX1-AS1 | TARBP2 | 0.482467196 | 8.96E-33 |
| MNX1-AS1 | QSOX1 | 0.478509119 | 3.41E-32 |
| MNX1-AS1 | DNTTIP1 | 0.477064349 | 5.53E-32 |
| MNX1-AS1 | RIN1 | 0.47271827 | 2.33E-31 |
| MNX1-AS1 | FKBP10 | 0.468285146 | 9.93E-31 |
| MNX1-AS1 | SLC35C2 | 0.460458836 | 1.22E-29 |
| C15orf59-AS1 | RHCG | 0.890420106 | 1.02E-185 |
| C15orf59-AS1 | PVALB | 0.867615427 | 4.49E-165 |
| C15orf59-AS1 | DNASE1 | 0.866755686 | 2.26E-164 |
| C15orf59-AS1 | ATP6V1G3 | 0.859685963 | 8.78E-159 |
| C15orf59-AS1 | CA2 | 0.831534983 | 3.16E-139 |
| C15orf59-AS1 | NRIP3 | 0.817777872 | 6.05E-131 |
| C15orf59-AS1 | SLC25A5 | 0.814471626 | 4.66E-129 |
| C15orf59-AS1 | NDUFA4 | 0.808430066 | 1.04E-125 |
| C15orf59-AS1 | SLC16A11 | 0.7984843 | 1.92E-120 |
| C15orf59-AS1 | DUSP15 | 0.792979645 | 1.18E-117 |
| PACRG-AS1 | C15orf59 | 0.842624373 | 1.81E-146 |
| PACRG-AS1 | PLA2G4F | 0.828176012 | 3.89E-137 |
| PACRG-AS1 | CLNK | 0.824594511 | 5.87E-135 |
| PACRG-AS1 | FOXI1 | 0.821531259 | 3.92E-133 |
| PACRG-AS1 | FHDC1 | 0.817654593 | 7.13E-131 |
| PACRG-AS1 | ATP6V0D2 | 0.814432668 | 4.90E-129 |
| PACRG-AS1 | EPN3 | 0.811397996 | 2.44E-127 |
| PACRG-AS1 | CFTR | 0.804030734 | 2.43E-123 |
| PACRG-AS1 | SMOC1 | 0.801760159 | 3.83E-122 |
| PACRG-AS1 | FGF9 | 0.799270536 | 7.55E-121 |
| AC090241.2 | HS6ST3 | 0.955074613 | 8.47E-286 |
| AC090241.2 | SLC16A7 | 0.915384006 | 2.41E-214 |
| AC090241.2 | PIK3C2G | 0.912710863 | 7.03E-211 |
| AC090241.2 | RNF152 | 0.905107668 | 1.33E-201 |
| AC090241.2 | DGKI | 0.898534675 | 3.43E-194 |
| AC090241.2 | PPM1L | 0.894566023 | 5.84E-190 |
| AC090241.2 | ZNF431 | 0.892616629 | 6.08E-188 |
| AC090241.2 | BMPR1B | 0.890211309 | 1.66E-185 |
| AC090241.2 | LIN7A | 0.887851178 | 3.59E-183 |
| AC090241.2 | EXPH5 | 0.88499702 | 2.05E-180 |
| AC008514.1 | IRX2 | 0.759875139 | 1.64E-102 |
| AC008514.1 | SCUBE2 | 0.72069337 | 1.73E-87 |
| AC008514.1 | ATP5EP2 | 0.694496757 | 8.29E-79 |
| AC008514.1 | TFF1 | 0.674401375 | 9.30E-73 |
| AC008514.1 | FSIP1 | 0.655212189 | 2.06E-67 |
| AC008514.1 | RAD51B | 0.653039953 | 7.85E-67 |
| AC008514.1 | ASIP | 0.639643559 | 2.36E-63 |
| AC008514.1 | GATA3 | 0.634428502 | 4.79E-62 |
| AC008514.1 | FASN | 0.632390235 | 1.53E-61 |
| AC008514.1 | CMTM1 | 0.628008972 | 1.80E-60 |
| AL121820.2 | NECTIN4 | 0.67748649 | 1.18E-73 |
| AL121820.2 | GALNT3 | 0.663800949 | 9.34E-70 |
| AL121820.2 | TACSTD2 | 0.649614869 | 6.32E-66 |
| AL121820.2 | PTK7 | 0.636677006 | 1.32E-62 |
| AL121820.2 | MYO3B | 0.625056413 | 9.28E-60 |
| AL121820.2 | CNMD | 0.619480841 | 1.96E-58 |
| AL121820.2 | TMEM178A | 0.618037087 | 4.27E-58 |
| AL121820.2 | RAB25 | 0.596532205 | 2.97E-53 |
| AL121820.2 | HOXB6 | 0.588154064 | 1.83E-51 |
| AL121820.2 | STAC2 | 0.587411661 | 2.62E-51 |
| LINC01213 | STK32A | 0.82767363 | 7.92E-137 |
| LINC01213 | GPD1L | 0.826990588 | 2.07E-136 |
| LINC01213 | OXCT1 | 0.780001141 | 2.10E-111 |
| LINC01213 | TMEM117 | 0.759347574 | 2.73E-102 |
| LINC01213 | KLHL3 | 0.758356687 | 7.08E-102 |
| LINC01213 | HCAR1 | 0.756459318 | 4.34E-101 |
| LINC01213 | C15orf59 | 0.731131723 | 3.13E-91 |
| LINC01213 | CACNA1D | 0.723497038 | 1.78E-88 |
| LINC01213 | NCALD | 0.721984708 | 6.08E-88 |
| LINC01213 | CFTR | 0.721017362 | 1.33E-87 |
| LINC00284 | ADTRP | 0.787333377 | 6.99E-115 |
| LINC00284 | FOXI2 | 0.690409765 | 1.55E-77 |
| LINC00284 | WFDC2 | 0.660871447 | 6.01E-69 |
| LINC00284 | UNC5D | 0.654536235 | 3.13E-67 |
| LINC00284 | NKX6-1 | 0.627358298 | 2.59E-60 |
| LINC00284 | RHBG | 0.604574275 | 5.07E-55 |
| LINC00284 | CD9 | 0.593103669 | 1.63E-52 |
| LINC00284 | CNGA1 | 0.591288081 | 3.97E-52 |
| LINC00284 | NIPAL1 | 0.586601468 | 3.87E-51 |
| LINC00284 | NTN1 | 0.563675327 | 1.59E-46 |
| AL157935.1 | SLC13A2 | 0.824927892 | 3.70E-135 |
| AL157935.1 | NMRK2 | 0.793195714 | 9.20E-118 |
| AL157935.1 | FAM102A | 0.713297379 | 6.13E-85 |
| AL157935.1 | IGFN1 | 0.686600204 | 2.26E-76 |
| AL157935.1 | SS18L1 | 0.679404037 | 3.22E-74 |
| AL157935.1 | ST6GALNAC2 | 0.651977446 | 1.50E-66 |
| AL157935.1 | SPAG5 | 0.640067733 | 1.84E-63 |
| AL157935.1 | DIO1 | 0.610092147 | 2.90E-56 |
| AL157935.1 | SCGB1D2 | 0.606319532 | 2.06E-55 |
| AL157935.1 | SSTR2 | 0.595797627 | 4.28E-53 |
| AL031710.1 | DEFB1 | 0.874983578 | 2.69E-171 |
| AL031710.1 | DUSP15 | 0.861130816 | 6.70E-160 |
| AL031710.1 | COX5A | 0.836558052 | 1.94E-142 |
| AL031710.1 | RHCG | 0.835651642 | 7.50E-142 |
| AL031710.1 | ATP6V1G3 | 0.833012364 | 3.68E-140 |
| AL031710.1 | CLCNKA | 0.830662957 | 1.11E-138 |
| AL031710.1 | BAG1 | 0.829304275 | 7.82E-138 |
| AL031710.1 | PVALB | 0.810730527 | 5.72E-127 |
| AL031710.1 | NDUFA6 | 0.805844322 | 2.61E-124 |
| AL031710.1 | PLLP | 0.799965941 | 3.30E-121 |
| AC012594.1 | ATP5EP2 | 0.850352381 | 7.43E-152 |
| AC012594.1 | NNAT | 0.834734517 | 2.93E-141 |
| AC012594.1 | TFF1 | 0.803258065 | 6.23E-123 |
| AC012594.1 | GATA3 | 0.795496516 | 6.42E-119 |
| AC012594.1 | FSIP1 | 0.784225282 | 2.16E-113 |
| AC012594.1 | CMTM1 | 0.744595268 | 2.51E-96 |
| AC012594.1 | RAD51B | 0.742776485 | 1.28E-95 |
| AC012594.1 | ESR1 | 0.739101076 | 3.29E-94 |
| AC012594.1 | ASIP | 0.730100035 | 7.46E-91 |
| AC012594.1 | ELOVL2 | 0.730042371 | 7.83E-91 |
| AC007342.4 | OXGR1 | 0.89196659 | 2.80E-187 |
| AC007342.4 | EPB41L4B | 0.882374702 | 6.01E-178 |
| AC007342.4 | HS6ST3 | 0.869509842 | 1.23E-166 |
| AC007342.4 | KLHL3 | 0.856543039 | 2.14E-156 |
| AC007342.4 | TRIM2 | 0.849925249 | 1.50E-151 |
| AC007342.4 | CLNK | 0.842400652 | 2.57E-146 |
| AC007342.4 | MYZAP | 0.841507801 | 1.03E-145 |
| AC007342.4 | HEPACAM2 | 0.84040835 | 5.62E-145 |
| AC007342.4 | PKIA | 0.82737715 | 1.20E-136 |
| AC007342.4 | TMEM116 | 0.826175156 | 6.51E-136 |
| AC013391.3 | LMO1 | 0.538326495 | 7.95E-42 |
| AC013391.3 | ZNF558 | 0.45433338 | 8.25E-29 |
| AC013391.3 | PBX4 | 0.446620741 | 8.72E-28 |
| AC013391.3 | RBCK1 | 0.430758506 | 9.24E-26 |
| AC013391.3 | PRELID3A | 0.417549645 | 3.74E-24 |
| AC013391.3 | ZBTB7C | 0.412928342 | 1.31E-23 |
| AC013391.3 | UCN | 0.410511658 | 2.51E-23 |
| AC013391.3 | IGFBP1 | 0.403426052 | 1.63E-22 |
| AC013391.3 | MTCP1 | 0.393773812 | 1.95E-21 |
| AC013391.3 | ADA | 0.392005583 | 3.05E-21 |
| AC007342.5 | OXGR1 | 0.902858109 | 5.25E-199 |
| AC007342.5 | HS6ST3 | 0.891299398 | 1.33E-186 |
| AC007342.5 | SLC16A7 | 0.854292998 | 1.01E-154 |
| AC007342.5 | CLNK | 0.850292725 | 8.20E-152 |
| AC007342.5 | MPPED2 | 0.835483244 | 9.64E-142 |
| AC007342.5 | EXPH5 | 0.829697846 | 4.46E-138 |
| AC007342.5 | EPB41L4B | 0.829087928 | 1.06E-137 |
| AC007342.5 | KLHL3 | 0.821590982 | 3.62E-133 |
| AC007342.5 | PPM1L | 0.81950359 | 6.05E-132 |
| AC007342.5 | RNF152 | 0.816893366 | 1.95E-130 |
| HOXB-AS3 | SMIM5 | 0.864466767 | 1.58E-162 |
| HOXB-AS3 | PACRG | 0.853840406 | 2.18E-154 |
| HOXB-AS3 | GADD45G | 0.784187782 | 2.25E-113 |
| HOXB-AS3 | CD9 | 0.778558467 | 9.78E-111 |
| HOXB-AS3 | ATP6V0D2 | 0.774756494 | 5.35E-109 |
| HOXB-AS3 | DEFB1 | 0.761029298 | 5.35E-103 |
| HOXB-AS3 | DUSP15 | 0.761008568 | 5.46E-103 |
| HOXB-AS3 | MAL | 0.758918529 | 4.13E-102 |
| HOXB-AS3 | MKKS | 0.754910813 | 1.88E-100 |
| HOXB-AS3 | TMEM61 | 0.754722885 | 2.25E-100 |
| AC016813.1 | ATP6V1B1 | 0.753435462 | 7.53E-100 |
| AC016813.1 | TMEM101 | 0.712153048 | 1.49E-84 |
| AC016813.1 | ATP6AP2 | 0.679156892 | 3.81E-74 |
| AC016813.1 | ATP6V1C2 | 0.675284643 | 5.16E-73 |
| AC016813.1 | GADD45G | 0.672400742 | 3.50E-72 |
| AC016813.1 | NAGS | 0.671749625 | 5.38E-72 |
| AC016813.1 | FOXI1 | 0.668389771 | 4.86E-71 |
| AC016813.1 | MAL | 0.664752961 | 5.08E-70 |
| AC016813.1 | BSND | 0.664058519 | 7.93E-70 |
| AC016813.1 | SLC9A4 | 0.653620164 | 5.50E-67 |
| LINC01606 | BAAT | 0.982823768 | 0 |
| LINC01606 | BZW2 | 0.969837438 | 0 |
| LINC01606 | IRX1 | 0.945630898 | 4.13E-264 |
| LINC01606 | GPBAR1 | 0.938598043 | 2.41E-250 |
| LINC01606 | PIGZ | 0.932451451 | 1.38E-239 |
| LINC01606 | ZNF114 | 0.916063709 | 3.04E-215 |
| LINC01606 | MYO7B | 0.9064958 | 3.10E-203 |
| LINC01606 | CAPN3 | 0.905585181 | 3.68E-202 |
| LINC01606 | BBS4 | 0.904705814 | 3.92E-201 |
| LINC01606 | LYPD6B | 0.903652289 | 6.48E-200 |
| AC104063.1 | HSP90B1 | 0.421738873 | 1.18E-24 |
| AC104063.1 | PRR13 | 0.418934319 | 2.55E-24 |
| AC104063.1 | SMKR1 | 0.416913217 | 4.45E-24 |
| AC104063.1 | C8orf46 | 0.412674333 | 1.40E-23 |
| AC104063.1 | SLC23A1 | 0.39807479 | 6.53E-22 |
| AC104063.1 | SIRPB1 | 0.395923835 | 1.13E-21 |
| AC104063.1 | SLC34A2 | 0.382052442 | 3.56E-20 |
| AC104063.1 | MGST1 | 0.376847888 | 1.25E-19 |
| AC104063.1 | MAOA | 0.366147575 | 1.52E-18 |
| AC104063.1 | ZCRB1 | 0.36048364 | 5.53E-18 |
| LINC01644 | FXYD3 | 0.756807629 | 3.11E-101 |
| LINC01644 | GALNT3 | 0.750329919 | 1.35E-98 |
| LINC01644 | AGR2 | 0.734921655 | 1.24E-92 |
| LINC01644 | TACSTD2 | 0.732886012 | 7.06E-92 |
| LINC01644 | SFTA2 | 0.724470494 | 8.00E-89 |
| LINC01644 | GABRP | 0.719732504 | 3.74E-87 |
| LINC01644 | TMPRSS4 | 0.714416342 | 2.55E-85 |
| LINC01644 | CNMD | 0.688196066 | 7.39E-77 |
| LINC01644 | MYO3B | 0.686951007 | 1.77E-76 |
| LINC01644 | SUSD4 | 0.652833327 | 8.91E-67 |
| AC002546.2 | HMBOX1 | 0.60168338 | 2.22E-54 |
| AC002546.2 | ZBTB16 | 0.598783241 | 9.61E-54 |
| AC002546.2 | ZBTB20 | 0.578818852 | 1.57E-49 |
| AC002546.2 | ANKAR | 0.570904963 | 6.10E-48 |
| AC002546.2 | TRIM13 | 0.568308417 | 1.99E-47 |
| AC002546.2 | GDAP2 | 0.568248788 | 2.04E-47 |
| AC002546.2 | BBIP1 | 0.566728629 | 4.05E-47 |
| AC002546.2 | ANKRD26 | 0.561037719 | 5.12E-46 |
| AC002546.2 | VAMP4 | 0.550709351 | 4.52E-44 |
| AC002546.2 | ZNF780B | 0.550632828 | 4.67E-44 |
| AC008060.1 | MATK | 0.455860945 | 5.14E-29 |
| AC008060.1 | CNTNAP5 | 0.417981938 | 3.32E-24 |
| AC008060.1 | TNMD | 0.409756418 | 3.07E-23 |
| AC008060.1 | KCNH2 | 0.330427975 | 3.39E-15 |
| AC008060.1 | NEFL | 0.325786944 | 8.58E-15 |
| AC008060.1 | SLC36A1 | 0.303373075 | 6.15E-13 |
| AC008060.1 | APBA2 | 0.278388485 | 4.75E-11 |
| AC008060.1 | OR2T10 | 0.267001661 | 2.99E-10 |
| AC008060.1 | SLC13A1 | 0.260775126 | 7.90E-10 |
| AC008060.1 | ORAI2 | 0.260691913 | 8.00E-10 |
| LINC01096 | CEMIP | 0.752038817 | 2.78E-99 |
| LINC01096 | MAP3K9 | 0.721732991 | 7.45E-88 |
| LINC01096 | NEK9 | 0.702882214 | 1.75E-81 |
| LINC01096 | CCDC77 | 0.700886674 | 7.74E-81 |
| LINC01096 | KISS1 | 0.69460133 | 7.68E-79 |
| LINC01096 | WNT5B | 0.69340941 | 1.81E-78 |
| LINC01096 | COL4A5 | 0.683708284 | 1.69E-75 |
| LINC01096 | GTF2F2 | 0.671110296 | 8.20E-72 |
| LINC01096 | AHSA1 | 0.659665645 | 1.28E-68 |
| LINC01096 | DIO2 | 0.657944592 | 3.78E-68 |
| AC092422.1 | SLC16A7 | 0.948165016 | 1.59E-269 |
| AC092422.1 | HS6ST3 | 0.901950121 | 5.62E-198 |
| AC092422.1 | MOB1B | 0.897325356 | 6.96E-193 |
| AC092422.1 | RNF152 | 0.895028747 | 1.91E-190 |
| AC092422.1 | SLC26A7 | 0.88929934 | 1.34E-184 |
| AC092422.1 | PPM1L | 0.883642222 | 3.92E-179 |
| AC092422.1 | PIK3C2G | 0.882562998 | 4.01E-178 |
| AC092422.1 | ZNF91 | 0.870915849 | 8.17E-168 |
| AC092422.1 | LIN7A | 0.867742156 | 3.53E-165 |
| AC092422.1 | EXPH5 | 0.864563814 | 1.32E-162 |
| LINC01300 | MMP19 | 0.599989798 | 5.23E-54 |
| LINC01300 | ARHGEF26 | 0.597430194 | 1.90E-53 |
| LINC01300 | C2orf40 | 0.575199427 | 8.46E-49 |
| LINC01300 | RASGRP2 | 0.540520546 | 3.23E-42 |
| LINC01300 | SNAI1 | 0.537660132 | 1.04E-41 |
| LINC01300 | DPT | 0.489651151 | 7.58E-34 |
| LINC01300 | PTGIR | 0.478655631 | 3.25E-32 |
| LINC01300 | LDB3 | 0.476905963 | 5.83E-32 |
| LINC01300 | ITGA7 | 0.474487542 | 1.30E-31 |
| LINC01300 | HIC1 | 0.473359548 | 1.89E-31 |
| SEMA3B-AS1 | PRRG2 | 0.707471027 | 5.49E-83 |
| SEMA3B-AS1 | TNFAIP8L3 | 0.662762841 | 1.81E-69 |
| SEMA3B-AS1 | DUSP15 | 0.659983355 | 1.05E-68 |
| SEMA3B-AS1 | CLCNKA | 0.659629832 | 1.31E-68 |
| SEMA3B-AS1 | BCAS4 | 0.658640534 | 2.44E-68 |
| SEMA3B-AS1 | C14orf2 | 0.653007561 | 8.01E-67 |
| SEMA3B-AS1 | FBXO2 | 0.651454718 | 2.07E-66 |
| SEMA3B-AS1 | CAPS | 0.650810904 | 3.06E-66 |
| SEMA3B-AS1 | SETMAR | 0.650706925 | 3.26E-66 |
| SEMA3B-AS1 | BAG1 | 0.638319144 | 5.09E-63 |
| AC022733.2 | PRSS22 | 0.620781361 | 9.67E-59 |
| AC022733.2 | USP43 | 0.575609594 | 7.00E-49 |
| AC022733.2 | ABCA4 | 0.565323287 | 7.61E-47 |
| AC022733.2 | C3orf52 | 0.553334771 | 1.47E-44 |
| AC022733.2 | CACNA1H | 0.538602447 | 7.10E-42 |
| AC022733.2 | PADI1 | 0.531150205 | 1.44E-40 |
| AC022733.2 | TACSTD2 | 0.519024182 | 1.66E-38 |
| AC022733.2 | SFN | 0.502697614 | 7.34E-36 |
| AC022733.2 | AGR2 | 0.499732597 | 2.14E-35 |
| AC022733.2 | LAMC2 | 0.482177462 | 9.89E-33 |
| AC104237.2 | OVCH2 | 0.830704489 | 1.05E-138 |
| AC104237.2 | THRSP | 0.505000187 | 3.17E-36 |
| AC104237.2 | LRRC39 | 0.498945603 | 2.84E-35 |
| AC104237.2 | PROZ | 0.49428369 | 1.49E-34 |
| AC104237.2 | MCCD1 | 0.474394128 | 1.34E-31 |
| AC104237.2 | WNT11 | 0.467991866 | 1.09E-30 |
| AC104237.2 | C16orf89 | 0.464823597 | 3.03E-30 |
| AC104237.2 | ME1 | 0.459212169 | 1.80E-29 |
| AC104237.2 | SLC2A12 | 0.452035479 | 1.68E-28 |
| AC104237.2 | NAPSA | 0.442017589 | 3.46E-27 |
| AC093281.2 | CCDC18 | 0.582769012 | 2.42E-50 |
| AC093281.2 | MS4A4E | 0.582697603 | 2.51E-50 |
| AC093281.2 | ATAD5 | 0.528100806 | 4.85E-40 |
| AC093281.2 | ZNF546 | 0.526707216 | 8.39E-40 |
| AC093281.2 | USP45 | 0.523606779 | 2.82E-39 |
| AC093281.2 | PHOSPHO1 | 0.522874897 | 3.75E-39 |
| AC093281.2 | ANKAR | 0.517729242 | 2.72E-38 |
| AC093281.2 | GPR17 | 0.516332097 | 4.63E-38 |
| AC093281.2 | OTULIN | 0.515202333 | 7.11E-38 |
| AC093281.2 | VAMP4 | 0.51455291 | 9.09E-38 |
| AC093904.4 | PLA2G4F | 0.826025211 | 8.03E-136 |
| AC093904.4 | C15orf59 | 0.820120693 | 2.64E-132 |
| AC093904.4 | EPN3 | 0.786246396 | 2.34E-114 |
| AC093904.4 | INPP5J | 0.74559717 | 1.02E-96 |
| AC093904.4 | HSD11B2 | 0.731483831 | 2.32E-91 |
| AC093904.4 | HCAR1 | 0.722146817 | 5.33E-88 |
| AC093904.4 | KLHL3 | 0.720901838 | 1.46E-87 |
| AC093904.4 | SMOC1 | 0.71818352 | 1.29E-86 |
| AC093904.4 | SYNGR3 | 0.716018124 | 7.22E-86 |
| AC093904.4 | PRKCD | 0.715393685 | 1.18E-85 |
| MIR3681HG | B4GALNT1 | 0.615098874 | 2.06E-57 |
| MIR3681HG | FMN1 | 0.596229184 | 3.45E-53 |
| MIR3681HG | INHA | 0.578832716 | 1.56E-49 |
| MIR3681HG | GPM6B | 0.573293174 | 2.04E-48 |
| MIR3681HG | FLNB | 0.571257748 | 5.19E-48 |
| MIR3681HG | MYO3B | 0.557713796 | 2.20E-45 |
| MIR3681HG | SLC12A3 | 0.544482622 | 6.25E-43 |
| MIR3681HG | PDE8B | 0.541769912 | 1.93E-42 |
| MIR3681HG | IGFBP2 | 0.530276139 | 2.04E-40 |
| MIR3681HG | DAPK2 | 0.529165678 | 3.18E-40 |
| LINC00322 | C8orf4 | 0.589670736 | 8.74E-52 |
| LINC00322 | CNMD | 0.558206219 | 1.78E-45 |
| LINC00322 | TMPRSS3 | 0.550796048 | 4.35E-44 |
| LINC00322 | KRT17 | 0.522955135 | 3.64E-39 |
| LINC00322 | ALDH1A2 | 0.502270081 | 8.57E-36 |
| LINC00322 | STAC2 | 0.498145752 | 3.79E-35 |
| LINC00322 | TACSTD2 | 0.496953498 | 5.80E-35 |
| LINC00322 | KLF5 | 0.483367971 | 6.60E-33 |
| LINC00322 | EHF | 0.482951182 | 7.60E-33 |
| LINC00322 | SFTA2 | 0.47678731 | 6.06E-32 |
| LINC00452 | EVI5L | 0.601170613 | 2.88E-54 |
| LINC00452 | SLC27A4 | 0.595077259 | 6.12E-53 |
| LINC00452 | PRKCD | 0.585115647 | 7.90E-51 |
| LINC00452 | PMAIP1 | 0.581658167 | 4.11E-50 |
| LINC00452 | CCDC151 | 0.579741237 | 1.02E-49 |
| LINC00452 | PARPBP | 0.578099629 | 2.19E-49 |
| LINC00452 | SYT17 | 0.552302881 | 2.29E-44 |
| LINC00452 | TJP3 | 0.531562195 | 1.22E-40 |
| LINC00452 | FAM189A2 | 0.530823017 | 1.64E-40 |
| LINC00452 | GPSM1 | 0.530645488 | 1.77E-40 |
| AC103563.7 | MAL | 0.872269316 | 5.85E-169 |
| AC103563.7 | ATP6V0D2 | 0.854801398 | 4.26E-155 |
| AC103563.7 | PACRG | 0.82545704 | 1.77E-135 |
| AC103563.7 | SMIM5 | 0.822903675 | 6.03E-134 |
| AC103563.7 | RALGPS1 | 0.810249684 | 1.05E-126 |
| AC103563.7 | SETMAR | 0.807446589 | 3.58E-125 |
| AC103563.7 | TBC1D1 | 0.799592396 | 5.15E-121 |
| AC103563.7 | PXK | 0.796649772 | 1.67E-119 |
| AC103563.7 | TMEM61 | 0.796513493 | 1.96E-119 |
| AC103563.7 | DUSP15 | 0.791594333 | 5.75E-117 |
| AC023421.1 | SLC14A2 | 0.908336794 | 1.92E-205 |
| AC023421.1 | PIK3C2G | 0.782069633 | 2.26E-112 |
| AC023421.1 | DGKI | 0.768315136 | 3.94E-106 |
| AC023421.1 | RNF152 | 0.766279968 | 3.03E-105 |
| AC023421.1 | MOB1B | 0.766146527 | 3.47E-105 |
| AC023421.1 | SAMD12 | 0.755876894 | 7.54E-101 |
| AC023421.1 | ZNF91 | 0.750168382 | 1.57E-98 |
| AC023421.1 | KIAA1456 | 0.749209714 | 3.80E-98 |
| AC023421.1 | SLC16A7 | 0.747644844 | 1.59E-97 |
| AC023421.1 | ZNF431 | 0.732557437 | 9.34E-92 |
| PCGEM1 | TMEM27 | 0.447190526 | 7.34E-28 |
| PCGEM1 | TNFAIP6 | 0.406083646 | 8.14E-23 |
| PCGEM1 | PTER | 0.393409273 | 2.14E-21 |
| PCGEM1 | WWP1 | 0.367598195 | 1.09E-18 |
| PCGEM1 | DNAJB9 | 0.367099595 | 1.22E-18 |
| PCGEM1 | DNAJC1 | 0.363596271 | 2.73E-18 |
| PCGEM1 | TMEM38B | 0.362817109 | 3.26E-18 |
| PCGEM1 | B3GALNT1 | 0.357748628 | 1.02E-17 |
| PCGEM1 | CCDC160 | 0.356730625 | 1.28E-17 |
| PCGEM1 | CRYZ | 0.35531391 | 1.75E-17 |
| AL592528.1 | TFF3 | 0.83374114 | 1.27E-140 |
| AL592528.1 | FAM180A | 0.796613069 | 1.74E-119 |
| AL592528.1 | TNNT3 | 0.711685861 | 2.15E-84 |
| AL592528.1 | SCNN1B | 0.677392373 | 1.25E-73 |
| AL592528.1 | SCNN1G | 0.673562589 | 1.62E-72 |
| AL592528.1 | IGF2 | 0.663385101 | 1.22E-69 |
| AL592528.1 | PTGS2 | 0.663077521 | 1.48E-69 |
| AL592528.1 | SCUBE1 | 0.661695302 | 3.57E-69 |
| AL592528.1 | RGS2 | 0.648072544 | 1.60E-65 |
| AL592528.1 | IL6 | 0.628291182 | 1.54E-60 |
| AL031123.1 | ATP6V0A4 | 0.885784318 | 3.62E-181 |
| AL031123.1 | FAM24B | 0.8674623 | 5.99E-165 |
| AL031123.1 | AQP6 | 0.860101282 | 4.20E-159 |
| AL031123.1 | LITAF | 0.822603302 | 9.10E-134 |
| AL031123.1 | TMEM213 | 0.81352092 | 1.60E-128 |
| AL031123.1 | NUPR2 | 0.813271005 | 2.21E-128 |
| AL031123.1 | TMEM61 | 0.78735464 | 6.83E-115 |
| AL031123.1 | BAALC | 0.767967863 | 5.59E-106 |
| AL031123.1 | TMEM55A | 0.75955931 | 2.22E-102 |
| AL031123.1 | ARHGAP18 | 0.746434444 | 4.78E-97 |
| LINC01485 | ITIH1 | 0.850273318 | 8.46E-152 |
| LINC01485 | C5 | 0.850102356 | 1.12E-151 |
| LINC01485 | SLC27A5 | 0.84959678 | 2.58E-151 |
| LINC01485 | AMBP | 0.849250419 | 4.54E-151 |
| LINC01485 | AGXT | 0.845883373 | 1.05E-148 |
| LINC01485 | GNMT | 0.845869894 | 1.08E-148 |
| LINC01485 | TTR | 0.844777849 | 6.12E-148 |
| LINC01485 | HPX | 0.844045203 | 1.95E-147 |
| LINC01485 | ITIH2 | 0.843951624 | 2.26E-147 |
| LINC01485 | APOH | 0.843849349 | 2.65E-147 |
| AL158068.2 | ZNF493 | 0.796181843 | 2.88E-119 |
| AL158068.2 | ZNF808 | 0.794994757 | 1.15E-118 |
| AL158068.2 | KIAA1328 | 0.78568445 | 4.35E-114 |
| AL158068.2 | DPH6 | 0.777551729 | 2.84E-110 |
| AL158068.2 | TNRC6B | 0.77296042 | 3.44E-108 |
| AL158068.2 | ABCA5 | 0.761324777 | 4.02E-103 |
| AL158068.2 | ZNF552 | 0.756092885 | 6.14E-101 |
| AL158068.2 | ZNF91 | 0.755700335 | 8.91E-101 |
| AL158068.2 | ZNF254 | 0.752788713 | 1.38E-99 |
| AL158068.2 | TMPPE | 0.745615057 | 1.00E-96 |
| AL353746.1 | LOXL3 | 0.676216241 | 2.77E-73 |
| AL353746.1 | LIPG | 0.665855971 | 2.50E-70 |
| AL353746.1 | SYP | 0.649172433 | 8.26E-66 |
| AL353746.1 | STMN2 | 0.648803546 | 1.03E-65 |
| AL353746.1 | CRYGS | 0.619069708 | 2.45E-58 |
| AL353746.1 | SMYD3 | 0.598145341 | 1.32E-53 |
| AL353746.1 | CHGB | 0.594579049 | 7.84E-53 |
| AL353746.1 | PTX3 | 0.581262808 | 4.95E-50 |
| AL353746.1 | FANCE | 0.569888451 | 9.70E-48 |
| AL353746.1 | ANKRD65 | 0.566345913 | 4.81E-47 |
| LINC01896 | SLC12A3 | 0.743163538 | 9.06E-96 |
| LINC01896 | TMPRSS3 | 0.72992675 | 8.63E-91 |
| LINC01896 | TFAP2B | 0.727907991 | 4.67E-90 |
| LINC01896 | PRR15L | 0.726506002 | 1.50E-89 |
| LINC01896 | STAC2 | 0.692578442 | 3.29E-78 |
| LINC01896 | KCNJ1 | 0.690790803 | 1.18E-77 |
| LINC01896 | FAM167A | 0.683341329 | 2.17E-75 |
| LINC01896 | KCNIP1 | 0.660991355 | 5.57E-69 |
| LINC01896 | FLNB | 0.660975685 | 5.62E-69 |
| LINC01896 | EHF | 0.650092762 | 4.73E-66 |
| AC022387.1 | SLC16A5 | 0.498571137 | 3.25E-35 |
| AC022387.1 | FZD7 | 0.421568564 | 1.23E-24 |
| AC022387.1 | ZDHHC3 | 0.412373264 | 1.52E-23 |
| AC022387.1 | HOXB6 | 0.395314346 | 1.32E-21 |
| AC022387.1 | TOX3 | 0.392614654 | 2.61E-21 |
| AC022387.1 | HOXB8 | 0.392009845 | 3.04E-21 |
| AC022387.1 | ADK | 0.387008606 | 1.06E-20 |
| AC022387.1 | KCNIP1 | 0.38263686 | 3.09E-20 |
| AC022387.1 | SERHL2 | 0.372267405 | 3.68E-19 |
| AC022387.1 | TMPRSS3 | 0.367742889 | 1.06E-18 |
| LINC01543 | PXK | 0.75077898 | 8.94E-99 |
| LINC01543 | SFTPB | 0.715236931 | 1.34E-85 |
| LINC01543 | DMRT2 | 0.703478506 | 1.12E-81 |
| LINC01543 | ATP6V0A4 | 0.702911261 | 1.71E-81 |
| LINC01543 | PLCG2 | 0.693395517 | 1.83E-78 |
| LINC01543 | FGF9 | 0.684217935 | 1.19E-75 |
| LINC01543 | SNTB1 | 0.681952166 | 5.65E-75 |
| LINC01543 | TMEM61 | 0.673331311 | 1.89E-72 |
| LINC01543 | KIT | 0.669185137 | 2.89E-71 |
| LINC01543 | TMEM213 | 0.660203101 | 9.16E-69 |
| C5orf64 | TMEM116 | 0.793670589 | 5.32E-118 |
| C5orf64 | ST3GAL6 | 0.771977893 | 9.47E-108 |
| C5orf64 | SETMAR | 0.769004042 | 1.96E-106 |
| C5orf64 | TNFAIP8L3 | 0.76750456 | 8.91E-106 |
| C5orf64 | SC5D | 0.755201686 | 1.43E-100 |
| C5orf64 | PFN2 | 0.7519389 | 3.05E-99 |
| C5orf64 | RHCG | 0.737507608 | 1.32E-93 |
| C5orf64 | FAM189A2 | 0.737325017 | 1.55E-93 |
| C5orf64 | ATP6V1G3 | 0.73116944 | 3.03E-91 |
| C5orf64 | DUSP15 | 0.727833584 | 4.97E-90 |
| AC092142.1 | SERHL2 | 0.669326339 | 2.64E-71 |
| AC092142.1 | AQP6 | 0.640627021 | 1.33E-63 |
| AC092142.1 | FAM24B | 0.63945655 | 2.63E-63 |
| AC092142.1 | PDE6G | 0.621034066 | 8.43E-59 |
| AC092142.1 | SDHAF4 | 0.614087168 | 3.53E-57 |
| AC092142.1 | C20orf196 | 0.598184854 | 1.30E-53 |
| AC092142.1 | BAALC | 0.594654325 | 7.55E-53 |
| AC092142.1 | TMIE | 0.592913195 | 1.79E-52 |
| AC092142.1 | COX7A2L | 0.592043495 | 2.74E-52 |
| AC092142.1 | MED31 | 0.591466496 | 3.64E-52 |
| LINC02302 | SLC14A2 | 0.674282802 | 1.01E-72 |
| LINC02302 | CEP78 | 0.649127018 | 8.49E-66 |
| LINC02302 | FAM221A | 0.647138064 | 2.81E-65 |
| LINC02302 | GATA3 | 0.638696247 | 4.09E-63 |
| LINC02302 | NNAT | 0.610144199 | 2.82E-56 |
| LINC02302 | FOXP1 | 0.605467199 | 3.20E-55 |
| LINC02302 | SCUBE2 | 0.60481936 | 4.47E-55 |
| LINC02302 | STRBP | 0.585550648 | 6.41E-51 |
| LINC02302 | TBC1D9 | 0.580536706 | 6.98E-50 |
| LINC02302 | ADCK1 | 0.572893431 | 2.45E-48 |
| AL355102.5 | NNAT | 0.699510535 | 2.14E-80 |
| AL355102.5 | RAD51B | 0.595377318 | 5.28E-53 |
| AL355102.5 | FAM221A | 0.586809127 | 3.50E-51 |
| AL355102.5 | CMTM1 | 0.586367559 | 4.33E-51 |
| AL355102.5 | ATP5EP2 | 0.581892734 | 3.67E-50 |
| AL355102.5 | FSIP1 | 0.557159112 | 2.80E-45 |
| AL355102.5 | TFF1 | 0.550942595 | 4.09E-44 |
| AL355102.5 | GATA3 | 0.550669788 | 4.60E-44 |
| AL355102.5 | BDKRB2 | 0.533990419 | 4.62E-41 |
| AL355102.5 | ESR1 | 0.529877784 | 2.40E-40 |
| LINC01055 | ZNF165 | 0.700214549 | 1.27E-80 |
| LINC01055 | KLK1 | 0.699397409 | 2.33E-80 |
| LINC01055 | ESRRB | 0.65994197 | 1.08E-68 |
| LINC01055 | LINC00890 | 0.655536087 | 1.69E-67 |
| LINC01055 | LDLR | 0.65076641 | 3.14E-66 |
| LINC01055 | CCRL2 | 0.623053468 | 2.80E-59 |
| LINC01055 | SH3GL2 | 0.587257996 | 2.82E-51 |
| LINC01055 | TMEM52B | 0.526353548 | 9.64E-40 |
| LINC01055 | NPHS1 | 0.49334798 | 2.08E-34 |
| LINC01055 | CA10 | 0.490308294 | 6.03E-34 |
| LINC00982 | CLCNKA | 0.88055029 | 2.89E-176 |
| LINC00982 | FOXI1 | 0.871262763 | 4.17E-168 |
| LINC00982 | SMIM5 | 0.823448199 | 2.86E-134 |
| LINC00982 | MAL | 0.816512999 | 3.22E-130 |
| LINC00982 | ATP6V1B1 | 0.811860698 | 1.35E-127 |
| LINC00982 | FAM189A2 | 0.802655672 | 1.29E-122 |
| LINC00982 | FA2H | 0.80237456 | 1.82E-122 |
| LINC00982 | ATP6V0D2 | 0.801689745 | 4.16E-122 |
| LINC00982 | PLA2G4F | 0.79915086 | 8.71E-121 |
| LINC00982 | C15orf59 | 0.798962942 | 1.09E-120 |
| AL049555.1 | FAM83B | 0.866042084 | 8.56E-164 |
| AL049555.1 | TRIM2 | 0.792825381 | 1.41E-117 |
| AL049555.1 | SLC16A7 | 0.792073 | 3.33E-117 |
| AL049555.1 | CLNK | 0.783626061 | 4.17E-113 |
| AL049555.1 | MPPED2 | 0.779001976 | 6.10E-111 |
| AL049555.1 | SIM1 | 0.771828429 | 1.10E-107 |
| AL049555.1 | ESRP1 | 0.769000179 | 1.97E-106 |
| AL049555.1 | KLHL3 | 0.764222828 | 2.34E-104 |
| AL049555.1 | RALGPS1 | 0.762994675 | 7.85E-104 |
| AL049555.1 | SACM1L | 0.758999601 | 3.82E-102 |
| AC005082.1 | MYO3B | 0.753807591 | 5.31E-100 |
| AC005082.1 | KCTD1 | 0.68924004 | 3.54E-77 |
| AC005082.1 | TMCO4 | 0.684840874 | 7.70E-76 |
| AC005082.1 | EHF | 0.681217778 | 9.35E-75 |
| AC005082.1 | VTCN1 | 0.657293521 | 5.67E-68 |
| AC005082.1 | HOXB8 | 0.652445033 | 1.13E-66 |
| AC005082.1 | ESRP1 | 0.649271322 | 7.78E-66 |
| AC005082.1 | B4GALNT1 | 0.648726933 | 1.08E-65 |
| AC005082.1 | GRHL2 | 0.62848692 | 1.38E-60 |
| AC005082.1 | FLNB | 0.627656092 | 2.19E-60 |
| AC128688.2 | C3orf52 | 0.838142979 | 1.78E-143 |
| AC128688.2 | PRSS22 | 0.755679974 | 9.09E-101 |
| AC128688.2 | USP43 | 0.749522572 | 2.85E-98 |
| AC128688.2 | SLC25A29 | 0.702167738 | 2.99E-81 |
| AC128688.2 | PADI1 | 0.67592772 | 3.36E-73 |
| AC128688.2 | SFN | 0.661468282 | 4.12E-69 |
| AC128688.2 | CACNA1H | 0.644735489 | 1.18E-64 |
| AC128688.2 | SOCS1 | 0.613373783 | 5.16E-57 |
| AC128688.2 | LCN12 | 0.608728682 | 5.91E-56 |
| AC128688.2 | VNN1 | 0.584475181 | 1.07E-50 |
| THRB-AS1 | SLC26A7 | 0.968419455 | 0 |
| THRB-AS1 | HS6ST3 | 0.913896082 | 2.11E-212 |
| THRB-AS1 | TRIM2 | 0.913462759 | 7.65E-212 |
| THRB-AS1 | SLC16A7 | 0.913416723 | 8.77E-212 |
| THRB-AS1 | CCDC68 | 0.912175674 | 3.37E-210 |
| THRB-AS1 | PKIA | 0.909921885 | 2.22E-207 |
| THRB-AS1 | C1orf168 | 0.900476409 | 2.51E-196 |
| THRB-AS1 | TSPAN5 | 0.899286914 | 5.16E-195 |
| THRB-AS1 | TMEM116 | 0.896418699 | 6.49E-192 |
| THRB-AS1 | CLNK | 0.892767646 | 4.26E-188 |
| GDNF-AS1 | KRT20 | 0.803394111 | 5.28E-123 |
| GDNF-AS1 | GAL3ST3 | 0.779348126 | 4.22E-111 |
| GDNF-AS1 | CTSK | 0.770943144 | 2.73E-107 |
| GDNF-AS1 | SLC36A2 | 0.746516503 | 4.44E-97 |
| GDNF-AS1 | PEG3 | 0.644223904 | 1.60E-64 |
| GDNF-AS1 | NECAB1 | 0.641452192 | 8.19E-64 |
| GDNF-AS1 | OMG | 0.639747963 | 2.22E-63 |
| GDNF-AS1 | TMEM171 | 0.62269198 | 3.41E-59 |
| GDNF-AS1 | ORC5 | 0.612959883 | 6.42E-57 |
| GDNF-AS1 | MRO | 0.609479253 | 4.00E-56 |
| AC079062.1 | CCNE1 | 0.773430828 | 2.12E-108 |
| AC079062.1 | HPX | 0.763535015 | 4.61E-104 |
| AC079062.1 | AHSG | 0.758792209 | 4.66E-102 |
| AC079062.1 | SLC27A5 | 0.756675691 | 3.53E-101 |
| AC079062.1 | ITIH2 | 0.75640588 | 4.56E-101 |
| AC079062.1 | SERPINC1 | 0.755679116 | 9.09E-101 |
| AC079062.1 | ADH4 | 0.755670195 | 9.17E-101 |
| AC079062.1 | GNMT | 0.755512825 | 1.06E-100 |
| AC079062.1 | APOA1 | 0.755484976 | 1.09E-100 |
| AC079062.1 | AGXT | 0.754963651 | 1.79E-100 |
| AL731557.1 | SLC18A2 | 0.799442668 | 6.15E-121 |
| AL731557.1 | FAM24B | 0.751862896 | 3.27E-99 |
| AL731557.1 | AQP6 | 0.706672965 | 1.01E-82 |
| AL731557.1 | ZDHHC3 | 0.631443169 | 2.61E-61 |
| AL731557.1 | LITAF | 0.623962476 | 1.70E-59 |
| AL731557.1 | GADD45G | 0.621138969 | 7.96E-59 |
| AL731557.1 | ATP6V0A4 | 0.588015481 | 1.95E-51 |
| AL731557.1 | NUPR2 | 0.578013167 | 2.28E-49 |
| AL731557.1 | SFTPB | 0.572633425 | 2.76E-48 |
| AL731557.1 | ARHGAP18 | 0.571010092 | 5.82E-48 |
| LY86-AS1 | AQP6 | 0.75281854 | 1.34E-99 |
| LY86-AS1 | FAM24B | 0.738502156 | 5.56E-94 |
| LY86-AS1 | ATP6V0A4 | 0.726908777 | 1.07E-89 |
| LY86-AS1 | NUPR2 | 0.697912087 | 6.92E-80 |
| LY86-AS1 | LITAF | 0.694420146 | 8.76E-79 |
| LY86-AS1 | FAM221A | 0.678454763 | 6.12E-74 |
| LY86-AS1 | COQ3 | 0.675405697 | 4.76E-73 |
| LY86-AS1 | BAALC | 0.662159677 | 2.66E-69 |
| LY86-AS1 | MCOLN2 | 0.660497607 | 7.61E-69 |
| LY86-AS1 | TMEM55A | 0.65541711 | 1.82E-67 |
| LINC01230 | CLCNKA | 0.862286929 | 8.38E-161 |
| LINC01230 | DHRS7 | 0.840061761 | 9.57E-145 |
| LINC01230 | FAM189A2 | 0.830690373 | 1.07E-138 |
| LINC01230 | DMRT2 | 0.822579819 | 9.39E-134 |
| LINC01230 | WBSCR17 | 0.814107005 | 7.48E-129 |
| LINC01230 | GABARAPL1 | 0.813652937 | 1.35E-128 |
| LINC01230 | DUSP15 | 0.807814807 | 2.26E-125 |
| LINC01230 | TMEM38A | 0.807691369 | 2.63E-125 |
| LINC01230 | SPINK1 | 0.805421741 | 4.40E-124 |
| LINC01230 | ATP6V0D2 | 0.804835061 | 9.06E-124 |
| MCF2L-AS1 | ATP6V0D2 | 0.90647645 | 3.26E-203 |
| MCF2L-AS1 | ABHD3 | 0.889777989 | 4.49E-185 |
| MCF2L-AS1 | FOXI1 | 0.868956099 | 3.53E-166 |
| MCF2L-AS1 | ERP27 | 0.848745144 | 1.04E-150 |
| MCF2L-AS1 | INPP5J | 0.837570744 | 4.23E-143 |
| MCF2L-AS1 | SYT17 | 0.836961511 | 1.06E-142 |
| MCF2L-AS1 | NTN1 | 0.829895816 | 3.36E-138 |
| MCF2L-AS1 | SETMAR | 0.829760942 | 4.07E-138 |
| MCF2L-AS1 | FAM189A2 | 0.826452186 | 4.42E-136 |
| MCF2L-AS1 | CLNK | 0.82301325 | 5.19E-134 |
| AP000844.2 | NTM | 0.658405641 | 2.83E-68 |
| AP000844.2 | SHC1 | 0.54222155 | 1.60E-42 |
| AP000844.2 | CPA4 | 0.541837665 | 1.88E-42 |
| AP000844.2 | IGLL5 | 0.530792673 | 1.66E-40 |
| AP000844.2 | TSHZ2 | 0.490108439 | 6.47E-34 |
| AP000844.2 | APLP1 | 0.478560285 | 3.35E-32 |
| AP000844.2 | HSH2D | 0.464610525 | 3.24E-30 |
| AP000844.2 | ECM1 | 0.455003736 | 6.70E-29 |
| AP000844.2 | MEI1 | 0.451274335 | 2.12E-28 |
| AP000844.2 | FCHO1 | 0.442471655 | 3.02E-27 |
| AC069029.1 | ZBTB20 | 0.900736139 | 1.29E-196 |
| AC069029.1 | GPR18 | 0.890515345 | 8.22E-186 |
| AC069029.1 | TNRC6B | 0.870813405 | 9.97E-168 |
| AC069029.1 | ZDHHC21 | 0.831052902 | 6.35E-139 |
| AC069029.1 | ZNF587B | 0.830076056 | 2.59E-138 |
| AC069029.1 | ZNF717 | 0.826596332 | 3.61E-136 |
| AC069029.1 | ZNF808 | 0.824520586 | 6.51E-135 |
| AC069029.1 | SHPRH | 0.817344743 | 1.07E-130 |
| AC069029.1 | GDAP2 | 0.813032033 | 3.00E-128 |
| AC069029.1 | ZNF493 | 0.801574916 | 4.78E-122 |
| AL118522.1 | FAM83B | 0.765301461 | 8.04E-105 |
| AL118522.1 | GPD1L | 0.729760177 | 9.93E-91 |
| AL118522.1 | KCNK15 | 0.710984054 | 3.70E-84 |
| AL118522.1 | ERBB4 | 0.705100945 | 3.31E-82 |
| AL118522.1 | SCIN | 0.681135353 | 9.89E-75 |
| AL118522.1 | ESRP1 | 0.680829614 | 1.22E-74 |
| AL118522.1 | MREG | 0.675443279 | 4.64E-73 |
| AL118522.1 | BMPR1B | 0.669390083 | 2.53E-71 |
| AL118522.1 | FAM169A | 0.668230485 | 5.38E-71 |
| AL118522.1 | STK32A | 0.667024645 | 1.18E-70 |
| CHL1-AS2 | CHL1 | 0.792735373 | 1.56E-117 |
| CHL1-AS2 | LCN2 | 0.612419904 | 8.54E-57 |
| CHL1-AS2 | SOSTDC1 | 0.598077036 | 1.37E-53 |
| CHL1-AS2 | TPK1 | 0.547270296 | 1.94E-43 |
| CHL1-AS2 | TMEM252 | 0.531831355 | 1.10E-40 |
| CHL1-AS2 | UPK1B | 0.529190503 | 3.15E-40 |
| CHL1-AS2 | EPHA1 | 0.501571397 | 1.10E-35 |
| CHL1-AS2 | ABCB8 | 0.483116458 | 7.19E-33 |
| CHL1-AS2 | SYNE4 | 0.475346398 | 9.79E-32 |
| CHL1-AS2 | TM7SF2 | 0.464322096 | 3.56E-30 |
| LINC00645 | SLC2A12 | 0.773209522 | 2.66E-108 |
| LINC00645 | ASB9 | 0.670805747 | 1.00E-71 |
| LINC00645 | C16orf89 | 0.615008259 | 2.16E-57 |
| LINC00645 | ESRRG | 0.588762563 | 1.36E-51 |
| LINC00645 | SLC9A4 | 0.567945391 | 2.34E-47 |
| LINC00645 | TMEM101 | 0.554643205 | 8.35E-45 |
| LINC00645 | WIPI2 | 0.543528766 | 9.30E-43 |
| LINC00645 | PFKFB2 | 0.54296175 | 1.18E-42 |
| LINC00645 | GMPR | 0.533866439 | 4.86E-41 |
| LINC00645 | EGF | 0.528367522 | 4.36E-40 |
| BX322234.2 | KLHDC7B | 0.729249477 | 1.52E-90 |
| BX322234.2 | PROM2 | 0.718841155 | 7.65E-87 |
| BX322234.2 | C8orf46 | 0.694229691 | 1.00E-78 |
| BX322234.2 | HPD | 0.600760505 | 3.54E-54 |
| BX322234.2 | SIRPB1 | 0.593168871 | 1.57E-52 |
| BX322234.2 | SLC39A4 | 0.573993573 | 1.48E-48 |
| BX322234.2 | PLTP | 0.556664224 | 3.48E-45 |
| BX322234.2 | KCNS1 | 0.5354914 | 2.52E-41 |
| BX322234.2 | LRRC43 | 0.503781507 | 4.95E-36 |
| BX322234.2 | PF4V1 | 0.479139257 | 2.76E-32 |
| AC122710.3 | CCDC18 | 0.567365924 | 3.04E-47 |
| AC122710.3 | ATAD5 | 0.525115987 | 1.57E-39 |
| AC122710.3 | MS4A4E | 0.507385049 | 1.32E-36 |
| AC122710.3 | ULK4 | 0.492593943 | 2.71E-34 |
| AC122710.3 | C8orf44 | 0.491730313 | 3.67E-34 |
| AC122710.3 | ZNF546 | 0.481138084 | 1.41E-32 |
| AC122710.3 | ANKAR | 0.473748674 | 1.66E-31 |
| AC122710.3 | HELLS | 0.472408423 | 2.58E-31 |
| AC122710.3 | OTULIN | 0.466604015 | 1.71E-30 |
| AC122710.3 | MFSD4B | 0.464045934 | 3.89E-30 |
| GATA3-AS1 | FXYD4 | 0.889474696 | 9.00E-185 |
| GATA3-AS1 | C4orf48 | 0.884403691 | 7.49E-180 |
| GATA3-AS1 | ZNF593 | 0.882082593 | 1.12E-177 |
| GATA3-AS1 | NDUFA13 | 0.867370212 | 7.12E-165 |
| GATA3-AS1 | PTGER1 | 0.85279175 | 1.28E-153 |
| GATA3-AS1 | EVA1B | 0.849088142 | 5.93E-151 |
| GATA3-AS1 | CCDC85B | 0.847069993 | 1.57E-149 |
| GATA3-AS1 | C21orf33 | 0.844115907 | 1.74E-147 |
| GATA3-AS1 | SMIM22 | 0.843501729 | 4.59E-147 |
| GATA3-AS1 | UQCC3 | 0.837124121 | 8.29E-143 |
| C15orf56 | ATP6V0D2 | 0.783514193 | 4.71E-113 |
| C15orf56 | SYT17 | 0.777335758 | 3.57E-110 |
| C15orf56 | RHBG | 0.775478244 | 2.52E-109 |
| C15orf56 | TMEM30B | 0.771015613 | 2.54E-107 |
| C15orf56 | DMRT2 | 0.769796372 | 8.80E-107 |
| C15orf56 | EPB41L4B | 0.766820125 | 1.77E-105 |
| C15orf56 | ERP27 | 0.764514785 | 1.75E-104 |
| C15orf56 | SMIM5 | 0.761367631 | 3.85E-103 |
| C15orf56 | SLC29A2 | 0.756372028 | 4.71E-101 |
| C15orf56 | TBC1D1 | 0.742098335 | 2.34E-95 |
| AC093390.1 | CXCL6 | 0.708662719 | 2.21E-83 |
| AC093390.1 | MMP25 | 0.656572987 | 8.87E-68 |
| AC093390.1 | CXCL1 | 0.57978032 | 9.97E-50 |
| AC093390.1 | FCGR3B | 0.575486813 | 7.41E-49 |
| AC093390.1 | PF4V1 | 0.560892904 | 5.46E-46 |
| AC093390.1 | CSF3R | 0.4847653 | 4.09E-33 |
| AC093390.1 | SAA4 | 0.404945363 | 1.10E-22 |
| AC093390.1 | S100A8 | 0.396563152 | 9.61E-22 |
| AC093390.1 | BAIAP3 | 0.396072643 | 1.09E-21 |
| AC093390.1 | TMPRSS3 | 0.390023427 | 5.00E-21 |
| LINC01544 | GNPNAT1 | 0.881665107 | 2.73E-177 |
| LINC01544 | FOXI2 | 0.83580538 | 5.97E-142 |
| LINC01544 | ERP27 | 0.832565632 | 7.08E-140 |
| LINC01544 | UNC5D | 0.832406209 | 8.93E-140 |
| LINC01544 | RHBG | 0.814537515 | 4.27E-129 |
| LINC01544 | CD9 | 0.814357677 | 5.40E-129 |
| LINC01544 | NKX6-1 | 0.814159566 | 6.98E-129 |
| LINC01544 | ABHD3 | 0.799020177 | 1.02E-120 |
| LINC01544 | ADTRP | 0.794525205 | 1.98E-118 |
| LINC01544 | ADAMTSL1 | 0.754316937 | 3.29E-100 |
| LINC02268 | ECM1 | 0.553523914 | 1.35E-44 |
| LINC02268 | SLC17A9 | 0.545026663 | 4.98E-43 |
| LINC02268 | MX2 | 0.507492982 | 1.27E-36 |
| LINC02268 | NPEPL1 | 0.469708465 | 6.25E-31 |
| LINC02268 | PYCR1 | 0.468204184 | 1.02E-30 |
| LINC02268 | APOL2 | 0.44797773 | 5.78E-28 |
| LINC02268 | ARL4C | 0.445114351 | 1.37E-27 |
| LINC02268 | QSOX1 | 0.437930473 | 1.16E-26 |
| LINC02268 | DNTTIP1 | 0.437368145 | 1.36E-26 |
| LINC02268 | RIPK2 | 0.435200762 | 2.57E-26 |
| AC009061.1 | SCG3 | 0.828685471 | 1.89E-137 |
| AC009061.1 | SHISA2 | 0.792183286 | 2.94E-117 |
| AC009061.1 | PLA2G4F | 0.78523336 | 7.15E-114 |
| AC009061.1 | HSD11B2 | 0.778500971 | 1.04E-110 |
| AC009061.1 | GOLM1 | 0.771461584 | 1.61E-107 |
| AC009061.1 | SLC26A4 | 0.765318481 | 7.91E-105 |
| AC009061.1 | INPP5J | 0.760082923 | 1.34E-102 |
| AC009061.1 | TRIM50 | 0.756680291 | 3.51E-101 |
| AC009061.1 | NHLRC4 | 0.733552267 | 4.00E-92 |
| AC009061.1 | ACSS3 | 0.722030525 | 5.86E-88 |
| ZNF350-AS1 | MYO3B | 0.757812129 | 1.19E-101 |
| ZNF350-AS1 | VTCN1 | 0.710254465 | 6.50E-84 |
| ZNF350-AS1 | CASP14 | 0.661660475 | 3.65E-69 |
| ZNF350-AS1 | KCNJ1 | 0.64204539 | 5.78E-64 |
| ZNF350-AS1 | FLNB | 0.638385454 | 4.90E-63 |
| ZNF350-AS1 | GALNT3 | 0.636178904 | 1.75E-62 |
| ZNF350-AS1 | TFAP2B | 0.63197457 | 1.93E-61 |
| ZNF350-AS1 | ANKS1A | 0.627191975 | 2.84E-60 |
| ZNF350-AS1 | HOXB8 | 0.626360898 | 4.51E-60 |
| ZNF350-AS1 | SUSD4 | 0.624170393 | 1.51E-59 |
| LINC01234 | IL20RB | 0.574398984 | 1.23E-48 |
| LINC01234 | CARD11 | 0.553425126 | 1.41E-44 |
| LINC01234 | GALNT2 | 0.539004122 | 6.02E-42 |
| LINC01234 | TUBA1C | 0.527122362 | 7.13E-40 |
| LINC01234 | C20orf24 | 0.515990855 | 5.27E-38 |
| LINC01234 | SLC39A11 | 0.502302505 | 8.47E-36 |
| LINC01234 | MTMR4 | 0.492342432 | 2.96E-34 |
| LINC01234 | PON2 | 0.490620738 | 5.41E-34 |
| LINC01234 | RIPK2 | 0.487909651 | 1.39E-33 |
| LINC01234 | EFNA5 | 0.480381198 | 1.82E-32 |
| LINC01655 | ATP5EP2 | 0.925101395 | 5.53E-228 |
| LINC01655 | TFF1 | 0.894949384 | 2.32E-190 |
| LINC01655 | FSIP1 | 0.875517834 | 9.19E-172 |
| LINC01655 | CMTM1 | 0.833929484 | 9.60E-141 |
| LINC01655 | ASIP | 0.829757481 | 4.09E-138 |
| LINC01655 | CRABP2 | 0.822628948 | 8.78E-134 |
| LINC01655 | ELOVL2 | 0.815077702 | 2.11E-129 |
| LINC01655 | RAD51B | 0.811237863 | 3.00E-127 |
| LINC01655 | FASN | 0.809892055 | 1.66E-126 |
| LINC01655 | NNAT | 0.807691435 | 2.63E-125 |
| AL121821.1 | GRHL2 | 0.65035047 | 4.05E-66 |
| AL121821.1 | GATA3 | 0.608330142 | 7.28E-56 |
| AL121821.1 | NNAT | 0.599270319 | 7.52E-54 |
| AL121821.1 | NRXN3 | 0.589402764 | 9.96E-52 |
| AL121821.1 | GRIP1 | 0.58737025 | 2.67E-51 |
| AL121821.1 | TJP3 | 0.582554338 | 2.68E-50 |
| AL121821.1 | ATP5EP2 | 0.55213736 | 2.45E-44 |
| AL121821.1 | FAM221A | 0.548960234 | 9.50E-44 |
| AL121821.1 | SCUBE2 | 0.547659742 | 1.65E-43 |
| AL121821.1 | TBC1D9 | 0.547598847 | 1.69E-43 |
| AL359633.2 | FAM24B | 0.835382944 | 1.12E-141 |
| AL359633.2 | AQP6 | 0.823107135 | 4.56E-134 |
| AL359633.2 | SLC18A2 | 0.81733475 | 1.09E-130 |
| AL359633.2 | LITAF | 0.806104746 | 1.89E-124 |
| AL359633.2 | ATP6V0A4 | 0.79719098 | 8.84E-120 |
| AL359633.2 | TMEM213 | 0.784874336 | 1.06E-113 |
| AL359633.2 | VAT1L | 0.748405697 | 7.94E-98 |
| AL359633.2 | PLCG2 | 0.737395165 | 1.46E-93 |
| AL359633.2 | KIT | 0.725301405 | 4.04E-89 |
| AL359633.2 | SFTPB | 0.712868623 | 8.56E-85 |
| AC010255.1 | ATP5EP2 | 0.812797897 | 4.06E-128 |
| AC010255.1 | GATA3 | 0.780295892 | 1.53E-111 |
| AC010255.1 | TFF1 | 0.774879317 | 4.70E-109 |
| AC010255.1 | NNAT | 0.753148545 | 9.85E-100 |
| AC010255.1 | FSIP1 | 0.740755467 | 7.68E-95 |
| AC010255.1 | RAD51B | 0.730387024 | 5.86E-91 |
| AC010255.1 | CMTM1 | 0.729673962 | 1.07E-90 |
| AC010255.1 | HARBI1 | 0.724755648 | 6.33E-89 |
| AC010255.1 | CRABP2 | 0.715347228 | 1.23E-85 |
| AC010255.1 | ASIP | 0.707782092 | 4.33E-83 |
| AC061975.7 | ESRP1 | 0.778344952 | 1.23E-110 |
| AC061975.7 | GRHL2 | 0.771910824 | 1.01E-107 |
| AC061975.7 | KCTD1 | 0.751992347 | 2.90E-99 |
| AC061975.7 | MECOM | 0.737566114 | 1.26E-93 |
| AC061975.7 | TMEM30B | 0.732866412 | 7.18E-92 |
| AC061975.7 | ERMP1 | 0.725886219 | 2.50E-89 |
| AC061975.7 | CPXM2 | 0.705547891 | 2.36E-82 |
| AC061975.7 | SCNN1A | 0.700436775 | 1.08E-80 |
| AC061975.7 | SCIN | 0.689639914 | 2.67E-77 |
| AC061975.7 | MREG | 0.683606131 | 1.81E-75 |
| AL445647.1 | STX1B | 0.294277327 | 3.14E-12 |
| AL445647.1 | ZNF888 | 0.254637579 | 2.01E-09 |
| AL445647.1 | PTER | 0.251292537 | 3.30E-09 |
| AL445647.1 | ACRBP | 0.233194702 | 4.32E-08 |
| AL445647.1 | WDR55 | 0.226723262 | 1.03E-07 |
| AL445647.1 | NEU3 | 0.225010642 | 1.29E-07 |
| AL445647.1 | HNF4G | 0.222935494 | 1.70E-07 |
| AL445647.1 | PLPPR5 | 0.222716932 | 1.75E-07 |
| AL445647.1 | SLC6A6 | 0.215617401 | 4.33E-07 |
| AL445647.1 | COG7 | 0.214424498 | 5.03E-07 |
| AL137793.1 | SLC9A4 | 0.863050345 | 2.10E-161 |
| AL137793.1 | TMEM101 | 0.840135785 | 8.54E-145 |
| AL137793.1 | ATP6V1C2 | 0.827461789 | 1.07E-136 |
| AL137793.1 | SLC43A1 | 0.826231583 | 6.01E-136 |
| AL137793.1 | ATP6V1B1 | 0.796924682 | 1.21E-119 |
| AL137793.1 | BSND | 0.774905612 | 4.58E-109 |
| AL137793.1 | NAGS | 0.748104431 | 1.05E-97 |
| AL137793.1 | GMPR | 0.746076122 | 6.61E-97 |
| AL137793.1 | ESRRG | 0.744404573 | 2.98E-96 |
| AL137793.1 | CA5B | 0.727761067 | 5.28E-90 |
| AC104237.3 | OVCH2 | 0.876876406 | 5.86E-173 |
| AC104237.3 | NAPSA | 0.459676171 | 1.56E-29 |
| AC104237.3 | BEX3 | 0.368563966 | 8.73E-19 |
| AC104237.3 | MCCD1 | 0.367419085 | 1.14E-18 |
| AC104237.3 | TDGF1 | 0.360059578 | 6.08E-18 |
| AC104237.3 | RASD1 | 0.359304512 | 7.20E-18 |
| AC104237.3 | BEX2 | 0.337059011 | 8.72E-16 |
| AC104237.3 | THRSP | 0.333422041 | 1.84E-15 |
| AC104237.3 | SLC2A12 | 0.311003565 | 1.50E-13 |
| AC104237.3 | BEX4 | 0.304012765 | 5.47E-13 |
| AC025431.1 | ZNF165 | 0.674207412 | 1.06E-72 |
| AC025431.1 | LINC00890 | 0.62576474 | 6.27E-60 |
| AC025431.1 | FAM189A2 | 0.621805424 | 5.53E-59 |
| AC025431.1 | KLK1 | 0.617028067 | 7.35E-58 |
| AC025431.1 | ALDH1A2 | 0.61409837 | 3.51E-57 |
| AC025431.1 | CCRL2 | 0.597599908 | 1.74E-53 |
| AC025431.1 | SDCBP2 | 0.590213285 | 6.71E-52 |
| AC025431.1 | ESRP1 | 0.581223143 | 5.04E-50 |
| AC025431.1 | GABARAPL1 | 0.572533847 | 2.89E-48 |
| AC025431.1 | ABHD17C | 0.56687378 | 3.79E-47 |
| AC243585.1 | MCOLN2 | 0.797194945 | 8.79E-120 |
| AC243585.1 | AQP6 | 0.794725358 | 1.57E-118 |
| AC243585.1 | PLCG2 | 0.78568473 | 4.35E-114 |
| AC243585.1 | ENAM | 0.747573562 | 1.70E-97 |
| AC243585.1 | RAB40B | 0.728167674 | 3.76E-90 |
| AC243585.1 | FGF9 | 0.727921795 | 4.62E-90 |
| AC243585.1 | FZD7 | 0.709547673 | 1.12E-83 |
| AC243585.1 | MAPRE3 | 0.694557841 | 7.93E-79 |
| AC243585.1 | BLNK | 0.692833087 | 2.74E-78 |
| AC243585.1 | CRTAC1 | 0.692706116 | 3.00E-78 |
| AC005064.1 | OXCT1 | 0.499772214 | 2.11E-35 |
| AC005064.1 | PLCL2 | 0.488761182 | 1.03E-33 |
| AC005064.1 | FAM83B | 0.474151124 | 1.45E-31 |
| AC005064.1 | GPD1L | 0.467798666 | 1.16E-30 |
| AC005064.1 | ADRB2 | 0.464717027 | 3.13E-30 |
| AC005064.1 | ATP10D | 0.463910681 | 4.06E-30 |
| AC005064.1 | PLA2G4A | 0.460442502 | 1.22E-29 |
| AC005064.1 | BMPR1B | 0.459216137 | 1.80E-29 |
| AC005064.1 | TBC1D1 | 0.454173841 | 8.67E-29 |
| AC005064.1 | GPAM | 0.442246433 | 3.23E-27 |
| LINC01187 | CFTR | 0.906214314 | 6.67E-203 |
| LINC01187 | ERP27 | 0.903226019 | 2.00E-199 |
| LINC01187 | CLNK | 0.895721947 | 3.56E-191 |
| LINC01187 | TBC1D1 | 0.893602951 | 5.86E-189 |
| LINC01187 | PXK | 0.883649023 | 3.86E-179 |
| LINC01187 | DMRT2 | 0.883050118 | 1.41E-178 |
| LINC01187 | NTN1 | 0.8820884 | 1.11E-177 |
| LINC01187 | ATP6V0D2 | 0.873852484 | 2.57E-170 |
| LINC01187 | KIT | 0.865952926 | 1.01E-163 |
| LINC01187 | FGF9 | 0.864982495 | 6.10E-163 |
| AL807761.4 | ZBTB20 | 0.918576434 | 1.23E-218 |
| AL807761.4 | ZNF808 | 0.868569036 | 7.38E-166 |
| AL807761.4 | TNRC6B | 0.864079021 | 3.21E-162 |
| AL807761.4 | ZNF460 | 0.852692379 | 1.51E-153 |
| AL807761.4 | GDAP2 | 0.836258817 | 3.03E-142 |
| AL807761.4 | GPR18 | 0.826281359 | 5.61E-136 |
| AL807761.4 | SHPRH | 0.819993384 | 3.14E-132 |
| AL807761.4 | INTS6 | 0.819442922 | 6.57E-132 |
| AL807761.4 | ZNF780B | 0.811323763 | 2.69E-127 |
| AL807761.4 | ZNF587B | 0.810364726 | 9.10E-127 |
| AP000757.1 | MUC20 | 0.928571657 | 2.62E-233 |
| AP000757.1 | FAM189A2 | 0.900805895 | 1.08E-196 |
| AP000757.1 | ERP27 | 0.891374775 | 1.12E-186 |
| AP000757.1 | DHRS7 | 0.887543475 | 7.18E-183 |
| AP000757.1 | RHBG | 0.874247033 | 1.17E-170 |
| AP000757.1 | NTN1 | 0.835483748 | 9.63E-142 |
| AP000757.1 | FOXI1 | 0.833049742 | 3.49E-140 |
| AP000757.1 | PIP | 0.832718928 | 5.66E-140 |
| AP000757.1 | TBC1D1 | 0.831311405 | 4.37E-139 |
| AP000757.1 | ATP6V0D2 | 0.824624774 | 5.63E-135 |
| AC114811.2 | DGKI | 0.945964626 | 8.27E-265 |
| AC114811.2 | PIK3C2G | 0.920879299 | 7.64E-222 |
| AC114811.2 | HS6ST3 | 0.910304188 | 7.46E-208 |
| AC114811.2 | ZNF431 | 0.906737617 | 1.60E-203 |
| AC114811.2 | BMPR1B | 0.902909361 | 4.59E-199 |
| AC114811.2 | RNF152 | 0.891475102 | 8.85E-187 |
| AC114811.2 | SLC16A7 | 0.891386674 | 1.09E-186 |
| AC114811.2 | PPM1L | 0.890089532 | 2.20E-185 |
| AC114811.2 | LIN7A | 0.881011316 | 1.09E-176 |
| AC114811.2 | MOB1B | 0.876906437 | 5.51E-173 |
| AL020994.2 | KLHL3 | 0.848173359 | 2.63E-150 |
| AL020994.2 | TMPRSS2 | 0.828953089 | 1.29E-137 |
| AL020994.2 | TMEM255A | 0.825933087 | 9.13E-136 |
| AL020994.2 | GABARAPL1 | 0.818966955 | 1.24E-131 |
| AL020994.2 | CA10 | 0.807159007 | 5.12E-125 |
| AL020994.2 | GPD1L | 0.793316578 | 8.00E-118 |
| AL020994.2 | DIRAS1 | 0.781477926 | 4.29E-112 |
| AL020994.2 | FOXI1 | 0.780519627 | 1.20E-111 |
| AL020994.2 | PNPLA8 | 0.757872165 | 1.13E-101 |
| AL020994.2 | TRIM50 | 0.755509509 | 1.07E-100 |
| AL049870.3 | PXK | 0.75170177 | 3.80E-99 |
| AL049870.3 | DMRT2 | 0.734465263 | 1.83E-92 |
| AL049870.3 | KIT | 0.69628035 | 2.28E-79 |
| AL049870.3 | BAALC | 0.688355743 | 6.60E-77 |
| AL049870.3 | PCNX4 | 0.686923664 | 1.80E-76 |
| AL049870.3 | ATP6V0A4 | 0.668413042 | 4.78E-71 |
| AL049870.3 | ATP6V0D2 | 0.666095227 | 2.14E-70 |
| AL049870.3 | PACRG | 0.662130777 | 2.71E-69 |
| AL049870.3 | SMIM5 | 0.661332382 | 4.49E-69 |
| AL049870.3 | SYT17 | 0.656240877 | 1.09E-67 |
| AC012653.2 | NNAT | 0.849198843 | 4.95E-151 |
| AC012653.2 | ATP5EP2 | 0.83064029 | 1.15E-138 |
| AC012653.2 | TFF1 | 0.809750287 | 1.98E-126 |
| AC012653.2 | GATA3 | 0.787316879 | 7.12E-115 |
| AC012653.2 | FSIP1 | 0.771949531 | 9.75E-108 |
| AC012653.2 | ASIP | 0.7335505 | 4.01E-92 |
| AC012653.2 | CMTM1 | 0.732655227 | 8.59E-92 |
| AC012653.2 | CRABP2 | 0.729785525 | 9.72E-91 |
| AC012653.2 | RAD51B | 0.728826631 | 2.17E-90 |
| AC012653.2 | FASN | 0.724011103 | 1.17E-88 |
| AC148477.4 | C15orf59 | 0.873808263 | 2.81E-170 |
| AC148477.4 | PRKCD | 0.853974724 | 1.74E-154 |
| AC148477.4 | HCN2 | 0.845946085 | 9.53E-149 |
| AC148477.4 | HSD11B2 | 0.845710103 | 1.39E-148 |
| AC148477.4 | FAM189A2 | 0.840743265 | 3.36E-145 |
| AC148477.4 | CCDC151 | 0.836338139 | 2.69E-142 |
| AC148477.4 | TRIM50 | 0.834703378 | 3.06E-141 |
| AC148477.4 | DUSP15 | 0.832391328 | 9.12E-140 |
| AC148477.4 | INPP5J | 0.831083958 | 6.08E-139 |
| AC148477.4 | SCNN1A | 0.828986397 | 1.23E-137 |
| AC087071.1 | NXPH4 | 0.347498851 | 9.63E-17 |
| AC087071.1 | ANKRD2 | 0.330831577 | 3.12E-15 |
| AC087071.1 | RIC3 | 0.292155108 | 4.56E-12 |
| AC087071.1 | STPG3 | 0.289249965 | 7.56E-12 |
| AC087071.1 | ZNF662 | 0.27195874 | 1.36E-10 |
| AC087071.1 | SLC16A5 | 0.267449709 | 2.79E-10 |
| AC087071.1 | TMPRSS3 | 0.258206162 | 1.17E-09 |
| AC087071.1 | PLEKHA4 | 0.258008456 | 1.21E-09 |
| AC087071.1 | OBSL1 | 0.256841197 | 1.44E-09 |
| AC087071.1 | ZNF853 | 0.256836751 | 1.44E-09 |
| AC079296.1 | SLC14A2 | 0.810561297 | 7.09E-127 |
| AC079296.1 | FAM221A | 0.737912978 | 9.29E-94 |
| AC079296.1 | NNAT | 0.706020236 | 1.65E-82 |
| AC079296.1 | HS6ST3 | 0.664915285 | 4.58E-70 |
| AC079296.1 | CEP78 | 0.659197636 | 1.72E-68 |
| AC079296.1 | ZNF552 | 0.649537438 | 6.62E-66 |
| AC079296.1 | PIK3C2G | 0.649361261 | 7.37E-66 |
| AC079296.1 | KIAA1328 | 0.647113428 | 2.85E-65 |
| AC079296.1 | EXPH5 | 0.640862574 | 1.16E-63 |
| AC079296.1 | ZNF431 | 0.640420138 | 1.50E-63 |
| AC023421.2 | BMPR1B | 0.951213341 | 2.06E-276 |
| AC023421.2 | PIK3C2G | 0.923615772 | 8.78E-226 |
| AC023421.2 | DGKI | 0.902279059 | 2.39E-198 |
| AC023421.2 | PKIA | 0.889944945 | 3.06E-185 |
| AC023421.2 | HS6ST3 | 0.888044528 | 2.32E-183 |
| AC023421.2 | ATP8B4 | 0.885499011 | 6.79E-181 |
| AC023421.2 | ZDHHC23 | 0.884921347 | 2.42E-180 |
| AC023421.2 | RNF152 | 0.882529708 | 4.31E-178 |
| AC023421.2 | ZNF431 | 0.872483661 | 3.84E-169 |
| AC023421.2 | CLNK | 0.869330301 | 1.73E-166 |
