# Supplementary material for: Genomic Instability Promotes the Progression of Clear Cell Renal Cell Carcinoma Through Influencing the Immune Microenvironment
Source: Front Genet. 2021 Oct 12;12:706661. doi: 10.3389/fgene.2021.706661 (PMC8546190; doi:10.3389/fgene.2021.706661)
Supplement: Supplementary file 6 [file Data_Sheet_2.ZIP › Raw Data List.docx]

1. Raw Data 1: Gene mutation matrix
2. Raw Data 2: Gene mutation count
3. Raw Data 3: mRNA expression matrix
4. Raw Data 4: LncRNA expression matrix
5. Raw Data 5: List of differentially expressed genes
6. Raw Data 6: Sample clustering file
7. Raw Data 7: Training set file
8. Raw Data 8: Validation set file
9. Raw Data 9: GSE53757 dataset expression file
10. Raw Data 10: TCGA-KIRC TMB file
11. Raw Data 11: CIBERSORT immune cell filter
12. Raw Data 12: EPIC immune cell filter
13. Raw Data 13: Patients clinical data
