# Supplementary figures and images for: Genomic Instability Promotes the Progression of Clear Cell Renal Cell Carcinoma Through Influencing the Immune Microenvironment
Source: Front Genet. 2021 Oct 12;12:706661. doi: 10.3389/fgene.2021.706661 (PMC8546190; doi:10.3389/fgene.2021.706661)

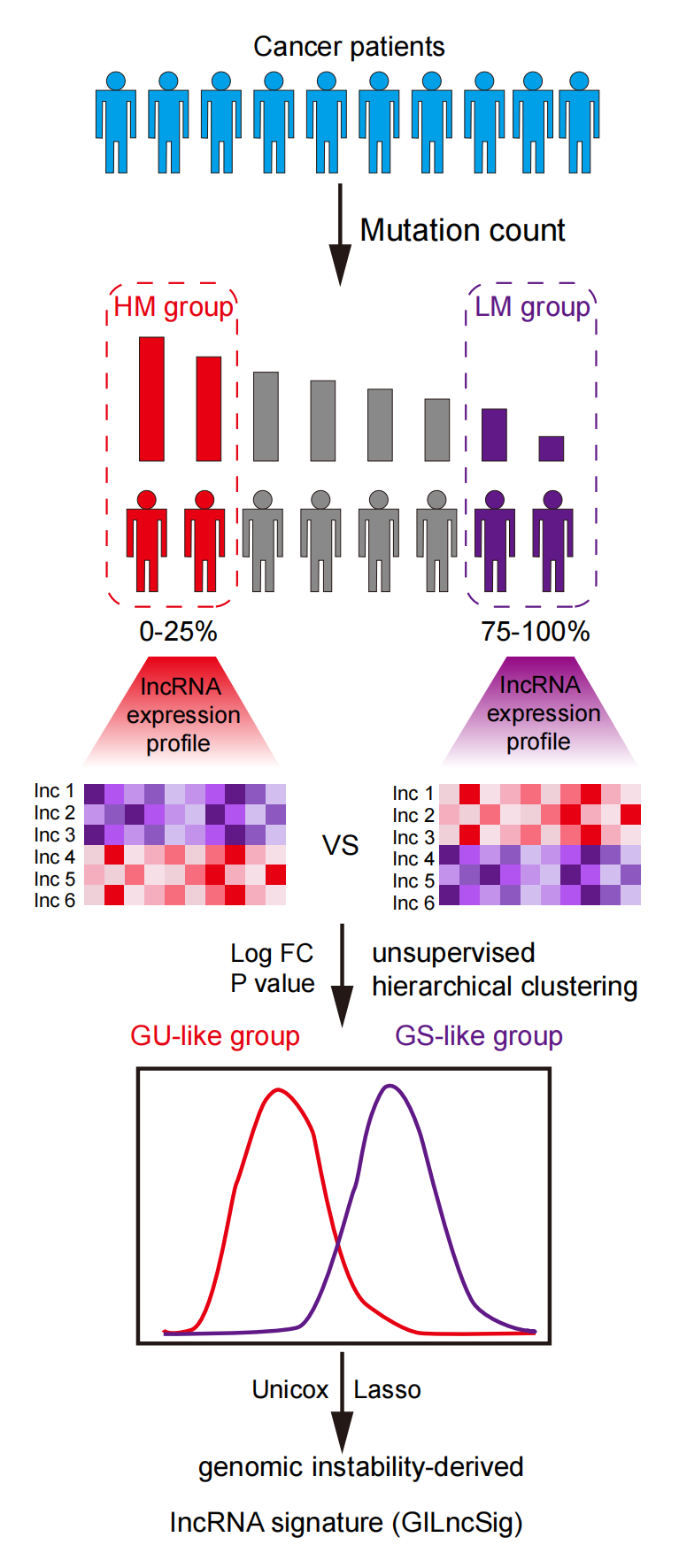

Supplement: Supplementary Figure 1 — The flow diagram of the study. [file Image_1.tif]

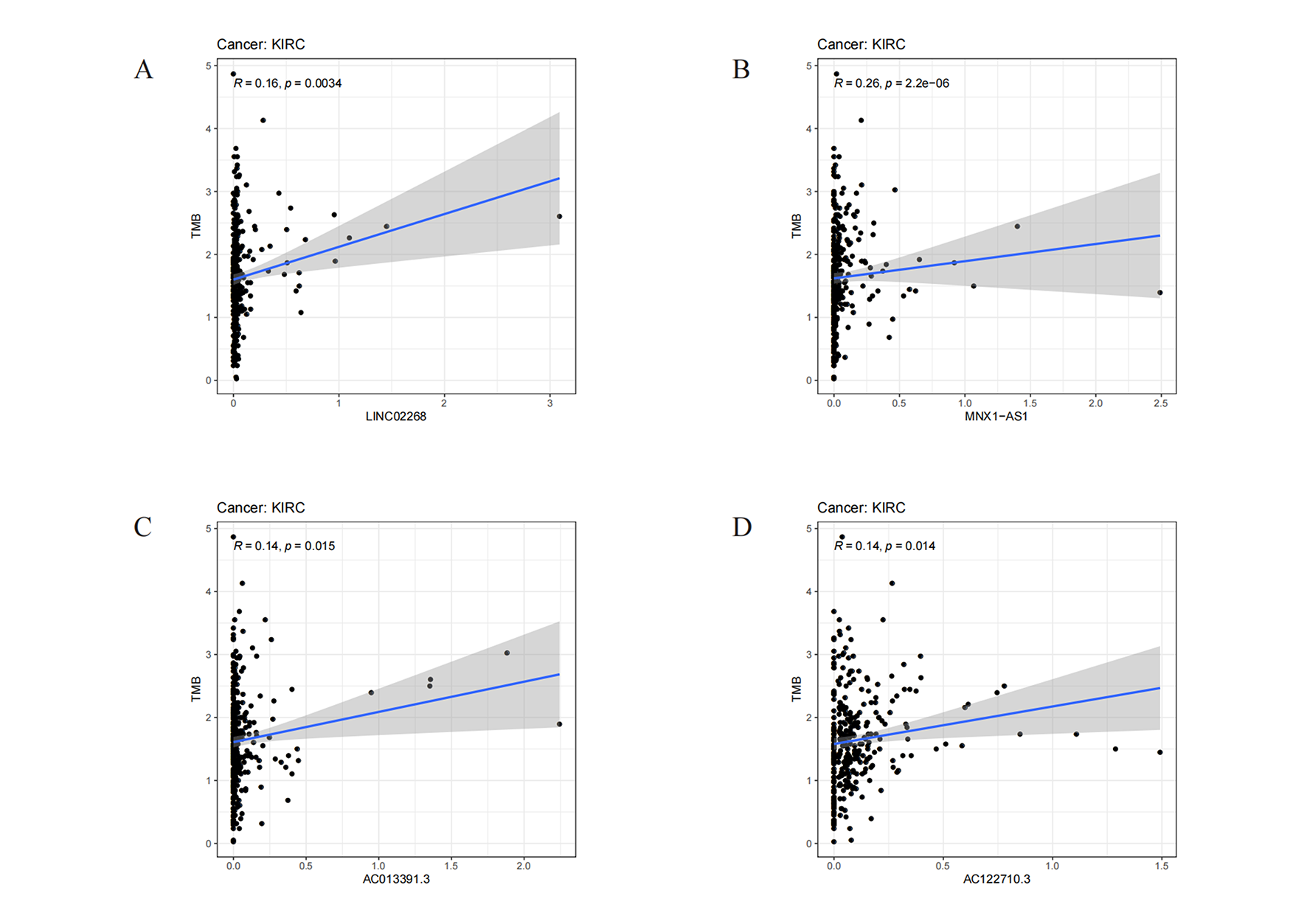

Supplement: Supplementary Figure 2 — Identification the correlation between genomic instability-derived lncRNAs and Tumor mutation burden (TMB). (A) The correlation between LINC02268 and TMB. (B) The correlation between MNX1-AS1 and TMB. (C) The correlation between AC013391.3 and TMB. (D) The correlation between AC122710.3 and TMB. [file Image_2.TIF]

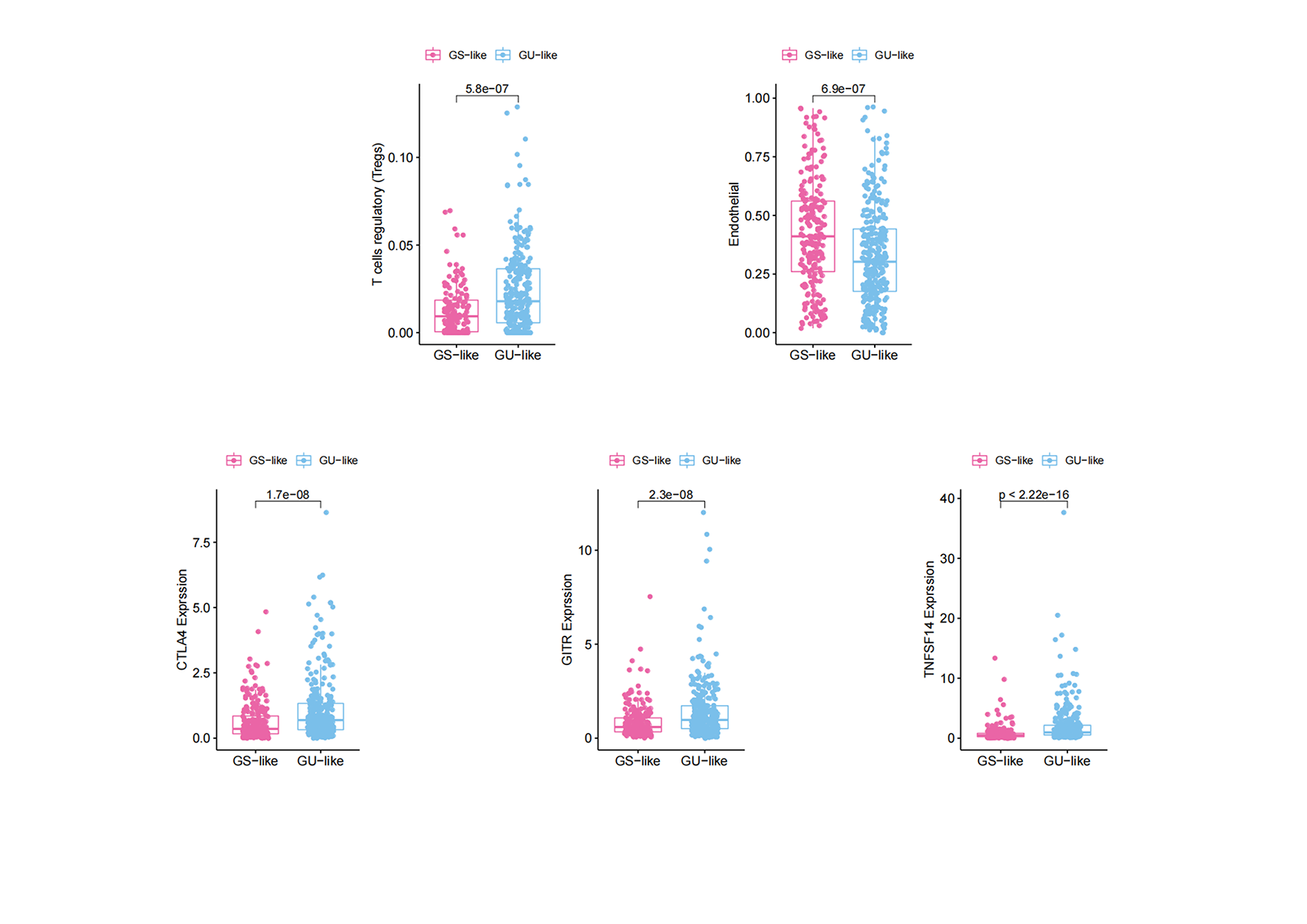

Supplement: Supplementary Figure 3 — Identification the expression level of the immune-associated features in GU-like group and GS-like group. The expression level of T cells regulatory (Tregs), CTLA4, GITR, and TNFSF14 in the GU-like group was significantly higher than that in the GS-like group while the expression level of Endothelial in the GU-like group was significantly lower than that in the GS-like group. [file Image_3.TIF]
